# Chalcogen bonding in the solid-state structures of 1,3-bis(benzimidazoliumyl)benzene-based chalcogen-bonding donors

**DOI:** 10.1107/S2053229622011536

**Published:** 2023-01-11

**Authors:** Tim Steinke, Elric Engelage, Stefan M. Huber

**Affiliations:** a Fakultät für Chemie und Biochemie, Ruhr-Universität Bochum, Universitätsstrasse 150, Bochum, 44801, Germany; University of Sheffield, United Kingdom

**Keywords:** chalcogen bonding, Lewis acid, crystal structure, sigma hole, intermolecular interactions, benzimidazolium

## Abstract

1,3-Bis(benzimidazoliumyl)benzene-based chalcogen-bonding catalysts were previously successfully applied in different benchmark reactions. In one of those examples, *i.e.* the activation of quinolines, sulfur- and selenium-based chalcogen-bonding catalysts showed comparable properties, which is unexpected, as the selenium-con­taining catalysts should show superior catalytic properties due to the increased polarizability of selenium compared to sulfur. Herein, we present four crystal structures of the respective 1,3-bis­(benzimidazoliumyl)benzene-based chalcogen-bonding catalyst con­taining sulfur (**3^S^**) and selenium (**3^Se^**, three forms) as Lewis acidic centres. The sulfur-con­taining catalyst shows weaker chalcogen bonding compared to its selenium analogue, as well as anion–π interactions. The selenium-based analogues, on the other hand, show stronger chalcogen-bonding motifs compared to the sulfur equivalent, de­pen­ding on the crystallization conditions, but in every case, the intermolecular interactions are comparable in strength. Other interactions, such as hydrogen bonding and anion–π, were also observed, but in the latter case, the interaction distances are longer compared to those of the sulfur-based equivalent. The solid-state structures could not further explain the high catalytic activity of the sulfur-con­taining catalysts. Therefore, a comparison of their σ-hole depths from density functional theory (DFT) gas-phase calculations was performed, which are again in line with the previously found properties in the solid-state structures.

## Abstract 2

1,3-Bis(benzimidazoliumyl)benzene-based chalcogen-bonding catalysts were crystallized and their intermolecular interactions investigated. Depending on the chalcogen employed and the crystallization conditions, different binding motifs were found. With sulfur centres as Lewis acidic interaction sites, only weak chalcogen bonding was observed. For selenium-based compounds, however, different bidentate coordination motifs were found between the chalcogen-bonding donor and the respective counter-ions.

## Introduction

Chalcogen bonding (ChB) (Aakeroy *et al.*, 2019 ▸) is defined as the attractive interaction of Lewis acidic chalcogen centres with Lewis bases and is closely related to hydrogen bonding (Doyle & Jacobsen, 2007 ▸) and halogen bonding (Cavallo *et al.*, 2016 ▸). However, chalcogen bonding is the least investigated interaction in this group. This interaction can be explained by three major contributions, again similar to hydrogen and halogen bonding: (i) charge transfer, described by an n→σ*-orbital interaction (Mulliken, 1952 ▸); (ii) electrostatic attraction of the lone pair of the Lewis base with an electropositive region (σ-hole) at the chalcogen (Rosenfield *et al.*, 1977 ▸); (iii) dispersion (Bleiholder *et al.*, 2006 ▸). In general, chalcogen bonding is a more directional interaction compared to hydro­gen bonding, which is explained by the absence of filled *p*-orbitals at hydrogen and by the fact that the σ*-orbital of the *R*—H bond is comprised of the 1*s* orbital on the hydrogen side. Furthermore, the interaction site is not limited to a single atom and can vary between tellurium, selenium and sulfur, which consequently allows the fine tuning of the interaction strength. Less electronegativity and easier polarization of the chalcogen leads to an increased anisotropic electron distribution and thus an enlarged electropositive region (σ-hole), as well as to a lower-lying σ*-orbital of the *R*—Ch bond. Hence, the strength of the chalcogen bonding increases with the atomic number of chalcogen (S < Se < Te). Another difference to hydrogen and halogen bonding is the second substituent located at the interacting atom. This interaction was observed in several solid-state systems, such as Ebselen, a synthetic organo­selenium drug (Dupont *et al.*, 1990 ▸) and di­phenyl diselenide with iodine (Kubiniok *et al.*, 1988 ▸). Intensive studies (Bleiholder *et al.*, 2006 ▸) and applications of the Gleiter group used chalcogen–chalcogen interactions to synthesize porous materials, such as nanotubes (Werz *et al.*, 2002 ▸), that incorporated other organic molecules like solvents (Werz *et al.*, 2004 ▸). In parallel, first applications in solution appeared as intramolecular chalcogen bonding was applied for the rigidification of intermediates to induce chirality (Fujita *et al.*, 1994 ▸; Wirth, 1995 ▸; Tiecco *et al.*, 2002 ▸). Moving from intra- to intermolecular interactions in solution, pioneering works in the field of anion recognition *via* chalcogen bonding have been performed by Gabbaï and co-workers (Zhao & Gabbaï, 2010 ▸) using charged chalcogen-bonding donors, and by Taylor and co-workers with neutral chalcogen-bonding donors (Garrett *et al.*, 2015 ▸). Extending the field of anion recognition to anion transport, the group of Matile introduced neutral dithieno­thio­phene (DTT) chalcogen-bonding donors (Benz *et al.*, 2016 ▸), which were also employed in oligomeric fashion as transmembrane transporters in further studies (Macchione *et al.*, 2018 ▸). Furthermore, with the DTT motif, the first application of chalcogen bonding in organocatalysis was presented by the same group (Benz *et al.*, 2017 ▸). Catalytic amounts of these compounds successfully activate quinolines for their reductive hydrogenation to 1,2,3,4-tetra­hydro­quinoline derivatives. In halogen-bonding organocatalysis, cationic donors were often markedly more active than neutral donors (Jungbauer & Huber, 2015 ▸). There­fore, our group reported the use of cationic chalcogen-bonding donors, which con­tained the more Lewis acidic chalcogen selenium on a 1,3-bis­(benz­imidazoliumyl)benzene scaffold (Wonner *et al.*, 2017*a* ▸). These catalysts were used in stoichiometric (Wonner *et al.*, 2017*a* ▸) and catalytic (Wonner *et al.*, 2017*b* ▸) activations of carbon–halide bonds. We also successfully demonstrated the catalytic activity of the same catalyst and the sulfur congener in the above-mentioned hydrogenation reaction (Wonner *et al.*, 2019*b* ▸).

The use of cationic selenium-based Lewis acids was further extended by Wang and co-workers in the activation of carbonyl functionalities (Wang *et al.*, 2019 ▸). As the development of chalcogen-bonding catalysts moved on, tellurium-based catalysts were introduced and applied in the activation of *trans*-β-nitro­styrene (Wonner *et al.*, 2019*a* ▸) and crotono­phenone (Wonner *et al.*, 2020 ▸) for Michael reactions, as well as in the activation of a carbon–chloride bond (Steinke *et al.*, 2021 ▸) and of imines (Steinke *et al.*, 2022 ▸). Lately, hypervalent tellurium-based chalcogen-bonding donors were introduced in organocatalysis (Weiss *et al.*, 2021 ▸; Zhou & Gabbaï, 2021 ▸). Herein, we present four crystal structures of 1,3-bis­(benz­imid­azoliumyl)benzene-based chalcogen-bonding donors with selenium and sulfur as Lewis acidic centres. Monodentate, bidentate and biaxial intermolecular chalcogen-bonding inter­actions are observed in these solid-state structures, which are further discussed in terms of previously observed catalytic activity. In addition, we performed DFT calculations to analyze chalcogen bonding in the absence of packing effects observed in the crystal structures of the catalyst system at hand.

## Experimental

### General remarks

Commercially available chemicals were purchased from ABCR, Alfa Aesar, Carbolution, Merck, ChemPur, Sigma–Aldrich, Roth or VWR and were used without further purification. All experiments were carried out under an inert gas atmosphere with dry solvents and flame-dried glassware using standard Schlenk techniques. Dry di­chloro­methane, di­ethyl ether and tetra­hydro­furan were received from an MBRAUN MB SPS-800 solvent purification system. Solvents were distilled and dried over a 4 Å molecular sieve and finally dried on an Alox column. Other dry solvents were dried with flame-dried 4 Å molecular sieve. A Karl Fischer Titroline 7500KF trace with Honeywell Hydranal–Coulomat AD solution was used to determine residual water. Merck thin-layer chromatography (TLC) aluminium sheets (silica gel 60, F254) were used for TLC analysis. Substances were detected by fluorescence under UV light (wavelength λ = 254 nm). Column chromatography was performed with silica gel (grain size 0.04–0.063 mm, Macherey–Nagel-Si60) and distilled solvents. The solvents used as eluents with the corresponding *R*_F_ values are listed for the corresponding experiments. ^1^H and ^13^C NMR spectra were recorded at room tem­per­ature with a Bruker AVIII 300 spectrometer. ^19^F NMR spectra were recorded at room tem­per­ature with a Bruker DPX-250 spectrometer and were measured proton decoupled if not further noted. ESI–MS spectra were recorded with a Bruker Esquire 6000, with the compounds dissolved in aceto­nitrile or methanol. EI–MS spectra were recorded on a Jeol AccuTOF. FT–IR spectra were recorded with a Shimadzu IR Affinity-IS spectrometer equipped with a Specac-Quest ATR module. The crystal structures were analysed on a Rigaku Synergy dual-source device, with a Cu microfocus sealed tube (Cu *K*α) using mirror monochromators and a HyPix-6000HE Hybrid photon coun­ting X-ray detector. The crystals were mounted in Hampton CryoLoops using GE/Bayer silicone grease. Data were re­corded and reduced using *Crysalis PRO* software (Rigaku OD, 2018 ▸). The structure was solved using *WinGX* (Farrugia, 2012 ▸) in combination with *SHELXT* (Sheldrick, 2015*a* ▸) and refined with *shelXle* (Hübschle *et al.*, 2011 ▸) and *SHELXL* (Sheldrick, 2015*b* ▸).

### Synthesis of known compounds

1,3-Bis(benzimidazolyl)­benzene (Ganta & Chand, 2015 ▸) and 3,3′-dimethyl-1,1′-(1,3-phenylene)bis(1*H*-1,3-ben­zo­di­az­ol-3-ium) bis(tri­fluoro­methane­sul­fon­ate), **1** (Liu *et al.*, 2019 ▸), were synthesized according to literature procedures.

### Synthesis of new compounds

#### Precursor 2^S^

A reported procedure was slightly modified for the synthesis of compound **2^S^** (Wonner *et al.*, 2017*a* ▸, 2019*b* ▸). In a flame-dried 100 ml Schlenk flask, 1.00 g of **1** (1.57 mmol, 1.00 eq.) was added and dissolved in 30 ml dry methanol (52.3 m*M*). To the solution were added 1.00 g sulfur (3.92 mmol, 2.50 eq.) and 1.28 g Cs_2_CO_3_ (3.92 mmol, 2.50 eq.). The resulting suspension was stirred for 24 h under reflux. The mixture was filtered over a pad of silica, which was rinsed twice with dichloromethane (DCM). The solvents were removed under reduced pressure and the crude solid was purified by column chromatography with pentane–EtOAc (2:1 *v*/*v*) (*R*_F_ = 0.33). The solvents were removed under reduced pressure and product **2^S^** was obtained as a white solid (m.p. 240 °C). Yield: 0.547 g (1.36 mmol, 87%). ^1^H NMR (300 MHz, chloro­form-*d*): δ (ppm) = 7.82–7.75 (*m*, 4H), 7.48 (*d*, *J* = 7.9 Hz, 2H), 7.36 (*t*, *J* = 7.7 Hz, 4H), 7.29–7.23 (*m*, 2H), 3.92 (*s*, 6H). ^13^C NMR (75 MHz, chloro­form-*d*): δ (ppm) = 170.30, 136.84, 132.73, 132.64, 130.36, 128.19, 127.74, 123.83 (*d*, *J* = 5.5 Hz), 110.59, 109.05, 31.48. ATR-IR: 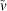 (cm^−1^) = 3030 (*w*), 2924 (*m*), 2345 (*w*), 1736 (*m*), 1728 (*m*), 1601 (*s*), 1589 (*m*), 1476 (*s*), 1454 (*m*), 1427 (*s*), 1377 (*vs*), 1337 (*vs*), 1302 (*s*), 1265 (*s*), 1211 (*vs*), 1161 (*m*), 1144 (*m*), 1119 (*s*), 1015 (*m*), 993 (*m*), 924 (*m*), 887 (*w*), 849 (*w*), 806 (*s*), 743 (*vs*), 714 (*vs*), 692 (*vs*), 615 (*s*), 559 (*vs*), 540 (*m*), 471 (*s*), 432 (*m*), 420 (*vs*). EI–MS (70 eV): *m*/*z*(+) = 402.2 [*M*]^+^, 369.2 [*M* − S]^+^.

#### Precursor 2^Se^

A reported procedure was slightly modified for the synthesis of compound **2^Se^** (Wonner *et al.*, 2017*a* ▸, 2019*b* ▸). In a flame-dried 100 ml Schlenk flask, 0.50 g of **1** (0.783 mmol, 1.00 eq.) was added and dissolved in 15 ml dry methanol (52.3 m*M*). To this solution were added 0.155 g selenium (1.96 mmol, 2.50 eq.) and 0.638 g Cs_2_CO_3_ (1.96 mmol, 2.50 eq.). The resulting suspension was stirred for 24 h under reflux. The mixture was filtered over a pad of silica, which was rinsed twice with DCM. The solvents were removed under reduced pressure and the crude solid was purified by column chromatography with pentane–EtOAc (1:1 *v*/*v*) (*R*_F_ = 0.59). The solvents were removed under reduced pressure and product **2^Se^** was obtained as a white solid (m.p. = 266 °C). Yield: 0.234 g (0.471 mmol, 60%). ^1^H NMR (400 MHz, aceto­nitrile-*d*_3_): δ (ppm) = 7.86–7.75 (*m*, 4H), 7.48 (*d*, *J* = 7.9 Hz, 2H), 7.36 (*t*, *J* = 7.7 Hz, 4H), 7.31–7.24 (*m*, 2H), 3.92 (*s*, 6H). ^13^C NMR (75 MHz, chloro­form-*d*): δ (ppm) = 167.36, 137.58, 133.97, 133.67, 130.52, 128.97, 128.97, 124.29 (*d*, *J* = 1.2 Hz), 111.31, 109.58, 33.58. ATR-IR: 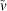 (cm^−1^) = 3053 (*w*), 2922 (*m*), 2853 (*w*), 1726 (*w*), 1601 (*m*), 1589 (*m*), 1493 (*m*), 1474 (*s*), 1456 (*m*), 1425 (*m*), 1375 (*s*), 1333 (*vs*), 1306 (*s*), 1298 (*s*), 1263.37 (*s*), 1211 (*s*), 1161 (*m*), 1144 (*m*), 1121 (*s*), 1084 (*s*), 1015 (*m*), 991 (*m*), 874 (*m*), 853 (*m*), 808 (*s*), 802 (*s*), 745 (*vs*), 709 (*s*), 691 (*s*), 675 (*m*), 646 (*m*), 617 (*m*), 592 (*m*), 557 (*s*), 534 (*m*), 469 (*m*), 430 (*m*). EI–MS (70 ev): *m*/*z* = 417.10 [*M* − Se]^+^.

#### Chalcogen-bonding donor 3^S^

A reported procedure was slightly modified for the synthesis of compound **3^S^** (Wonner *et al.*, 2017*a* ▸, 2019*b* ▸). To a flame-dried 50 ml Schlenk flask was added 0.400 g of **2^S^** (0.984 mmol, 1.00 eq.) dissolved in 15 ml dry DCM (66.3 m*M*). Afterwards, 0.281 ml methyl tri­fluoro­methane­sulfonate (0.408 g, 2.48 mmol, 2.50 eq.) was added dropwise. The mixture was stirred for 24 h at room tem­per­ature. The solvent was removed under reduced pressure. The crude solid was washed three times with di­ethyl ether and three times with pentane, and then dried under high vacuum. Product **3^S^** was obtained as a pale-yellow solid (m.p. = 222 °C). Yield: 0.630 g (0.858 mmol, 86%). ^1^H NMR (300 MHz, acetonitrile-*d*_3_): δ (ppm) = 8.14 (*dd*, *J* = 9.0, 6.9 Hz, 1H), 8.07–8.01 (*m*, 2H), 7.99–7.94 (*m*, 3H), 7.75 (*qd*, *J* = 8.2 Hz, *J* = 1.2 Hz, 4H), 7.58 (*d*, *J* = 7.8 Hz, 2H), 4.22 (*s*, 6H), 2.51 (*s*, 6H). ^13^C NMR (75 MHz, acetonitrile-*d*_3_): δ (ppm) = 151.97, 135.33, 134.21, 133.95, 131.59, 129.21, 128.85, 127.87, 114.35, 113.82, 34.55, 18.20. ^19^F NMR (235 MHz, aceto­nitrile-*d*_3_): δ (ppm) = −79.31 (*s*, 6F). ATR-IR: 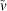 (cm^−1^) = 3066 (*w*), 3045 (*w*), 1604 (*w*), 1508 (*m*), 1498 (*m*), 1483 (*m*), 1463 (*m*), 1398 (*w*), 1319 (*w*), 1254 (*vs*), 1223 (*vs*), 1148 (*vs*), 1088 (*m*), 1028 (*vs*), 982 (*m*), 925 (*w*), 849 (*w*), 818 (*m*), 799 (*w*), 767 (*s*), 754 (*s*), 746 (*s*), 698 (*s*), 633 (*vs*), 573 (*s*), 559 (*s*), 515 (*vs*), 444 (*m*), 417 (*m*). ESI–MS: *m*/*z*(+) = calc. 581.10 [*M* + OTf]^+^; found 581.10 [*M* + OTf]^+^. *m*/*z*(−) = calc. 148.95 [OTf]^−^; found 149.29 [OTf]^−^.

#### Chalcogen-bonding donor 3^Se^

A reported procedure was slightly modified for the synthesis of compound **3^Se^** (Wonner *et al.*, 2017*a* ▸, 2019*b* ▸). To a flame-dried 50 ml Schlenk flask was added 0.150 g of **2^Se^** (0.302 mmol, 1.00 eq.) dissolved in 20 ml dry DCM (15.1 m*M*). Afterwards, 85.5 µl methyl tri­fluoro­methane­sulfonate (0.124 g, 0.756 mmol, 2.50 eq.) was added dropwise. The mixture was stirred for 24 h at room tem­per­ature. The solvent was removed under reduced pressure. The crude solid was washed three times with di­ethyl ether and three times with pentane, and then dried under high vacuum. The crude solid was dissolved in the minimum amount of aceto­nitrile and precipitated by the addition of di­ethyl ether. The solid was filtered off and dried under high vacuum. Product **3^Se^** was obtained as a pale-yellow solid (m.p. = 239 °C) after washing with di­ethyl ether and pentane. Yield: 0.230 g (0.278 mmol, 92%). ^1^H NMR (400 MHz, aceto­nitrile-*d*_3_): δ (ppm) = 8.14 (*dd*, *J* = 8.8, 6.7 Hz, 1H), 8.07–8.01 (*m*, 2H), 7.99–7.94 (*m*, 3H), 7.82–7.68 (*m*, 4H), 7.58 (*d*, *J* = 7.9 Hz, 2H), 4.22 (*s*, 6H), 2.50 (*s*, 6H). ^13^C NMR (75 MHz, aceto­nitrile-*d*_3_): δ (ppm) = 148.30, 136.11, 134.90, 134.21, 133.71, 128.93, 128.59, 128.28, 114.33, 113.87, 35.73, 11.10. ^19^F NMR (377 MHz, chloro­form-*d*): δ (ppm) = −79.34 (*s*, 6F). ATR-IR: 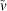 (cm^−1^) = 3082 (*w*), 2951 (*w*), 2360 (*w*), 1740 (*w*), 1605 (*w*), 1506 (*m*), 1476 (*m*), 1458 (*m*), 1404 (*w*), 1360 (*w*), 1308 (*w*), 1254 (*vs*), 1221 (*s*), 1138 (*s*), 1092 (*w*), 1078 (*w*), 1026 (*vs*), 935 (*w*), 908 (*w*), 829 (*w*), 773 (*w*), 758 (*s*), 739 (*m*), 710 (*w*), 692 (*m*), 667.37 (*w*), 633 (*vs*), 571 (*m*), 557 (*m*), 515 (*s*), 469 (*w*), 449 (*w*), 430 (*w*). ESI–MS: *m*/*z*(+) = calc. 676.99 [*M* − OTf]^+^, 264.02 [*M* − 2OTf]^2+^; found 676.64 [*M* − OTf]^+^, 263.00 [*M* − 2OTf]^2+^. *m*/*z*(−) = calc. 148.95 [OTf]^−^; found 148.64 [OTf]^−^.

### Refinement

Crystal data, data collection and structure refinement details are summarized in Table 1 ▸. All H atoms were refined using the riding model in idealized positions and isotropic radii, with C—H distances of 0.98 Å and *U*_iso_(H) = 1.5*U*_eq_(C) for methyl H atoms, and C—H = 0.95 Å and *U*_iso_(H) = 1.2*U*_eq_(C) for other H atoms.

## Discussion

As described in the *Introduction*, selenium-based chalcogen-bonding catalysts deriving from a 1,3-bis­(benzimidazoliumyl)benzene frame were applied in several reactions. Whereas the first report describes the application in a stoichiometric activation of a carbon–bromine bond (Wonner *et al.*, 2017*a* ▸), the following article dealt with the catalytic activation of a carbon–chloride bond (Wonner *et al.*, 2017*b* ▸). As these reactions rely at least partially on the binding of the catalyst to the halide leaving group, they are typically easier to activate or catalyse than reactions involving neutral organic functional groups like carbonyls or imines. Therefore, the next step was the activation of quinolines in a transfer hydrogenation reaction (Wonner *et al.*, 2019*b* ▸). However, this benchmark reaction showed surprisingly similar catalytic activities between preorganized and non-preorganized catalysts, as well as between sulfur- and selenium-based chalcogen-bonding donors. On theoretical grounds, the preorganized and the selenium-based variant would have been expected to be noticeably more potent. In particular, the comparable per­for­mance of the catalyst differing in the chalcogen atom was puzzling and intriguing, as this could mean that better accessible sulfur variants could be used in catalysis without much loss of turn-over frequency.

**Figure 1:**
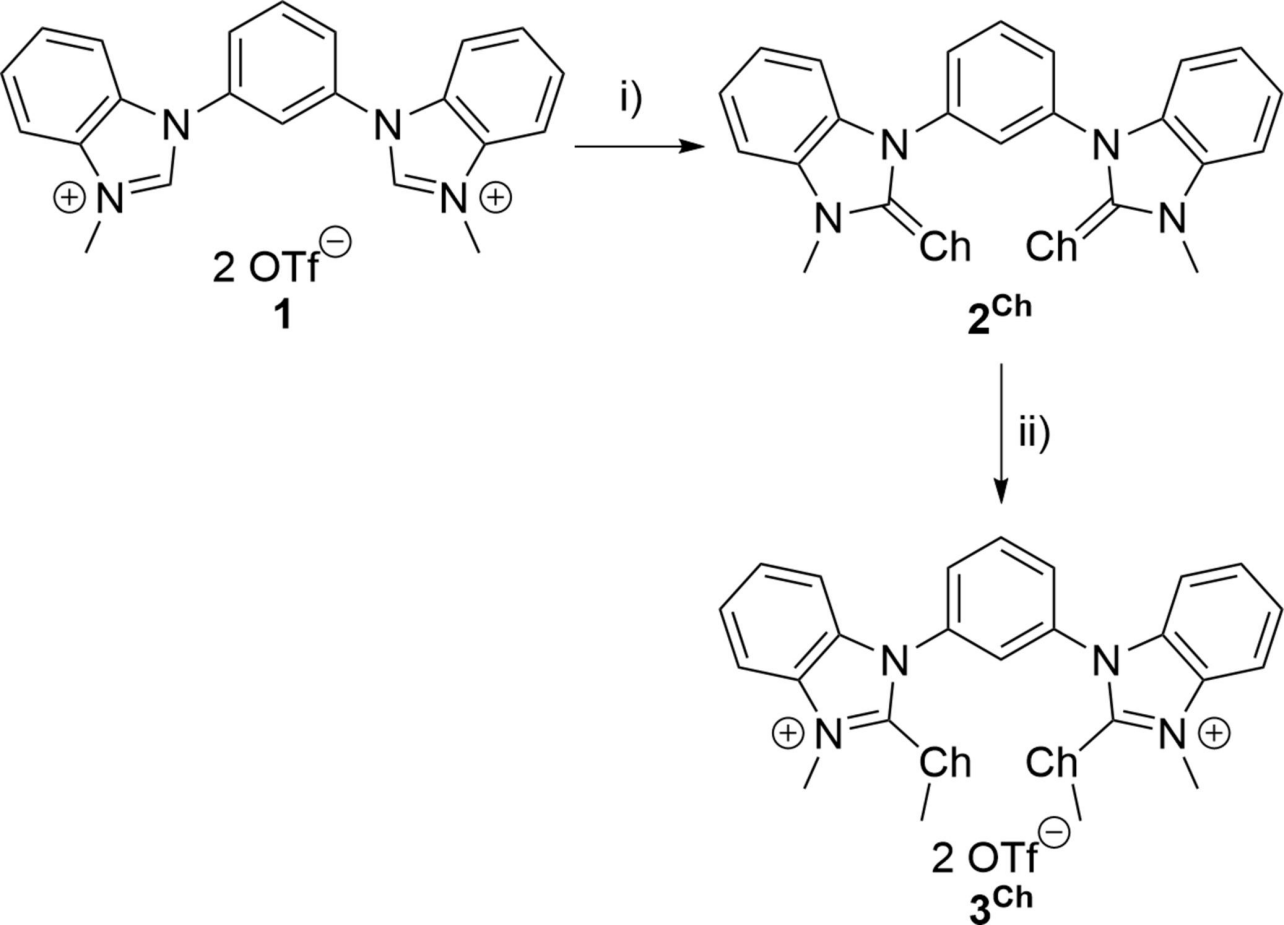
Synthesis of the chalcogen-bonding catalysts investigated in this report. (i) Ch (2.50 eq.), Cs_2_CO_3_ (2.50 eq.), MeOH, reflux, 3 d (**2^S^** = 87%; **2^Se^** = 60%); (ii) MeOTf (2.50 eq.), DCM, room temperature, 24 h (**3^S^** = 86%; **3^Se^** = 92%) (Ch = S or Se) (Wonner *et al.*, 2019*b* ▸).

To further investigate this issue, the non-preorganized sulfur- and selenium-based catalysts were subjected to crystallization studies to investigate the chalcogen-bonding properties. Crystallization of these compounds proved difficult despite various efforts made while varying parameters that affect the crystallization process, such as tem­per­ature, concentration and crystallization techniques. The octyl chains, which are responsible for the good solubility of the catalysts in organic solvents, are most likely the reason for this. To avoid this challenge, the respective all-methyl­ated catalysts (**3^S^** and **3^Se^**) were synthesized. The preparation of these compounds followed an already reported route (Fig. 1 ▸) (Wonner *et al.*, 2019*b* ▸), with 1,3-bis­(benzimidazolyl)­benzene (Ganta & Chand, 2015 ▸) and compound **1** (Liu *et al.*, 2019 ▸) being synthesized according to literature procedures. The next step was the formation of the respective sulfur or selenium urea compounds (**2^S^** and **2^Se^**) under basic conditions. The final step, methyl­ation of the urea compound, yielded the all-methyl­ated compounds **3^S^** and **3^Se^**.

**Figure 2:**
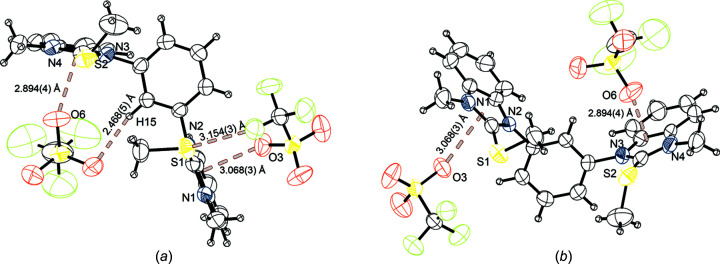
The molecular structure of **3^S^** with the chalcogen bonding, anion–π interactions and hydrogen bonding marked, and with displacement ellipsoids drawn at the 50% probability level.

Next, crystallization studies were carried out. The vapour diffusion method was applied successfully to crystallize com­pound **3^S^** from di­chloro­methane and cyclo­hexane (Fig. 2 ▸). This compound crystallizes in the monoclinic space group *P*2_1_/*c*. The unit-cell constants are *a* = 7.7776 (2), *b* = 22.5823 (6) and *c* = 18.2338 (5) Å, with a volume of 3160.16 (15) Å^3^ and a density of 1.536 Mg m^−3^. The unit cell is built up of four ion pairs. Within this structure, an intermolecular interaction between atoms S1 and F1 of a tri­fluoro­methane­sul­fon­ate anion is observed. The S1⋯F1 distance is 3.154 (3) Å, 96% of the van der Waals radii (Σ*r*_ω_), and the directionality is rather poor (C1—S1⋯F1 = 146.9°). The supposedly more Lewis basic O atoms of the tri­fluoro­methane­sul­fon­ate anion are involved in two anion–π interactions with the imidazole unit of benzimidazole. These interactions have distances O6⋯cent = 2.894 (4) Å and O3⋯cent = 3.068 (3) Å from the calculated centroid (cent) of the imidazole unit to the tri­fluoro­methane­sul­fon­ate O atoms. From this O atom to the closest C atom, the distances in both cases are 11% shorter than the Σ*r*_ω_ (which possibly indicates a stronger interaction compared to the already mentioned chalcogen-bonding interaction). In addition, hydrogen bonding between atoms O4 and H15 in the 2-position of the central arene core [with H15⋯O4 = 2.468 (5) Å, 94% of Σ*r*_ω_, and C15—H15⋯O4 = 166.5°] is observed.

Overall, merely a chalcogen-bonding interaction between sulfur and fluorine was observed with a distance that corresponds to 96% of the Σ*r*_ω_. Additional weak hydrogen bonding found in this structure between the backbone H atoms of the arene core and the benzimidazolium unit will not be further discussed in this article.

**Figure 3:**
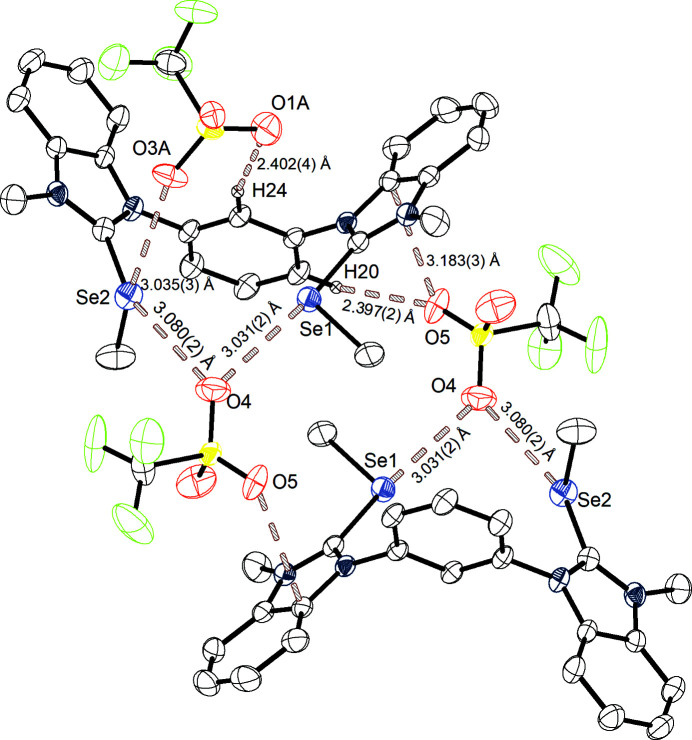
Structure A of **3^Se^** with the chalcogen bonding, hydrogen bonding and anion–π interactions marked, and with displacement ellipsoids drawn at the 50% probability level. For the sake of clarity, only H atoms involved in hydrogen bonding are shown. Symmetry operation to generate the second molecule of **3^Se^** and tri­fluoro­methane­sul­fon­ate: −*x* + 1, −*y* + 1, −*x* + 1.

Under the same crystallization conditions, the selenium-con­taining equivalent **3^Se^** crystallizes also in the monoclinic space group *P*2_1_/*c* (Fig. 3 ▸). The unit-cell constants are *a* = 7.74775 (4), *b* = 13.12863 (8) and *c* = 31.27496 (18) Å, with a volume of 3181.02 (3) Å^3^ and a density of 1.722 Mg m^−3^. The unit cell con­tains four ion pairs and bidentate chalcogen bonding from the Se1 and Se2 centres of **3^Se^** to tri­fluoro­methane­sul­fon­ate atom O4 is observed. Some of the anions involved in these interactions are disordered over two positions. The interaction distances are Se1⋯O4 = 3.031 (2) Å and Se2⋯O4 = 3.080 (2) Å, with angles of C3—Se1⋯O4 = 165.31° and C11—Se2⋯O4 = 159.89°. The sum of the van der Waals radii is 3.42 Å and therefore the observed distances amount to 89 and 90% of this measure, thus classifying this interaction as stronger chalcogen bonding compared to the interaction found in the structure of **3^S^**. Atom Se2, which already features chalcogen bonding in the elongation of the benzimidazole C11—Se bond, faces also a second chalcogen-bonding interaction to atom O3*A* of the second tri­fluoro­methane­sul­fon­ate anion in the elongation of the CH_3_—Se bond, with an Se2⋯O3*A* distance of 3.035 (3) Å and a C2—Se2⋯O3*A* angle of 158.07° (89% of Σ*r*_ω_). This additional interaction vividly demonstrates the property of chalcogens to form *two* noncovalent interactions based on their Lewis acidity. Although only one electron-withdrawing substituent is used, both axes simultaneously seem to be available for chalcogen bonding.

Taking a closer look at the unit cell, several other intermolecular interactions are found. The remaining O atoms (O5 and O6) of this tri­fluoro­methane­sul­fon­ate anion are involved in two anion–π interactions to an imidazole group of benz­im­id­azole [O5⋯cent = 3.269 (3) Å and O6⋯cent = 3.307 (3) Å]. These interactions can be considered very weak, as the distances are close to the actual Σ*r*_ω_ with 99 and 100%. Atom O5 faces additional hydrogen bonding, formed with atom H20 sitting in the 4-position of the central arene core [H20⋯O5 = 2.397 (2) Å, 91% of Σ*r*_ω_, and C20—H20⋯O5 = 143.01°]. Moreover, atom O1*A* of the tri­fluoro­methane­sul­fon­ate anion is employed in a second hydrogen-bonding interaction to H24, sitting in the 2-position of the central arene core [H24⋯O1*A* = 2.402 (4) Å, 92% of Σ*r*_ω_, and C24—H24⋯O1*A* = 161.21°]. These two hydrogen-bonding interactions indicate a Lewis acidic character of the benzene H atoms. The overall picture of the unit cell is therefore described by two chalcogen-bonding donors which are bridged with two tri­fluoro­methane­sul­fon­ate anions *via* chalcogen bonding, hydrogen bonding and anion–π interactions. This structure features comparable binding distances within the chalcogen-bonding donor, *e.g.* the distances of the carbon–selenium bonds and also similar distances of the observed intermolecular chalcogen bonding to a related compound (Wonner *et al.*, 2017*a* ▸). The distances of the anion–π interactions in **3^S^** are on average 9% shorter than the corresponding interactions found in this crystal structure. This could be explained by the fact that one tri­fluoro­methane­sul­fon­ate O atom forms this interaction in the structure of **3^S^**, whereas two tri­fluoro­methane­sul­fon­ate O atoms are involved in the interaction with **3^Se^**, which geometrically leads to an increased interaction distance. Another explanation could be the more electron-withdrawing properties of sulfur compared to selenium based on the difference in their electronegativity, leading to a more polarized imidazolium system when sulfur is implemented.

**Figure 4:**
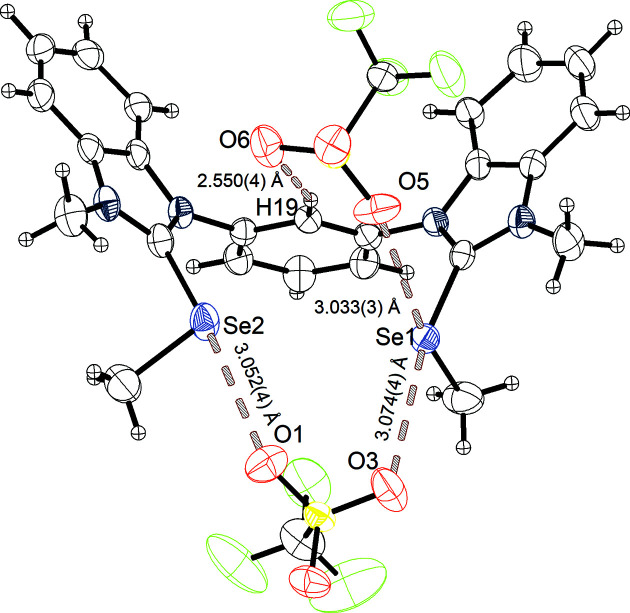
Structure B of **3^Se^** grown at 4–5 °C, with the chalcogen bonding and hydrogen bonding marked, and with displacement ellipsoids drawn at the 50% probability level.

Besides this structure, a second crystal structure (B) of **3^Se^** was obtained from crystallization at lower tem­per­atures (4–5 °C) (Fig. 4 ▸). The crystal system is again monoclinic with the space group *P*2_1_/*c*. The parameters of the unit cell are *a* = 13.8634 (4), *b* = 7.7558 (2) and *c* = 29.3267 (7) Å, with a volume of 3144.59 (14) Å^3^ and a density of 1.742 Mg m^−3^. The unit cell once more consists of four ion pairs. Different to the previously described solid-state structure (A of **3^Se^**), each selenium centre features its own chalcogen-bonding interaction to an O atom of a tri­fluoro­methane­sul­fon­ate anion. The distances of these interactions are Se1⋯O3 = 3.074 (4) Å (90% of Σ*r*_ω_) and Se2⋯O1 = 3.052 (4) Å (89% of Σ*r*_ω_), with C3—Se1⋯O3 = 167.87° and C5—Se2⋯O1 = 171.26°. Besides these interactions and differences, yet another short contact is observed. Atom Se1, already featuring chalcogen bonding in the elongation of the benzimidazole C3—Se1 bond, faces also a second chalcogen-bonding interaction to atom O5 of the second tri­fluoro­methane­sul­fon­ate anion in the elongation of the CH_3_—Se bond. This interaction [Se1⋯O5 = 3.033 (3) Å, 89% of Σ*r*_ω_, with C3—Se1⋯O5 = 161.19°] gives Se1 again a biaxial character. However, only one of the two Se atoms features this biaxial system, as also seen in structure A of **3^Se^** (Fig. 3 ▸). Between the two crystal structures of **3^Se^**, only minor differences are observed, as the Se⋯O chalcogen-bonding interaction distances are in both cases approximately 90% of the van der Waals radii. The angles of the structure obtained at lower tem­per­atures are closer to 170°. Both structures feature a biaxial chalcogen centre with similar Se⋯O distances (89% of the van der Waals radii) and similar angles below 160°, which are less directional than the interactions in the elongation of the benzimidazole C—Se bond. Similar to the previously described structures, the 2-position hydrogen (H19) of the central arene core shows its Lewis acidic po­tential in a hydrogen-bonding interaction to one of the tri­fluoro­methane­sul­fon­ate anions (O6) [H19⋯O6 = 2.550 (4) Å, 98% of Σ*r*_ω_, and C19—H19⋯O6 = 157.75°].

**Figure 5:**
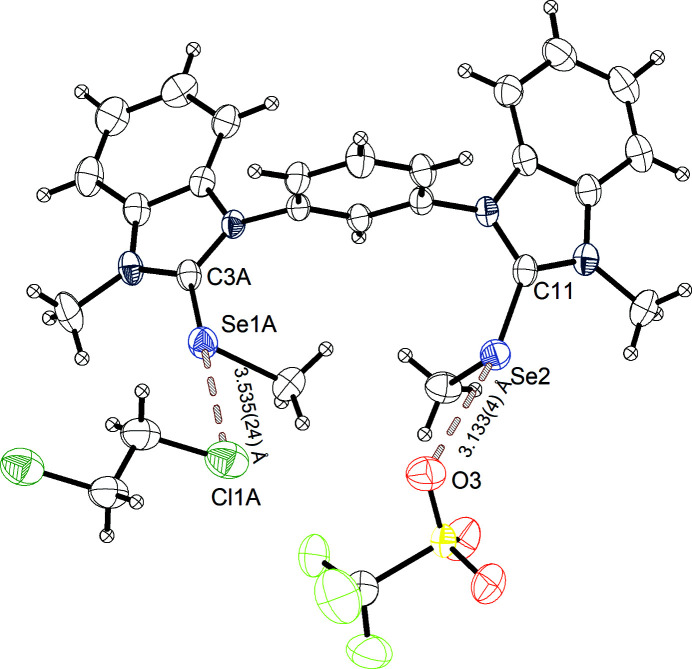
Structure C of **3^Se^**, with chalcogen bonding to tri­fluoro­methane­sul­fon­ate and 1,2-di­chloro­ethane marked, and with displacement ellipsoids drawn at the 50% probability level. The di­chloro­ethane molecule and tri­fluoro­methane­sul­fon­ate anion are generated by (*x* + 1, *y*, *z*).

Changing the solvent in the crystallization experiments from di­chloro­methane to 1,2-di­chloro­ethane ended up yielding yet another structural variation (structure C of **3^Se^**) (Fig. 5 ▸). In this case, a triclinic space group *P* 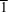 , with unit-cell parameters of *a* = 9.3672 (1), *b* = 13.3265 (2) and *c* = 13.6445 (1) Å, with a volume of 1647.49 (3) Å^3^ and a density of 1.762 Mg m^−3^ was obtained. The unit cell is built up of two ion pairs along with four crystallized 1,2-di­chloro­methane molecules. The binding distances and angles within the donor are comparable to the previously described structure of **3^Se^**. The crystal structure features two independent intermolecular interactions. The first one includes the Se1 centre and atom Cl1 of 1,2-di­chloro­ethane, with an Se1⋯Cl1 distance of 3.535 (24) Å, which corresponds to 97% of the Σ*r*_ω_, and a C3*A*—Se1*A*⋯Cl1*A* angle of 176°. The second short contact can be found between the Se2 centre and tri­fluoro­methane­sul­fon­ate atom O3, with an Se2⋯O3 distance of 3.133 (4) Å (92% of Σ*r*_ω_) and a C11—Se2⋯O3 angle of 168°. This Se⋯O interaction is again comparable to the other observed chalcogen-bonding interactions for **3^Se^**. The distance of Se1*A* to the Cl1*A* atom of 1,2-di­chloro­ethane is very close to the Σ*r*_ω_, which indicates a weaker interaction compared to the interaction of selenium with a tri­fluoro­methane­sul­fon­ate O atom. However, since the solvent is not charged and thus a neutral chalcogen-bonding acceptor is involved in the Se⋯Cl interaction, a weaker interaction is expected. The slightly longer distance to the tri­fluoro­methane­sul­fon­ate O atom compared to the distance in structures A and B of **3^Se^** can be explained by the fact that two independent Se⋯O/Cl chalcogen-bonding interactions, yet no co-operative effect, are present.

**Figure 6:**
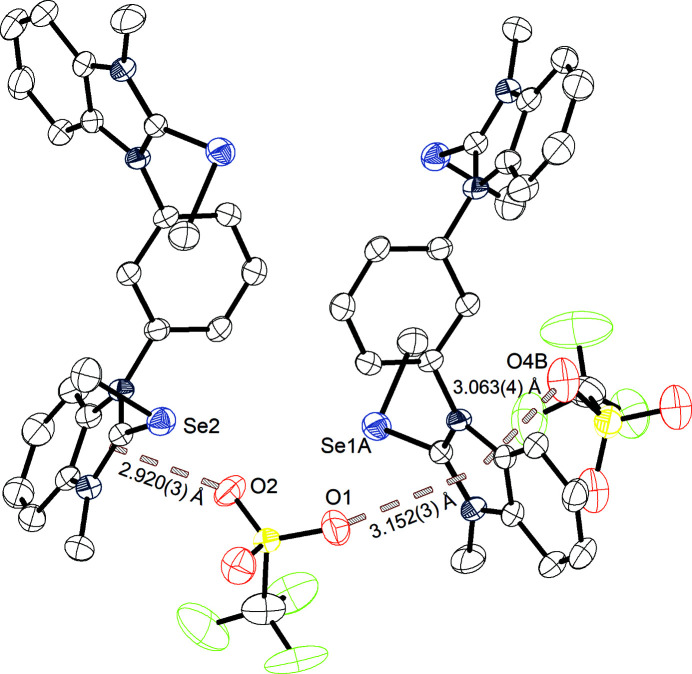
Structure C of **3^Se^**, with the anion–π interactions to tri­fluoro­methane­sul­fon­ate marked, and with displacement ellipsoids drawn at the 50% probability level. H atoms and 1,2-di­chloro­ethane molecules have been omitted for clarity. The first tri­fluoro­methane­sul­fon­ate anion is generated by (*x*, *y*, *z* − 1) and the second tri­fluoro­methane­sul­fon­ate anion and **3^Se^** are generated by (−*x* + 1, −*y* + 1, −*z*).

In addition, this structure also features anion–π interactions (Fig. 6 ▸). An imidazole moiety of one of the two benzimidazolium systems coordinates with each side to one tri­fluoro­methane­sul­fon­ate anion [O1⋯cent = 3.152 (3) Å and O4*B*⋯cent = 3.063 (4) Å]. Atom O2 sitting on the same tri­fluoro­methane­sul­fon­ate anion as O1 also forms an anion–π interaction [O2⋯cent = 2.920 (3) Å], becoming therefore a bridging moiety between two chalcogen-bonding donors. The imidazole system of this interaction does not feature a second anion–π interaction. The distances of these interactions are 5–13% shorter than the Σ*r*_ω_ of the O atom to the closest C atom. These anion–π interactions are up to 10% shorter compared to those found in structure A of **3^Se^** (Fig. 4 ▸), and provide further indications that the weak anion–π interaction in structure A is due to geometric limitations. Comparing the interaction with **3^S^** indicates interactions of similar strength, even though selenium is less electronegative and does not polarize the imidazole moiety of benzimidazole in the same fashion as the more electronegative sulfur could do.

Considering the fact that the carbon–nitro­gen bonds to the central scaffold are free to rotate and therefore the Lewis acidic selenium centres could in principle point in opposite directions, it is interesting to see that a *syn*-like conformer has been observed in all of the presented structures. The only exception is the disorder found in structure C of **3^Se^**, with a ratio of 95:5 of the *syn*- and *anti*-like conformers. This is especially remarkable for the structure of **3^S^** and structure C of **3^Se^**, as no bidentate interaction of the Lewis acidic centres keeps the compound in this conformation, and the last structure of **3^Se^**, as two independent chalcogen-bonding interactions are found, which in principle could also point in different directions.

**Figure 7:**
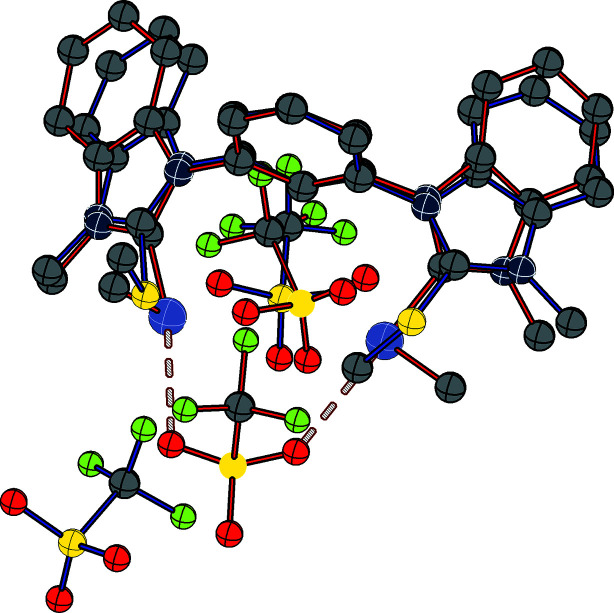
Overlay plot of the structures of **3^S^** (blue bonds) and **3^Se^** (structure B) (red bonds).

Overlaying the structure of **3^S^** and structure B of **3^Se^** stresses the similarity of the compounds (Fig. 7 ▸). The 1,3-bis­benzimidazolium scaffolds are matching each other in terms of geometry. The biggest difference is that one of the methyl groups located at the chalcogens (right side in this plot) is pointing in opposite direction. Nevertheless, they are roughly occupying the same space. The bigger difference is the orientation of one of the two tri­fluoro­methane­sul­fon­ate anions. Whereas for **3^S^**, the CF_3_ group of tri­fluoro­methane­sul­fon­ate is facing the sulfur, it is the SO_3_ moiety that is facing the selenium centre in structure B of **3^Se^**.

Besides these solid-state structures, we also performed density functional theory (DFT) calculations to further evaluate and elucidate chalcogen-bonding properties of the herein considered catalyst system. For the *in silico* investigations, the M06-2X functional (Zhao & Truhlar, 2008 ▸) with the triple-zeta def2-TZVP basis set (Weigend & Ahlrichs, 2005 ▸) and Grimme’s D3 dispersion correction was applied (Grimme *et al.*, 2010 ▸; Grimme, 2012 ▸). In the electrostatic plots, the energy potentials are set to 87.8 and 153.1 kcal mol^−1^ and are projected with 0.001 e Bohr^−3^. The surface map values are summed up in Table 2 ▸ in kcal mol^−1^.

**Figure 8:**
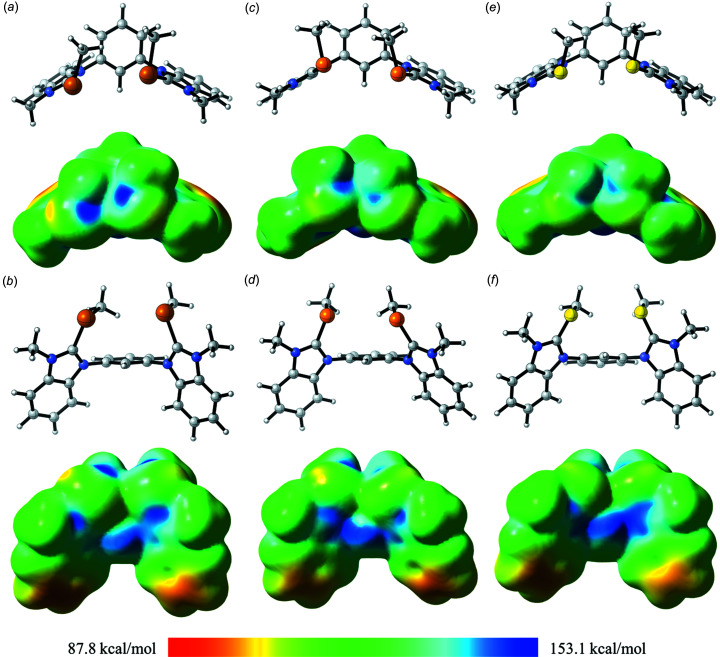
Plots of the optimized structure of **3^Ch^** created with *CLYView* (Legault, 2009 ▸), along with the electrostatic potential surface with an energy scale of 87.8–153.1 kcal mol^−1^ projected with 0.001 e Bohr^−3^. (*a*)/(*b*) Ch = Te, (*c*)/(*d*) Ch = Se and (*e*)/(*f*) Ch = S.

First, we consider the ‘depth’ of the σ-holes, which are localized at the elongation of the benzimidazole C—Ch bonds [Figs. 8 ▸(*a*), 8(*c*) and 8(*e*)]. According to the expected tendency, the surface map values for these σ-holes (at Ch1 and Ch2) are decreasing with the decreasing atomic number of the implemented chalcogen (Table 2 ▸). In any case, both σ-holes in the described areas show slightly different values (*e.g.* 152.4 and 153.3 kcal mol^−1^ for **3^Te^**). The comparison of **3^Se^** and **3^S^** is of special interest, as these data can be compared with the previously reported catalysis data (Wonner *et al.*, 2019*b* ▸). If a deeper σ-hole depth is equal to stronger catalytic activity, **3^Se^** should show an increased activation compared to **3^S^**. As stated above, this is not the case. Therefore, the observed catalytic activity in the reduction of quinolines cannot be further explained taking only these σ-holes into account.

Shining light on the remaining σ-holes of these systems, that in the elongation of the CH_3_—Ch bond [Figs. 8 ▸(*b*), 8(*d*) and 8(*f*)] could provide hints on the origins of the comparable catalytic properties. **3^Te^** shows defined σ-holes [Fig. 8 ▸(*b*)] in this region with decreased absolute values of potentials [with respect to those of the C(benzim)—Ch bond] of 147.8 and 147.2 kcal mol^−1^. Besides this electron-poor area, the H atom in the 2-position of the central arene core also features an electropositive potential of 145.2 kcal mol^−1^.

Moving to **3^Se^**, these σ-holes are less defined and less separated from the electron-poor region at the central arene core [Fig. 8 ▸(*d*)]. In contrast to **3^Te^**, the four chalcogen-based electron-deficient areas for **3^Se^** show comparable energies (Table 2 ▸, entry 2). The estimated energy for the σ-hole of the 2-position H atom shows a potentially more Lewis acidic area compared to those located at the chalcogen. In two of the three crystal structures of **3^Se^**, we observed weak hydrogen bonding of this H atom. The overall maximum value for **3^Se^** was observed right between the less defined chalcogen and hydrogen σ-hole, with a value of 147.9 kcal mol^−1^. For **3^Te^**, no such value was found, as they did not overcome the classic σ-hole values.

Continuing these investigations with **3^S^**, no separation of the σ-holes (CH_3_—S bond) is spotted and a connected electron-deficient system covering these σ-holes and the 2-position H atom of the central core is observed. The surface map values (Table 2 ▸, entry 3) are reversed to those from **3^Te^**, as the 2-position seems to be the most Lewis acidic centre, followed by the CH_3_—S bond σ-holes. The C(benzim)—S bond σ-holes are those that are the least Lewis acidic. Matching this with the crystal structure of **3^S^**, these data are confirmed, as no chalcogen bonding in the elongation of the C(benzim)—S bond is found, but one intermolecular action is observed in the elongation of the CH_3_—S bond which is theoretically favoured. Like **3^Se^**, the most electron-deficient area of **3^S^** is again observed between the central arene core and the chalcogen. However, in the case of **3^S^**, the difference to the second most Lewis acidic centre (2.5 kcal mol^−1^) is larger than the difference in **3^Se^** (1.0 kcal mol^−1^). Since sulfur is more electron withdrawing, the larger electron deficient area might be explained by the more electron-withdrawing sulfur, which impacts the whole structure. This might also explain the reason for the catalytic activity in the reduction of quinolones. Nevertheless, it is necessary to mention that various reference experiments were carried out in that reaction which indicate an activation based on chalcogen bonding, for instance, by using the respective hydrogen-bonding equivalents.

## Conclusion

In summary, we presented four solid-state structures of sulfur- and selenium-con­taining chalcogen-bonding catalysts. The structures and their interactions are matched with *in silico*-calculated respective structures to shine some light on the fairly surprising results of their application in the activation of quinolines. The structure of **3^S^** features one chalcogen-bon­ding interaction from sulfur to fluorine of the tri­fluoro­methane­sul­fon­ate counter-anion, which has an interaction distance close to the sum of the van der Waals radii, whilst also anion–π interactions and hydrogen bonding are observed. These results are in line with the theoretically predicted data. In three crystal structures of the selenium-based analogue **3^Se^**, different mono- and bidentate, as well as biaxial binding motifs to neutral and negatively charged molecules based on chalcogen bonding, are present. Nevertheless, these structures also con­tained anion–π interactions along with hydrogen bonding. The results could also be related to calculational data. We further extended the DFT calculations to the appropriate tellurium-based compound, which could not be synthesized, and observed the most suitable theoretical properties for chalcogen bonding. However, the unusual activities of the previous report on the reduction of quinolines could not be satisfactorily explained.

## Supplementary Material

Crystal structure: contains datablock(s) ts-049_3S, TS-056_3Se-C, TS-056_twin_twin1_hklf4_3Se-B, TS-058_3Se_A, global. DOI: 10.1107/S2053229622011536/qw3003sup1.cif

Structure factors: contains datablock(s) ts-049_3S. DOI: 10.1107/S2053229622011536/qw3003ts-049_3Ssup2.hkl

Structure factors: contains datablock(s) TS-056_3Se-C. DOI: 10.1107/S2053229622011536/qw3003TS-056_3Se-Csup3.hkl

Structure factors: contains datablock(s) TS-056_twin_twin1_hklf4_3Se-B. DOI: 10.1107/S2053229622011536/qw3003TS-056_twin_twin1_hklf4_3Se-Bsup4.hkl

Structure factors: contains datablock(s) TS-058_3Se-A. DOI: 10.1107/S2053229622011536/qw3003TS-058_3Se-Asup5.hkl

Energies of computed structures. DOI: 10.1107/S2053229622011536/qw3003sup6.txt

Coordinates of computed structures. DOI: 10.1107/S2053229622011536/qw3003sup7.txt

Additional tables, figures and spectra. DOI: 10.1107/S2053229622011536/qw3003sup8.pdf

CCDC references: 2201758, 2201759, 2201760, 2201761

## Figures and Tables

**Table 1:** Experimental details Experiments were carried out at 170 K with Cu *K*α radiation using a Rigaku XtaLAB Synergy Dualflex diffractometer with a HyPix detector. H-atom parameters were constrained.

	**3^S^**	**3^Se^-A**	**3^Se^-B**	**3^Se^-C**
Crystal data				
Chemical formula	C_24_H_24_N_4_S_2_^2+^·2CF_3_O_3_S^−^	C_24_H_24_N_4_Se_2_^2+^·2CF_3_O_3_S^−^	C_24_H_24_N_4_Se_2_^2+^·2CF_3_O_3_S^−^	C_24_H_24_N_4_Se_2_^2+^·2CF_3_O_3_S^−^·0.5C_2_H_4_Cl_2_
*M*_r_	730.73	824.53	824.53	874.01
Crystal system, space group	Monoclinic, *P*2_1_/*c*	Monoclinic, *P*2_1_/*c*	Monoclinic, *P*2_1_/*c*	Triclinic, *P* 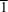
*a*, *b*, *c* (Å)	7.7776 (2), 22.5823 (6), 18.2338 (5)	7.74775 (4), 13.12863 (8), 31.27496 (18)	13.8634 (4), 7.7558 (2), 29.3266 (7)	9.3672 (1), 13.3265 (2), 13.6445 (1)
α, β, γ (°)	90, 99.329 (2), 90	90, 90.6141 (5), 90	90, 94.246 (2), 90	102.515 (1), 93.231 (1), 96.198 (1)
*V* (Å^3^)	3160.16 (15)	3181.02 (3)	3144.59 (14)	1647.49 (3)
*Z*	4	4	4	2
μ (mm^−1^)	3.51	4.88	4.94	5.48
Crystal size (mm)	0.13 × 0.06 × 0.03	0.29 × 0.14 × 0.08	0.03 × 0.02 × 0.01	0.19 × 0.10 × 0.08

Data collection				
Absorption correction	Multi-scan (*CrysAlis PRO*; Rigaku OD, 2018 ▸)	Gaussian (*CrysAlis PRO*; Rigaku OD, 2018 ▸)	Gaussian (*CrysAlis PRO*; Rigaku OD, 2018 ▸)	Gaussian (*CrysAlis PRO*; Rigaku OD, 2018 ▸)
*T*_min_, *T*_max_	0.579, 1.000	0.300, 1.000	0.900, 0.969	0.456, 1.000
No. of measured, independent and observed [*I* > 2σ(*I*)] reflections	21606, 5564, 4631	37979, 5613, 5268	28154, 5529, 4865	19605, 5795, 5307
*R*_int_	0.043	0.039	0.073	0.046
(sin θ/λ)_max_ (Å^−1^)	0.595	0.595	0.595	0.595

Refinement				
*R*[*F* ^2^ > 2σ(*F* ^2^)], *wR*(*F* ^2^), *S*	0.073, 0.205, 1.09	0.028, 0.070, 1.02	0.053, 0.142, 1.06	0.048, 0.127, 1.05
No. of reflections	5564	5613	5529	5795
No. of parameters	419	486	419	484
No. of restraints	0	0	0	28
Δρ_max_, Δρ_min_ (e Å^−3^)	0.69, −0.39	0.60, −0.78	1.21, −0.84	0.84, −0.80

Computer programs: *CrysAlis PRO* (Rigaku OD, 2018 ▸), *SHELXT* (Sheldrick, 2015*a* ▸), *shelXle* (Hübschle *et al.*, 2011 ▸), *SHELXL2018* (Sheldrick, 2015*b* ▸), *DIAMOND* (Putz & Brandenburg, 2014 ▸), *Mercury* (Macrae *et al.*, 2020 ▸) and *WinGX* (Farrugia, 2012 ▸).

**Table 2:** Surface map values for the σ-holes of **3^Ch^**

Entry	Compound	C(benzim)—Ch1^*a*^	C(benzim)—Ch2^*a*^	CH_3_—Ch1^*a*^	CH_3_—Ch2^*a*^	C(core)—H^*b*^	Maximum
1	**3^Te^**	152.4	153.3	147.8	147.2	145.2	–
2	**3^Se^**	146.6	144.7	145.9	145.6	146.9^*c*^	147.9
3	**3^S^**	141.0	144.2	144.5	144.9^*c*^	146.6^*c*^	149.1

Notes: (*a*) surface map values in kcal mol^−1^ in the elongation of the respective bonds, (*b*) the H atom at the 2-position of the central benzene core and (*c*) approximate values due to the unclear definition of the σ-holes.
