# Supplementary material for: Chalcogen bonding in the solid-state structures of 1,3-bis(benzimidazoliumyl)benzene-based chalcogen-bonding donors
Source: Acta Crystallogr C Struct Chem. 2023 Jan 11;79(Pt 2):26–35. doi: 10.1107/S2053229622011536 (PMC9899511; doi:10.1107/S2053229622011536)
Supplement: Supplementary file 8 [file c-79-00026-sup8.pdf]

STRUCTURAL CHEMISTRY

Volume 79 (2023)

Supporting information for article:

Chalcogen bonding in the solid-state structures of 1,3-bis(benzimidazoliumyl)benzene-based chalcogen-bonding donors

Tim Steinke, Elric Engelage and Stefan M. Huber

S1. Crystal structure Computing details

S1.1. Data of 3^S

S1.1.1. Crystal Data 3^S

$C_{26}H_{24}F_6N_4O_6S_4$
M_r = 730.73 g/mol
Monoclinic P2$_1$/c
a = 7.7776(2) Å
b = 22.5823(6) Å
c = 18.2338(5) Å
β = 99.329(2)°
V = 3160.16(15) Å^3
z = 4
F(000) = 1496

D_x = 1.536 g/cm^3
Cu K_α radiation, λ = 1.54184 Å
Cell parameters from 5564 reflections
θ = 3.1 - 66.5°
μ = 3.511 mm^{-1}
T = 169.99 K
Needle, translucent white
0.134 x 0.055 x 0.030 mm

S1.2. Data collection 3^S

XtaLAB Synergy, Dualflex, HyPix
Radiation source: PhotonJet (Cu) X-ray Source
Mirror monochromator
ω scans
Absorption correction: Multi-scan CrysAlis PRO 1.171.40.20a (Rigaku Oxford Diffraction, 2018) Empirical absorption correction using spherical harmonics, implemented in SCALE3 ABSPACK scaling algorithm.

Tmin = 0.579, Tmax = 1.000
21606 measured reflections
5564 independent reflections
4631 reflections with I > 2σ(I)
R_{int} = 4.3%
θ_{max} = 66.5°, θ_{min} = 3.1°
h = -9 → 5
k = -25 → 26
l = -21 → 21

S1.3. Refinement 3^S

Refinement on F2
Least-squares matrix: full
R[F2 > 2σ(F2)] = 7.3%
wR(F2) = 20.5%
S = 1.094
5564 reflections

419 parameters
0 restraints
$\Delta\rho_{max}$, $\Delta\rho_{min}$ (e Å^{-3}) = 0.69, −0.39
Primary atom site location: dual
Hydrogen site location: constrained

Table S1 3^S

Atomic coordinates and equivalent isotropic displacement parameters (Å^2) for **3^S**.

	x	y	z	U(eq)
S(1)	0.35126(16)	0.56835(6)	0.25113(7)	0.0608(4)
N(1)	0.3348(5)	0.50402(16)	0.3780(2)	0.0540(10)

O(1)	-0.0399(6)	0.41389(17)	0.2140(3)	0.0872(13)
F(1)	0.0689(4)	0.51149(15)	0.12752(18)	0.0726(8)
C(1)	0.5501(7)	0.6107(3)	0.2717(3)	0.0715(15)
C(2)	0.3914(7)	0.4486(2)	0.3489(4)	0.0719(16)
S(2)	0.49729(19)	0.80031(6)	0.25935(8)	0.0670(4)
N(2)	0.2444(4)	0.59565(15)	0.3825(2)	0.0443(8)
O(2)	-0.2997(5)	0.4683(2)	0.2232(3)	0.0866(13)
F(2)	-0.1862(5)	0.48431(17)	0.0786(2)	0.0926(11)
S(3)	-0.11679(15)	0.47000(5)	0.22368(7)	0.0541(3)
N(3)	0.3109(5)	0.80886(15)	0.3752(2)	0.0470(9)
O(3)	-0.0234(5)	0.50764(17)	0.2798(2)	0.0698(10)
F(3)	-0.1486(5)	0.56541(14)	0.1402(2)	0.0952(12)
C(3)	0.3109(5)	0.55545(19)	0.3401(3)	0.0494(11)
C(4)	0.2803(5)	0.5108(2)	0.4456(3)	0.0520(11)
S(4)	0.77237(17)	0.72091(5)	0.48588(8)	0.0614(4)
N(4)	0.5248(5)	0.87227(16)	0.3815(2)	0.0536(10)
O(4)	0.6806(7)	0.66722(19)	0.4697(4)	0.118(2)
F(4)	0.7760(9)	0.7011(3)	0.6268(3)	0.157(2)
C(5)	0.2729(7)	0.4715(2)	0.5046(4)	0.0674(16)
O(5)	0.9570(5)	0.71609(19)	0.5008(3)	0.0824(12)
F(5)	0.5543(10)	0.7495(4)	0.5733(4)	0.197(4)
O(6)	0.7038(6)	0.76858(18)	0.4399(3)	0.0870(13)
F(6)	0.8004(12)	0.7926(3)	0.5986(3)	0.183(3)
C(6)	0.2087(7)	0.4934(3)	0.5638(3)	0.0711(16)
C(7)	0.1544(7)	0.5519(3)	0.5683(3)	0.0658(14)
C(8)	0.1598(6)	0.5913(2)	0.5103(3)	0.0560(12)
C(9)	0.2250(5)	0.56933(19)	0.4506(3)	0.0470(10)
C(25)	-0.0943(6)	0.5092(2)	0.1387(3)	0.0575(12)
C(10)	0.1780(5)	0.65309(18)	0.3587(2)	0.0427(9)
C(13)	0.0411(6)	0.7624(2)	0.3156(3)	0.0540(11)
C(12)	-0.0553(7)	0.7121(2)	0.2967(3)	0.0618(13)
C(11)	0.0119(6)	0.6568(2)	0.3173(3)	0.0540(11)
C(15)	0.2777(5)	0.70264(18)	0.3781(2)	0.0431(9)
C(14)	0.2070(6)	0.75701(18)	0.3553(2)	0.0451(10)
C(16)	0.3153(6)	0.84064(18)	0.4413(3)	0.0481(10)
C(17)	0.2144(7)	0.8362(2)	0.4963(3)	0.0597(13)
C(18)	0.2588(8)	0.8743(2)	0.5563(3)	0.0683(14)
C(19)	0.3968(9)	0.9144(2)	0.5590(3)	0.0742(17)
C(26)	0.7166(13)	0.7413(5)	0.5744(5)	0.111(3)
C(20)	0.4967(7)	0.9181(2)	0.5047(3)	0.0645(14)
C(21)	0.4523(6)	0.87969(17)	0.4445(3)	0.0501(11)
C(22)	0.6779(7)	0.9045(2)	0.3644(4)	0.0718(16)
C(23)	0.4388(6)	0.82879(18)	0.3403(3)	0.0493(10)
C(24)	0.3058(10)	0.8150(3)	0.1931(4)	0.091(2)
H(1A)	0.579226	0.627305	0.225622	0.107
H(1AB)	0.644929	0.584853	0.294772	0.107
H(1AC)	0.534172	0.642930	0.305987	0.107
H(2A)	0.289278	0.425239	0.327712	0.108
H(2AB)	0.461306	0.426270	0.389191	0.108

H(2AC)	0.461914	0.457087	0.310199	0.108
H(5)	0.310860	0.431642	0.503126	0.081
H(6)	0.200375	0.467634	0.604257	0.085
H(7)	0.112974	0.565088	0.611725	0.079
H(8)	0.120478	0.631015	0.511917	0.067
H(13)	-0.005869	0.800385	0.301595	0.065
H(12)	-0.169567	0.715418	0.269176	0.074
H(11)	-0.054144	0.622075	0.303404	0.065
H(15)	0.391374	0.699598	0.406269	0.052
H(17)	0.120517	0.808961	0.493525	0.072
H(18)	0.194119	0.873057	0.596247	0.082
H(19)	0.421558	0.940111	0.600630	0.089
H(20)	0.591175	0.945075	0.507464	0.077
H(22A)	0.706455	0.890325	0.317005	0.108
H(22B)	0.777133	0.897555	0.403977	0.108
H(22C)	0.652004	0.946926	0.360821	0.108
H(24A)	0.334857	0.813690	0.142782	0.137
H(24B)	0.260693	0.854357	0.202400	0.137
H(24C)	0.217090	0.785069	0.197802	0.137

Anisotropic displacement parameters [Å^2] for **3**^{S}.

	U11	U22	U33	U23	U13	U12
S(1)	0.0561(7)	0.0656(8)	0.0585(7)	-0.0152(6)	0.0024(5)	-0.0077(6)
N(1)	0.044(2)	0.0352(19)	0.075(3)	-0.0064(18)	-0.0119(19)	-0.0007(15)
O(1)	0.117(3)	0.044(2)	0.107(3)	0.012(2)	0.038(3)	0.011(2)
F(1)	0.0648(18)	0.076(2)	0.076(2)	0.0123(16)	0.0057(15)	-0.0092(15)
C(1)	0.065(3)	0.073(4)	0.073(4)	-0.003(3)	0.000(3)	-0.024(3)
C(2)	0.065(3)	0.040(3)	0.105(5)	-0.011(3)	-0.007(3)	0.008(2)
S(2)	0.0748(8)	0.0602(8)	0.0694(9)	0.0042(6)	0.0218(7)	0.0106(6)
N(2)	0.0456(19)	0.0336(17)	0.050(2)	-0.0005(15)	-0.0049(15)	-0.0044(14)
O(2)	0.059(2)	0.104(3)	0.099(3)	-0.023(3)	0.017(2)	-0.018(2)
F(2)	0.104(3)	0.090(2)	0.071(2)	-0.0061(18)	-0.0237(19)	-0.027(2)
S(3)	0.0519(6)	0.0430(6)	0.0658(7)	-0.0067(5)	0.0044(5)	-0.0074(5)
N(3)	0.050(2)	0.0331(18)	0.056(2)	-0.0019(16)	0.0019(17)	-0.0003(15)
O(3)	0.075(2)	0.068(2)	0.061(2)	-0.0094(18)	-0.0018(18)	-0.0241(18)
F(3)	0.115(3)	0.0526(19)	0.110(3)	0.0145(18)	-0.008(2)	0.0221(18)
C(3)	0.039(2)	0.038(2)	0.066(3)	-0.006(2)	-0.0071(19)	-0.0016(17)
C(4)	0.039(2)	0.041(2)	0.069(3)	0.007(2)	-0.011(2)	-0.0034(18)
S(4)	0.0610(7)	0.0418(6)	0.0767(9)	0.0067(6)	-0.0030(6)	0.0067(5)
N(4)	0.050(2)	0.0355(19)	0.073(3)	0.0095(18)	0.0037(19)	0.0002(16)
O(4)	0.105(4)	0.051(2)	0.172(5)	0.012(3)	-0.052(3)	-0.009(2)

F(4)	0.177(5)	0.190(6)	0.107(4)	0.063(4)	0.028(4)	0.019(4)
C(5)	0.054(3)	0.043(3)	0.094(4)	0.017(3)	-0.021(3)	-0.004(2)
O(5)	0.061(2)	0.077(3)	0.107(3)	0.002(2)	0.005(2)	0.0120(19)
F(5)	0.156(5)	0.274(9)	0.188(6)	0.087(6)	0.110(5)	0.080(6)
O(6)	0.103(3)	0.063(2)	0.093(3)	0.022(2)	0.011(2)	0.032(2)
F(6)	0.297(10)	0.146(5)	0.104(4)	-0.047(4)	0.022(5)	0.022(6)
C(6)	0.067(3)	0.070(4)	0.069(4)	0.030(3)	-0.011(3)	-0.015(3)
C(7)	0.059(3)	0.072(4)	0.061(3)	0.013(3)	-0.006(2)	-0.009(3)
C(8)	0.053(3)	0.056(3)	0.054(3)	0.009(2)	-0.004(2)	-0.001(2)
C(9)	0.043(2)	0.040(2)	0.053(3)	0.0060(19)	-0.0056(19)	-0.0036(18)
C(25)	0.056(3)	0.046(3)	0.064(3)	-0.006(2)	-0.014(2)	-0.001(2)
C(10)	0.048(2)	0.033(2)	0.044(2)	-0.0010(17)	-0.0016(18)	0.0002(17)
C(13)	0.054(3)	0.040(2)	0.064(3)	0.006(2)	-0.002(2)	0.0059(19)
C(12)	0.052(3)	0.052(3)	0.072(3)	0.002(2)	-0.017(2)	0.000(2)
C(11)	0.049(2)	0.042(2)	0.065(3)	-0.002(2)	-0.010(2)	-0.0054(19)
C(15)	0.043(2)	0.037(2)	0.046(2)	-0.0030(17)	-0.0032(17)	0.0008(17)
C(14)	0.053(2)	0.034(2)	0.047(2)	-0.0025(17)	0.0026(19)	-0.0027(18)
C(16)	0.053(2)	0.034(2)	0.054(3)	-0.0024(18)	0.001(2)	0.0054(18)
C(17)	0.067(3)	0.042(3)	0.067(3)	-0.010(2)	0.000(2)	0.000(2)
C(18)	0.089(4)	0.058(3)	0.057(3)	-0.009(2)	0.009(3)	0.006(3)
C(19)	0.096(4)	0.048(3)	0.068(4)	-0.013(3)	-0.018(3)	-0.002(3)
C(26)	0.122(7)	0.122(7)	0.094(6)	0.042(5)	0.034(5)	0.036(5)
C(20)	0.069(3)	0.039(3)	0.076(4)	-0.003(2)	-0.015(3)	-0.004(2)
C(21)	0.056(3)	0.025(2)	0.062(3)	0.0046(18)	-0.011(2)	0.0002(17)
C(22)	0.058(3)	0.053(3)	0.103(5)	0.016(3)	0.008(3)	-0.012(2)
C(23)	0.053(2)	0.034(2)	0.060(3)	0.0091(19)	0.002(2)	0.0040(18)
C(24)	0.112(5)	0.101(5)	0.059(3)	0.002(3)	0.008(3)	0.032(4)

Bond lengths [Å] and angles [°] for **3**^{S}.

S(1)-C(3)	1.726(5)	O(3)-S(3)-C(25)	102.2(2)
S(1)-C(1)	1.806(5)	C(23)-N(3)-C(16)	108.7(4)
N(1)-C(3)	1.350(6)	C(23)-N(3)-C(14)	125.8(4)
N(1)-C(4)	1.375(7)	C(16)-N(3)-C(14)	124.7(4)
N(1)-C(2)	1.455(6)	N(2)-C(3)-N(1)	108.6(4)
O(1)-S(3)	1.424(4)	N(2)-C(3)-S(1)	124.8(3)
F(1)-C(25)	1.319(6)	N(1)-C(3)-S(1)	126.6(4)

C(1)-H(1A)	0.9800	N(1)-C(4)-C(9)	107.9(4)
C(1)-H(1AB)	0.9800	N(1)-C(4)-C(5)	132.3(5)
C(1)-H(1AC)	0.9800	C(9)-C(4)-C(5)	119.7(5)
C(2)-H(2A)	0.9800	O(4)-S(4)-O(6)	113.6(3)
C(2)-H(2AB)	0.9800	O(4)-S(4)-O(5)	115.7(3)
C(2)-H(2AC)	0.9800	O(6)-S(4)-O(5)	116.3(3)
S(2)-C(23)	1.737(5)	O(4)-S(4)-C(26)	102.7(5)
S(2)-C(24)	1.790(7)	O(6)-S(4)-C(26)	102.4(3)
N(2)-C(3)	1.349(6)	O(5)-S(4)-C(26)	103.4(4)
N(2)-C(9)	1.407(6)	C(23)-N(4)-C(21)	108.8(4)
N(2)-C(10)	1.437(5)	C(23)-N(4)-C(22)	126.0(5)
O(2)-S(3)	1.422(4)	C(21)-N(4)-C(22)	125.1(4)
F(2)-C(25)	1.332(5)	C(6)-C(5)-C(4)	116.6(5)
S(3)-O(3)	1.434(4)	C(6)-C(5)-H(5)	121.7
S(3)-C(25)	1.818(6)	C(4)-C(5)-H(5)	121.7
N(3)-C(23)	1.342(6)	C(5)-C(6)-C(7)	123.2(5)
N(3)-C(16)	1.400(6)	C(5)-C(6)-H(6)	118.4
N(3)-C(14)	1.435(5)	C(7)-C(6)-H(6)	118.4
F(3)-C(25)	1.339(6)	C(8)-C(7)-C(6)	121.1(6)
C(4)-C(9)	1.397(6)	C(8)-C(7)-H(7)	119.5
C(4)-C(5)	1.403(7)	C(6)-C(7)-H(7)	119.5
S(4)-O(4)	1.414(4)	C(9)-C(8)-C(7)	115.8(5)
S(4)-O(6)	1.415(4)	C(9)-C(8)-H(8)	122.1
S(4)-O(5)	1.422(4)	C(7)-C(8)-H(8)	122.1
S(4)-C(26)	1.797(9)	C(8)-C(9)-C(4)	123.6(4)
N(4)-C(23)	1.347(6)	C(8)-C(9)-N(2)	131.0(4)
N(4)-C(21)	1.369(7)	C(4)-C(9)-N(2)	105.3(4)
N(4)-C(22)	1.471(6)	F(1)-C(25)-F(2)	106.9(5)
F(4)-C(26)	1.344(9)	F(1)-C(25)-F(3)	106.3(4)
C(5)-C(6)	1.353(9)	F(2)-C(25)-F(3)	106.7(4)
C(5)-H(5)	0.9500	F(1)-C(25)-S(3)	112.2(3)
F(5)-C(26)	1.273(10)	F(2)-C(25)-S(3)	112.5(4)
F(6)-C(26)	1.367(12)	F(3)-C(25)-S(3)	111.7(4)
C(6)-C(7)	1.394(8)	C(15)-C(10)-C(11)	121.8(4)
C(6)-H(6)	0.9500	C(15)-C(10)-N(2)	119.8(4)
C(7)-C(8)	1.387(7)	C(11)-C(10)-N(2)	118.5(4)
C(7)-H(7)	0.9500	C(12)-C(13)-C(14)	119.0(4)

C(8)-C(9)	1.365(7)	C(12)-C(13)-H(13)	120.5
C(8)-H(8)	0.9500	C(14)-C(13)-H(13)	120.5
C(10)-C(15)	1.375(6)	C(13)-C(12)-C(11)	120.8(4)
C(10)-C(11)	1.388(6)	C(13)-C(12)-H(12)	119.6
C(13)-C(12)	1.374(7)	C(11)-C(12)-H(12)	119.6
C(13)-C(14)	1.379(6)	C(12)-C(11)-C(10)	118.7(4)
C(13)-H(13)	0.9500	C(12)-C(11)-H(11)	120.6
C(12)-C(11)	1.382(7)	C(10)-C(11)-H(11)	120.6
C(12)-H(12)	0.9500	C(10)-C(15)-C(14)	117.7(4)
C(11)-H(11)	0.9500	C(10)-C(15)-H(15)	121.1
C(15)-C(14)	1.382(6)	C(14)-C(15)-H(15)	121.1
C(15)-H(15)	0.9500	C(13)-C(14)-C(15)	122.0(4)
C(16)-C(17)	1.373(7)	C(13)-C(14)-N(3)	120.0(4)
C(16)-C(21)	1.377(6)	C(15)-C(14)-N(3)	117.9(4)
C(17)-C(18)	1.390(7)	C(17)-C(16)-C(21)	123.1(4)
C(17)-H(17)	0.9500	C(17)-C(16)-N(3)	130.9(4)
C(18)-C(19)	1.399(9)	C(21)-C(16)-N(3)	106.0(4)
C(18)-H(18)	0.9500	C(16)-C(17)-C(18)	115.5(5)
C(19)-C(20)	1.357(9)	C(16)-C(17)-H(17)	122.2
C(19)-H(19)	0.9500	C(18)-C(17)-H(17)	122.2
C(20)-C(21)	1.398(7)	C(17)-C(18)-C(19)	121.3(6)
C(20)-H(20)	0.9500	C(17)-C(18)-H(18)	119.4
C(22)-H(22A)	0.9800	C(19)-C(18)-H(18)	119.4
C(22)-H(22B)	0.9800	C(20)-C(19)-C(18)	122.7(5)
C(22)-H(22C)	0.9800	C(20)-C(19)-H(19)	118.6
C(24)-H(24A)	0.9800	C(18)-C(19)-H(19)	118.6
C(24)-H(24B)	0.9800	F(5)-C(26)-F(4)	109.5(8)
C(24)-H(24C)	0.9800	F(5)-C(26)-F(6)	107.5(9)
C(3)-S(1)-C(1)	100.0(2)	F(4)-C(26)-F(6)	104.6(9)
C(3)-N(1)-C(4)	109.1(4)	F(5)-C(26)-S(4)	114.0(8)
C(3)-N(1)-C(2)	125.2(5)	F(4)-C(26)-S(4)	111.1(6)
C(4)-N(1)-C(2)	125.4(4)	F(6)-C(26)-S(4)	109.6(6)
S(1)-C(1)-H(1A)	109.5	C(19)-C(20)-C(21)	115.9(5)
S(1)-C(1)-H(1AB)	109.5	C(19)-C(20)-H(20)	122.0
H(1A)-C(1)-H(1AB)	109.5	C(21)-C(20)-H(20)	122.0
S(1)-C(1)-H(1AC)	109.5	N(4)-C(21)-C(16)	107.9(4)
H(1A)-C(1)-H(1AC)	109.5	N(4)-C(21)-C(20)	130.7(5)

H(1AB)-C(1)-H(1AC)	109.5	C(16)-C(21)-C(20)	121.4(5)
N(1)-C(2)-H(2A)	109.5	N(4)-C(22)-H(22A)	109.5
N(1)-C(2)-H(2AB)	109.5	N(4)-C(22)-H(22B)	109.5
H(2A)-C(2)-H(2AB)	109.5	H(22A)-C(22)-H(22B)	109.5
N(1)-C(2)-H(2AC)	109.5	N(4)-C(22)-H(22C)	109.5
H(2A)-C(2)-H(2AC)	109.5	H(22A)-C(22)-H(22C)	109.5
H(2AB)-C(2)-H(2AC)	109.5	H(22B)-C(22)-H(22C)	109.5
C(23)-S(2)-C(24)	101.6(3)	N(3)-C(23)-N(4)	108.6(4)
C(3)-N(2)-C(9)	109.2(4)	N(3)-C(23)-S(2)	126.5(3)
C(3)-N(2)-C(10)	125.9(4)	N(4)-C(23)-S(2)	124.7(4)
C(9)-N(2)-C(10)	124.3(4)	S(2)-C(24)-H(24A)	109.5
O(2)-S(3)-O(1)	114.5(3)	S(2)-C(24)-H(24B)	109.5
O(2)-S(3)-O(3)	114.3(3)	H(24A)-C(24)-H(24B)	109.5
O(1)-S(3)-O(3)	116.2(3)	S(2)-C(24)-H(24C)	109.5
O(2)-S(3)-C(25)	104.0(3)	H(24A)-C(24)-H(24C)	109.5
O(1)-S(3)-C(25)	103.2(2)	H(24B)-C(24)-H(24C)	109.5

Symmetry transformations used to generate equivalent atoms.

S1.4. Data of 3^{Se} - A

S1.4.1. Crystal Data 3^{Se} - A

$C_{26}H_{24}F_6N_4O_6S_2Se_2$
M_r = 824.53 g/mol
Monoclinic $P2_1/c$
a = 7.74775(4) Å
b = 13.12863(8) Å
c = 31.27496(18) Å
β = 90.6141(5)°
V = 3181.02(3) $Å^3$
z = 4
F(000) = 1640
D_x = 1.722 g/cm^3

Cu K_α radiation, λ = 1.54184 Å
Cell parameters from 5613 reflections
θ = 2.8 - 66.5°
μ = 4.88 mm^{-1}
T = 170.00 K
Cut prism, clear colourless
0.286 x 0.142 x 0.075 mm

S1.5. Data collection 3^{Se} - A

XtaLAB Synergy, Dualflex, HyPix
Radiation source: Mirror
ω scans
Absorption correction: Gaussian
CrysAlis PRO 1.171.40.20a (Rigaku Oxford Diffraction, 2018) Numerical absorption correction based on gaussian integration over a multifaceted crystal model Empirical absorption correction using spherical harmonics, implemented in SCALE3 ABSPACK scaling algorithm.
Tmin = 0.30, Tmax = 1.00
37979 measured reflections
5613 independent reflections
5268 reflections with I > 2σ(I)
R_{int} = 3.9%

θ_{max} = 66.5°, θ_{min} = 2.8°
h = -9 → 8
k = -15 → 15
l = -33 → 37

S1.6. Refinement 3^{Se} - A

Refinement on F2
Least-squares matrix: full
R[F2 > 2σ(F2)] = 2.8%
wR(F2) = 7.0%
S = 1.02
5613 reflections
468 parameters
0 restraints
Primary atom site location: dual
Hydrogen site location: constrained

Table S2 3^{Se}- A

Atomic coordinates and equivalent isotropic displacement parameters (Å^2) for **3^{Se} - A**.

	x	y	z	U(eq)
Se(1)	0.65737(3)	0.42578(2)	0.43767(2)	0.03035(8)
Se(2)	0.65188(3)	0.64005(2)	0.34377(2)	0.03505(8)
N(1)	0.5833(2)	0.20696(14)	0.44266(6)	0.0270(4)
N(2)	0.3857(2)	0.29649(13)	0.40949(5)	0.0228(4)
N(3)	0.3914(2)	0.53847(14)	0.29349(5)	0.0250(4)
N(4)	0.5899(2)	0.60572(14)	0.25347(6)	0.0277(4)
C(1)	0.7135(4)	0.4171(2)	0.49873(8)	0.0433(6)
C(2)	0.4748(4)	0.7348(3)	0.36256(10)	0.0541(7)
C(3)	0.5385(3)	0.30088(16)	0.43047(6)	0.0244(4)
C(4)	0.7418(3)	0.1769(2)	0.46527(9)	0.0439(6)
C(5)	0.4536(3)	0.13950(17)	0.43035(7)	0.0286(5)
C(6)	0.4380(4)	0.03435(18)	0.43572(8)	0.0382(6)
C(7)	0.2925(4)	-0.0094(2)	0.41803(9)	0.0466(7)
C(8)	0.1666(4)	0.0474(2)	0.39622(9)	0.0451(7)
C(9)	0.1819(3)	0.15228(19)	0.39078(8)	0.0350(5)
C(10)	0.3293(3)	0.19581(16)	0.40876(7)	0.0263(4)
C(11)	0.5374(3)	0.59428(17)	0.29369(7)	0.0259(4)
C(12)	0.7436(3)	0.6609(2)	0.23925(9)	0.0418(6)
C(13)	0.4767(3)	0.55470(16)	0.22628(7)	0.0274(4)
C(14)	0.4739(4)	0.5422(2)	0.18222(8)	0.0405(6)
C(15)	0.3396(4)	0.4855(2)	0.16546(8)	0.0467(7)
C(16)	0.2127(4)	0.4419(2)	0.19127(8)	0.0420(6)

C(17)	0.2144(3)	0.45407(19)	0.23485(7)	0.0324(5)
C(18)	0.3495(3)	0.51175(16)	0.25161(6)	0.0252(4)
C(19)	0.3045(3)	0.38090(16)	0.38795(6)	0.0226(4)
C(20)	0.1522(3)	0.42223(17)	0.40319(7)	0.0272(5)
C(21)	0.0786(3)	0.50337(19)	0.38142(7)	0.0318(5)
C(22)	0.1547(3)	0.54272(19)	0.34491(7)	0.0301(5)
C(23)	0.3072(3)	0.49972(17)	0.33100(6)	0.0246(4)
C(24)	0.3829(3)	0.41797(16)	0.35164(6)	0.0236(4)
S(2)	0.23709(8)	0.27181(4)	0.53076(2)	0.03371(14)
F(4)	0.1481(3)	0.14818(19)	0.59164(7)	0.0915(8)
F(5)	-0.0647(2)	0.22005(16)	0.55977(7)	0.0693(5)
F(6)	0.0696(3)	0.09949(15)	0.52938(9)	0.0915(7)
O(4)	0.2348(3)	0.35229(15)	0.56160(7)	0.0536(5)
O(5)	0.1547(3)	0.29449(18)	0.49060(6)	0.0630(6)
O(6)	0.3970(3)	0.21663(19)	0.52873(7)	0.0632(6)
C(26)	0.0923(4)	0.1797(2)	0.55375(9)	0.0483(7)
S(1A)	0.82979(10)	0.33445(8)	0.30604(3)	0.0304(3)
F(1A)	0.8104(4)	0.2125(2)	0.23925(10)	0.0611(8)
F(2A)	0.5919(5)	0.3100(3)	0.24631(12)	0.0580(9)
F(3A)	0.8292(3)	0.3715(2)	0.22338(7)	0.0585(7)
O(1A)	0.7200(5)	0.2707(3)	0.33147(13)	0.0531(9)
O(2A)	1.0134(13)	0.3105(6)	0.3063(2)	0.0341(11)
O(3A)	0.7920(3)	0.44154(19)	0.30852(10)	0.0446(7)
C(27A)	0.7659(14)	0.3056(8)	0.2506(3)	0.039(2)
C(27B)	0.8672(13)	0.2688(10)	0.3114(3)	0.039(2)
O(1B)	0.7976(10)	0.4328(6)	0.2719(4)	0.069(3)
O(2B)	0.5834(15)	0.2999(10)	0.2679(4)	0.049(3)
O(3B)	0.8581(12)	0.2808(11)	0.2293(3)	0.066(3)
F(1B)	0.7846(11)	0.2951(9)	0.3465(3)	0.070(3)
F(2B)	1.022(3)	0.2947(16)	0.3152(6)	0.0341(11)
F(3B)	0.8668(9)	0.1687(5)	0.3093(2)	0.064(2)
S(1B)	0.7620(8)	0.3257(5)	0.26391(18)	0.0344(11)
H(1A)	0.620462	0.381536	0.513578	0.065
H(1AB)	0.726361	0.485887	0.510494	0.065
H(1AC)	0.821774	0.379496	0.502677	0.065

H(2A)	0.512508	0.767708	0.389198	0.081
H(2AB)	0.367043	0.697746	0.367425	0.081
H(2AC)	0.455801	0.786789	0.340499	0.081
H(4A)	0.719025	0.171376	0.495941	0.066
H(4AB)	0.831422	0.228253	0.460559	0.066
H(4AC)	0.781135	0.110890	0.454429	0.066
H(6)	0.522600	-0.004595	0.450651	0.046
H(7)	0.276908	-0.080869	0.420733	0.056
H(8)	0.068092	0.013546	0.384810	0.054
H(9)	0.097531	0.191347	0.375827	0.042
H(12A)	0.789534	0.702111	0.262879	0.063
H(12B)	0.712557	0.705366	0.215211	0.063
H(12C)	0.831466	0.611974	0.230146	0.063
H(14)	0.559900	0.571217	0.164547	0.049
H(15)	0.332824	0.475704	0.135395	0.056
H(16)	0.123143	0.402895	0.178266	0.050
H(17)	0.128460	0.424864	0.252494	0.039
H(20)	0.099529	0.395370	0.428055	0.033
H(21)	-0.025378	0.532601	0.391576	0.038
H(22)	0.103140	0.597916	0.329851	0.036
H(24)	0.485820	0.388053	0.341198	0.028

Anisotropic displacement parameters [Å^2] for **3^{Se} – A.**

	U11	U22	U33	U23	U13	U12
Se(1)	0.02959(13)	0.03091(14)	0.03050(13)	0.00076(9)	-0.00250(9)	-0.00819(9)
Se(2)	0.02722(14)	0.04291(16)	0.03488(14)	-0.00055(10)	-0.00660(10)	-0.00386(10)
N(1)	0.0296(9)	0.0268(9)	0.0246(9)	0.0016(7)	0.0010(7)	0.0009(7)
N(2)	0.0228(8)	0.0257(9)	0.0200(8)	0.0006(7)	0.0038(6)	-0.0035(7)
N(3)	0.0219(8)	0.0336(10)	0.0197(8)	0.0043(7)	0.0009(7)	-0.0020(7)
N(4)	0.0256(9)	0.0274(9)	0.0301(9)	0.0040(7)	0.0068(7)	-0.0024(7)
C(1)	0.0525(16)	0.0437(15)	0.0334(13)	-0.0058(11)	-0.0106(11)	-0.0052(12)
C(2)	0.0454(16)	0.0613(19)	0.0554(17)	-0.0225(15)	-0.0078(13)	0.0049(14)
C(3)	0.0240(10)	0.0295(11)	0.0198(10)	0.0000(8)	0.0029(8)	-0.0018(8)
C(4)	0.0410(14)	0.0407(14)	0.0498(15)	0.0033(12)	-0.0116(12)	0.0086(11)
C(5)	0.0349(12)	0.0283(11)	0.0228(10)	-0.0016(8)	0.0087(9)	-0.0029(9)
C(6)	0.0532(15)	0.0289(12)	0.0327(12)	0.0031(10)	0.0138(11)	-0.0001(11)

C(7)	0.0620(17)	0.0290(13)	0.0492(15)	-0.0035(11)	0.0215(13)	-0.0147(12)
C(8)	0.0452(15)	0.0422(14)	0.0482(15)	-0.0110(12)	0.0125(12)	-0.0210(12)
C(9)	0.0325(12)	0.0395(13)	0.0332(12)	-0.0061(10)	0.0075(10)	-0.0090(10)
C(10)	0.0296(11)	0.0268(11)	0.0226(10)	-0.0015(8)	0.0096(8)	-0.0064(9)
C(11)	0.0220(10)	0.0289(11)	0.0267(11)	0.0027(9)	0.0014(8)	0.0017(8)
C(12)	0.0363(13)	0.0434(14)	0.0460(14)	0.0029(11)	0.0154(11)	-0.0139(11)
C(13)	0.0317(11)	0.0250(11)	0.0255(11)	0.0023(8)	0.0056(9)	-0.0002(9)
C(14)	0.0541(15)	0.0412(14)	0.0265(12)	0.0019(10)	0.0131(11)	-0.0077(12)
C(15)	0.0673(18)	0.0500(16)	0.0229(12)	-0.0045(11)	0.0064(11)	-0.0142(14)
C(16)	0.0533(16)	0.0437(14)	0.0290(12)	-0.0024(10)	-0.0018(11)	-0.0132(12)
C(17)	0.0305(11)	0.0409(13)	0.0258(11)	0.0059(9)	0.0009(9)	-0.0066(10)
C(18)	0.0275(10)	0.0281(11)	0.0201(10)	0.0040(8)	0.0020(8)	0.0016(9)
C(19)	0.0214(10)	0.0275(11)	0.0190(9)	-0.0016(8)	0.0001(8)	-0.0038(8)
C(20)	0.0227(10)	0.0388(12)	0.0201(10)	0.0012(9)	0.0039(8)	-0.0024(9)
C(21)	0.0223(10)	0.0454(13)	0.0278(11)	0.0009(10)	0.0055(8)	0.0054(10)
C(22)	0.0239(11)	0.0394(13)	0.0269(11)	0.0040(9)	-0.0015(9)	0.0032(9)
C(23)	0.0216(10)	0.0342(11)	0.0181(10)	0.0015(8)	0.0017(8)	-0.0054(9)
C(24)	0.0180(9)	0.0319(11)	0.0209(10)	0.0000(8)	0.0020(8)	-0.0011(8)
S(2)	0.0418(3)	0.0356(3)	0.0239(3)	-0.0002(2)	0.0078(2)	-0.0108(2)
F(4)	0.0806(14)	0.1174(18)	0.0760(14)	0.0721(14)	-0.0233(11)	-0.0289(13)
F(5)	0.0361(9)	0.0898(14)	0.0821(13)	0.0215(11)	0.0008(8)	-0.0170(9)
F(6)	0.1023(17)	0.0403(10)	0.131(2)	-0.0046(12)	-0.0255(15)	-0.0263(11)
O(4)	0.0619(13)	0.0463(11)	0.0525(12)	-0.0187(9)	0.0055(9)	-0.0137(9)
O(5)	0.0956(17)	0.0673(14)	0.0263(9)	0.0162(9)	0.0000(10)	-0.0012(12)
O(6)	0.0446(11)	0.0844(16)	0.0609(13)	-0.0131(12)	0.0140(10)	0.0016(11)
C(26)	0.0465(16)	0.0443(16)	0.0538(16)	0.0205(13)	-0.0152(12)	-0.0142(12)
S(1A)	0.0247(4)	0.0284(6)	0.0381(5)	-0.0052(3)	0.0045(3)	0.0023(3)
F(1A)	0.0574(16)	0.0514(15)	0.0745(18)	-0.0349(14)	0.0013(13)	-0.0056(13)
F(2A)	0.0324(14)	0.0642(19)	0.077(2)	-0.013(2)	-0.0141(19)	-0.0109(12)
F(3A)	0.0561(15)	0.0740(19)	0.0452(13)	0.0064(12)	-0.0094(11)	-0.0226(14)
O(1A)	0.046(2)	0.0530(18)	0.060(2)	0.0019(17)	0.0217(17)	-0.0061(16)
O(2A)	0.0296(13)	0.033(3)	0.040(4)	0.0030(18)	0.003(2)	0.0034(18)
O(3A)	0.0425(14)	0.0325(13)	0.0587(18)	-0.0168(12)	-0.0093(13)	0.0090(11)
C(27A)	0.034(3)	0.037(4)	0.046(5)	-0.010(3)	-0.001(4)	-0.005(3)
C(27B)	0.046(6)	0.040(7)	0.031(5)	-0.006(4)	-0.016(4)	0.014(5)
O(1B)	0.038(4)	0.031(4)	0.136(10)	0.011(5)	-0.018(5)	0.002(3)
O(2B)	0.026(4)	0.054(5)	0.066(7)	-0.008(6)	-0.009(5)	-0.003(3)

O(3B)	0.053(5)	0.117(12)	0.027(4)	-0.022(5)	0.002(4)	0.018(6)
F(1B)	0.045(5)	0.124(9)	0.041(4)	-0.004(4)	0.014(3)	0.017(5)
F(2B)	0.0296(13)	0.033(3)	0.040(4)	0.0030(18)	0.003(2)	0.0034(18)
F(3B)	0.059(4)	0.038(3)	0.095(5)	0.018(3)	-0.021(3)	-0.009(3)
S(1B)	0.0244(14)	0.034(2)	0.045(3)	0.0025(19)	0.003(2)	0.0040(15)

Bond lengths [Å] and angles [°] for **3**^{Se} **- A**.

Se(1)-C(3)	1.893(2)	N(2)-C(3)-Se(1)	121.22(16)
Se(1)-C(1)	1.957(2)	N(1)-C(4)-H(4A)	109.5
Se(2)-C(11)	1.890(2)	N(1)-C(4)-H(4AB)	109.5
Se(2)-C(2)	1.948(3)	H(4A)-C(4)-H(4AB)	109.5
N(1)-C(3)	1.335(3)	N(1)-C(4)-H(4AC)	109.5
N(1)-C(5)	1.391(3)	H(4A)-C(4)-H(4AC)	109.5
N(1)-C(4)	1.465(3)	H(4AB)-C(4)-H(4AC)	109.5
N(2)-C(3)	1.349(3)	C(10)-C(5)-N(1)	106.97(19)
N(2)-C(10)	1.392(3)	C(10)-C(5)-C(6)	121.7(2)
N(2)-C(19)	1.438(3)	N(1)-C(5)-C(6)	131.3(2)
N(3)-C(11)	1.348(3)	C(7)-C(6)-C(5)	115.9(3)
N(3)-C(18)	1.391(3)	C(7)-C(6)-H(6)	122.1
N(3)-C(23)	1.441(3)	C(5)-C(6)-H(6)	122.1
N(4)-C(11)	1.335(3)	C(6)-C(7)-C(8)	122.5(2)
N(4)-C(13)	1.388(3)	C(6)-C(7)-H(7)	118.8
N(4)-C(12)	1.467(3)	C(8)-C(7)-H(7)	118.8
C(1)-H(1A)	0.9800	C(9)-C(8)-C(7)	121.8(2)
C(1)-H(1AB)	0.9800	C(9)-C(8)-H(8)	119.1
C(1)-H(1AC)	0.9800	C(7)-C(8)-H(8)	119.1
C(2)-H(2A)	0.9800	C(10)-C(9)-C(8)	115.3(2)
C(2)-H(2AB)	0.9800	C(10)-C(9)-H(9)	122.3
C(2)-H(2AC)	0.9800	C(8)-C(9)-H(9)	122.3
C(4)-H(4A)	0.9800	C(5)-C(10)-C(9)	122.8(2)
C(4)-H(4AB)	0.9800	C(5)-C(10)-N(2)	106.42(19)
C(4)-H(4AC)	0.9800	C(9)-C(10)-N(2)	130.8(2)
C(5)-C(10)	1.384(3)	N(4)-C(11)-N(3)	108.73(18)
C(5)-C(6)	1.396(3)	N(4)-C(11)-Se(2)	126.88(16)
C(6)-C(7)	1.376(4)	N(3)-C(11)-Se(2)	124.28(15)
C(6)-H(6)	0.9500	N(4)-C(12)-H(12A)	109.5
C(7)-C(8)	1.400(4)	N(4)-C(12)-H(12B)	109.5

C(7)-H(7)	0.9500	H(12A)-C(12)-H(12B)	109.5
C(8)-C(9)	1.392(4)	N(4)-C(12)-H(12C)	109.5
C(8)-H(8)	0.9500	H(12A)-C(12)-H(12C)	109.5
C(9)-C(10)	1.390(3)	H(12B)-C(12)-H(12C)	109.5
C(9)-H(9)	0.9500	N(4)-C(13)-C(14)	131.9(2)
C(12)-H(12A)	0.9800	N(4)-C(13)-C(18)	107.11(18)
C(12)-H(12B)	0.9800	C(14)-C(13)-C(18)	121.0(2)
C(12)-H(12C)	0.9800	C(15)-C(14)-C(13)	116.4(2)
C(13)-C(14)	1.388(3)	C(15)-C(14)-H(14)	121.8
C(13)-C(18)	1.390(3)	C(13)-C(14)-H(14)	121.8
C(14)-C(15)	1.378(4)	C(14)-C(15)-C(16)	122.2(2)
C(14)-H(14)	0.9500	C(14)-C(15)-H(15)	118.9
C(15)-C(16)	1.401(4)	C(16)-C(15)-H(15)	118.9
C(15)-H(15)	0.9500	C(17)-C(16)-C(15)	121.7(2)
C(16)-C(17)	1.372(3)	C(17)-C(16)-H(16)	119.2
C(16)-H(16)	0.9500	C(15)-C(16)-H(16)	119.2
C(17)-C(18)	1.390(3)	C(16)-C(17)-C(18)	115.9(2)
C(17)-H(17)	0.9500	C(16)-C(17)-H(17)	122.0
C(19)-C(24)	1.382(3)	C(18)-C(17)-H(17)	122.0
C(19)-C(20)	1.388(3)	C(17)-C(18)-C(13)	122.8(2)
C(20)-C(21)	1.384(3)	C(17)-C(18)-N(3)	131.26(19)
C(20)-H(20)	0.9500	C(13)-C(18)-N(3)	105.94(18)
C(21)-C(22)	1.390(3)	C(24)-C(19)-C(20)	121.8(2)
C(21)-H(21)	0.9500	C(24)-C(19)-N(2)	117.53(18)
C(22)-C(23)	1.384(3)	C(20)-C(19)-N(2)	120.64(18)
C(22)-H(22)	0.9500	C(21)-C(20)-C(19)	118.65(19)
C(23)-C(24)	1.380(3)	C(21)-C(20)-H(20)	120.7
C(24)-H(24)	0.9500	C(19)-C(20)-H(20)	120.7
S(2)-O(4)	1.4308(19)	C(20)-C(21)-C(22)	120.9(2)
S(2)-O(5)	1.434(2)	C(20)-C(21)-H(21)	119.5
S(2)-O(6)	1.437(2)	C(22)-C(21)-H(21)	119.5
S(2)-C(26)	1.804(3)	C(23)-C(22)-C(21)	118.5(2)
F(4)-C(26)	1.323(3)	C(23)-C(22)-H(22)	120.7
F(5)-C(26)	1.342(4)	C(21)-C(22)-H(22)	120.7
F(6)-C(26)	1.311(4)	C(24)-C(23)-C(22)	122.01(19)
S(1A)-O(3A)	1.438(3)	C(24)-C(23)-N(3)	117.49(18)
S(1A)-O(1A)	1.439(4)	C(22)-C(23)-N(3)	120.49(19)

S(1A)-O(2A)	1.456(9)	C(23)-C(24)-C(19)	118.02(19)
S(1A)-C(27A)	1.837(9)	C(23)-C(24)-H(24)	121.0
F(1A)-C(27A)	1.320(9)	C(19)-C(24)-H(24)	121.0
F(2A)-C(27A)	1.355(11)	O(4)-S(2)-O(5)	115.36(14)
F(3A)-C(27A)	1.312(11)	O(4)-S(2)-O(6)	114.79(14)
C(27B)-F(2B)	1.25(3)	O(5)-S(2)-O(6)	116.21(14)
C(27B)-F(3B)	1.315(14)	O(4)-S(2)-C(26)	102.35(14)
C(27B)-F(1B)	1.323(13)	O(5)-S(2)-C(26)	102.44(13)
C(27B)-S(1B)	1.844(12)	O(6)-S(2)-C(26)	102.69(14)
O(1B)-S(1B)	1.454(10)	F(6)-C(26)-F(4)	108.1(3)
O(2B)-S(1B)	1.431(13)	F(6)-C(26)-F(5)	106.4(2)
O(3B)-S(1B)	1.445(8)	F(4)-C(26)-F(5)	106.6(3)
C(3)-Se(1)-C(1)	99.69(10)	F(6)-C(26)-S(2)	112.8(2)
C(11)-Se(2)-C(2)	97.32(11)	F(4)-C(26)-S(2)	111.59(19)
C(3)-N(1)-C(5)	108.90(18)	F(5)-C(26)-S(2)	111.08(19)
C(3)-N(1)-C(4)	126.9(2)	O(3A)-S(1A)-O(1A)	114.6(2)
C(5)-N(1)-C(4)	124.16(19)	O(3A)-S(1A)-O(2A)	114.2(4)
C(3)-N(2)-C(10)	108.78(18)	O(1A)-S(1A)-O(2A)	117.0(3)
C(3)-N(2)-C(19)	124.90(17)	O(3A)-S(1A)-C(27A)	101.5(4)
C(10)-N(2)-C(19)	126.05(18)	O(1A)-S(1A)-C(27A)	104.3(4)
C(11)-N(3)-C(18)	109.19(17)	O(2A)-S(1A)-C(27A)	102.3(5)
C(11)-N(3)-C(23)	125.12(17)	F(3A)-C(27A)-F(1A)	109.6(7)
C(18)-N(3)-C(23)	125.06(17)	F(3A)-C(27A)-F(2A)	106.6(8)
C(11)-N(4)-C(13)	109.01(18)	F(1A)-C(27A)-F(2A)	106.0(7)
C(11)-N(4)-C(12)	126.7(2)	F(3A)-C(27A)-S(1A)	112.2(6)
C(13)-N(4)-C(12)	124.24(19)	F(1A)-C(27A)-S(1A)	112.1(7)
Se(1)-C(1)-H(1A)	109.5	F(2A)-C(27A)-S(1A)	110.0(5)
Se(1)-C(1)-H(1AB)	109.5	F(2B)-C(27B)-F(3B)	106.2(13)
H(1A)-C(1)-H(1AB)	109.5	F(2B)-C(27B)-F(1B)	108.7(13)
Se(1)-C(1)-H(1AC)	109.5	F(3B)-C(27B)-F(1B)	107.5(11)
H(1A)-C(1)-H(1AC)	109.5	F(2B)-C(27B)-S(1B)	112.5(10)
H(1AB)-C(1)-H(1AC)	109.5	F(3B)-C(27B)-S(1B)	111.4(7)
Se(2)-C(2)-H(2A)	109.5	F(1B)-C(27B)-S(1B)	110.4(8)
Se(2)-C(2)-H(2AB)	109.5	O(2B)-S(1B)-O(3B)	118.4(7)
H(2A)-C(2)-H(2AB)	109.5	O(2B)-S(1B)-O(1B)	113.3(7)
Se(2)-C(2)-H(2AC)	109.5	O(3B)-S(1B)-O(1B)	115.1(9)
H(2A)-C(2)-H(2AC)	109.5	O(2B)-S(1B)-C(27B)	104.7(7)

H(2AB)-C(2)-H(2AC)	109.5	O(3B)-S(1B)-C(27B)	102.1(6)
N(1)-C(3)-N(2)	108.89(18)	O(1B)-S(1B)-C(27B)	99.9(7)
N(1)-C(3)-Se(1)	129.86(16)		

Symmetry transformations used to generate equivalent atoms.

S1.7. Data of 3^{Se} - B

S1.7.1. Crystal Data 3^{Se} - B

$C_{26}H_{24}F_6N_4O_6S_2Se_2$
M_r = 730.73 g/mol
Monoclinic $P2_1/c$
a = 13.8634(4) Å
b = 7.7558(2) Å
c = 29.3266(7) Å
β = 99.329(2)°
V = 3144.59(14) $Å^3$
z = 4
F(000) = 1640

D_x = 1.742 g/cm^3
Cu K_α radiation, λ = 1.54184 Å
Cell parameters from 5529 reflections
θ = 3.0 - 66.5°
μ = 4.93 mm^{-1}
T = 169.99 K
Needle, clear colourless
0.03 x 0.02 x 0.01 mm

S1.8. Data collection 3^{Se} - B

XtaLAB Synergy, Dualflex, HyPix
Radiation source: PhotonJet (Cu) X-ray Source
Mirror monochromator
ω scans
Absorption correction: Gaussian, CrysAlis PRO 1.171.40.20a (Rigaku Oxford Diffraction, 2018) Numerical absorption correction based on gaussian integration over a multifaceted crystal model Empirical absorption correction using spherical harmonics, implemented in SCALE3 ABSPACK scaling algorithm.

Tmin = 0.900, Tmax = 0.969
28154 measured reflections
5529 independent reflections
4865 reflections with I > 2σ(I)
R_{int} = 7.3%
θ_{max} = 66.5°, θ_{min} = 3.0°
h = -16 → 16
k = -8 → 9
l = -34 → 34

S1.9. Refinement 3^{Se} - B

Refinement on F2
Least-squares matrix: full
R[F2 > 2σ(F2)] = 5.3%
wR(F2) = 14.2%
S = 1.06
5564 reflections

419 parameters
0 restraints
$\Delta\rho_{max}$, $\Delta\rho_{min}$ (e $Å^{-3}$) = 1.21, −0.84
Primary atom site location: dual
Hydrogen site location: constrained

The needles suitable for x-ray diffraction were generally polycrystalline and could not be separated into single crystals. The measured structure still contained two distinguishable diffraction patterns.

The structure was treated as part of a twin while the second set of data was not suitable for refinement.

Table S3 **3^{Se} - B.**

Atomic coordinates and equivalent isotropic displacement parameters (Å^{2}x 10^3) for **3^{Se} - B**.

	x	y	z	U(eq)
Se(1)	0.11263(3)	0.38325(5)	0.29868(2)	0.03215(15)
F(1)	0.0959(2)	0.5093(5)	0.47848(13)	0.0694(9)
N(1)	-0.0191(2)	0.2977(5)	0.36753(12)	0.0341(8)
C(1)	0.0526(4)	0.2371(8)	0.25052(17)	0.0579(14)
S(1)	0.25873(8)	0.56039(14)	0.19470(4)	0.0372(3)
O(1)	0.3333(3)	0.5611(5)	0.23149(13)	0.0578(9)
Se(2)	0.41613(3)	0.38318(6)	0.31859(2)	0.04288(17)
F(2)	0.2279(3)	0.4471(5)	0.51749(10)	0.0692(9)
N(2)	0.0917(2)	0.1025(4)	0.36094(12)	0.0294(7)
C(2)	0.5275(4)	0.4091(9)	0.2823(2)	0.0670(17)
S(2)	0.25037(8)	0.53832(14)	0.43272(4)	0.0362(2)
O(2)	0.2737(3)	0.6768(5)	0.15809(12)	0.0504(8)
O(3)	0.1633(3)	0.5591(5)	0.20962(15)	0.0626(11)
N(3)	0.4414(2)	0.0865(4)	0.37656(12)	0.0302(7)
F(3)	0.1680(2)	0.2696(4)	0.46669(10)	0.0543(7)
C(3)	0.0593(3)	0.2557(5)	0.34562(13)	0.0284(8)
O(4)	0.2589(2)	0.7146(4)	0.44704(12)	0.0461(8)
F(4)	0.2640(3)	0.2227(4)	0.19986(15)	0.0768(10)
N(4)	0.5505(2)	0.2813(4)	0.39527(13)	0.0332(7)
C(4)	-0.0785(4)	0.4534(6)	0.36051(19)	0.0455(11)
C(5)	0.4743(3)	0.2459(5)	0.36683(15)	0.0334(9)
O(5)	0.1875(3)	0.5115(5)	0.39240(11)	0.0552(9)
F(5)	0.3616(4)	0.3304(5)	0.15549(18)	0.1116(19)
C(6)	0.6053(4)	0.4434(6)	0.39690(19)	0.0468(11)
O(6)	0.3386(3)	0.4417(5)	0.43480(14)	0.0567(9)
F(6)	0.2080(5)	0.3186(6)	0.13605(17)	0.132(2)
C(7)	-0.0375(3)	0.1685(6)	0.39801(15)	0.0352(9)
C(9)	-0.1050(4)	0.0002(8)	0.4544(2)	0.0603(16)
C(8)	-0.1085(4)	0.1495(7)	0.42879(18)	0.0503(12)

C(11)	0.0402(3)	-0.1056(5)	0.42061(16)	0.0367(10)
C(10)	-0.0346(4)	-0.1258(8)	0.4502(2)	0.0582(15)
C(15)	0.6343(3)	-0.0366(7)	0.48347(15)	0.0451(12)
C(14)	0.6384(3)	0.1164(6)	0.46087(16)	0.0406(11)
C(13)	0.5690(3)	0.1418(6)	0.42471(14)	0.0335(9)
C(12)	0.0327(3)	0.0416(6)	0.39394(15)	0.0347(9)
C(18)	0.4995(3)	0.0169(5)	0.41286(14)	0.0305(8)
C(19)	0.2665(3)	0.0943(5)	0.36723(13)	0.0275(8)
C(20)	0.1806(3)	0.0211(5)	0.35003(13)	0.0290(8)
C(21)	0.1784(3)	-0.1307(5)	0.32508(15)	0.0356(9)
C(22)	0.2648(3)	-0.2086(6)	0.31623(16)	0.0393(10)
C(23)	0.3522(3)	-0.1372(5)	0.33229(15)	0.0345(9)
C(24)	0.3520(3)	0.0128(5)	0.35743(13)	0.0286(8)
C(25)	0.2726(5)	0.3490(7)	0.1695(2)	0.0605(15)
C(26)	0.1816(3)	0.4383(6)	0.47555(15)	0.0348(9)
C(17)	0.4944(3)	-0.1372(6)	0.43601(14)	0.0348(9)
C(16)	0.5645(3)	-0.1619(7)	0.47140(15)	0.0416(10)
H(1A)	0.052811	0.296917	0.221099	0.087
H(1AB)	-0.014215	0.211641	0.257104	0.087
H(1AC)	0.088992	0.129092	0.249142	0.087
H(2A)	0.505489	0.442600	0.251066	0.101
H(2AB)	0.562469	0.299384	0.281726	0.101
H(2AC)	0.570588	0.498308	0.295994	0.101
H(4A)	-0.056519	0.519097	0.334671	0.068
H(4AB)	-0.072162	0.524674	0.388172	0.068
H(4AC)	-0.146324	0.420497	0.354010	0.068
H(6A)	0.665773	0.427299	0.382119	0.070
H(6AB)	0.619983	0.477627	0.428834	0.070
H(6AC)	0.566691	0.533656	0.380849	0.070
H(9)	-0.152421	-0.017410	0.475690	0.072
H(8)	-0.156673	0.234819	0.432012	0.060
H(11)	0.091493	-0.186174	0.419121	0.044
H(10)	-0.037114	-0.228354	0.467791	0.070
H(15)	0.680543	-0.058933	0.508319	0.054
H(14)	0.685931	0.201081	0.469317	0.049

H(19)	0.267277	0.196541	0.385089	0.033
H(21)	0.118436	-0.180504	0.314207	0.043
H(22)	0.264081	-0.312285	0.298942	0.047
H(23)	0.411477	-0.190701	0.326081	0.041
H(17)	0.445942	-0.220803	0.428141	0.042
H(16)	0.565302	-0.267064	0.488023	0.050

Anisotropic displacement parameters [Å^2] for **3^{Se} - B**.

	U11	U22	U33	U23	U13	U12
Se(1)	0.0312(3)	0.0304(3)	0.0348(3)	0.00444(17)	0.00218(18)	-0.00345(16)
F(1)	0.0504(18)	0.072(2)	0.089(2)	0.0140(19)	0.0303(17)	0.0141(16)
N(1)	0.0276(17)	0.0327(18)	0.0423(19)	0.0015(15)	0.0039(14)	0.0033(14)
C(1)	0.062(3)	0.073(4)	0.039(3)	-0.001(3)	0.001(2)	-0.028(3)
S(1)	0.0404(6)	0.0362(6)	0.0363(5)	0.0051(4)	0.0109(4)	-0.0002(4)
O(1)	0.068(2)	0.054(2)	0.050(2)	-0.0003(17)	-0.0077(18)	0.0099(18)
Se(2)	0.0324(3)	0.0370(3)	0.0598(3)	0.0149(2)	0.0076(2)	0.00622(18)
F(2)	0.082(2)	0.089(2)	0.0353(15)	0.0053(16)	-0.0021(15)	-0.0044(19)
N(2)	0.0249(17)	0.0310(17)	0.0326(18)	0.0030(14)	0.0044(13)	0.0010(13)
C(2)	0.054(3)	0.085(4)	0.064(4)	0.022(3)	0.018(3)	0.014(3)
S(2)	0.0395(6)	0.0339(5)	0.0353(5)	-0.0009(4)	0.0029(4)	-0.0043(4)
O(2)	0.064(2)	0.0450(19)	0.0436(18)	0.0093(15)	0.0129(16)	-0.0139(17)
O(3)	0.051(2)	0.057(2)	0.084(3)	0.022(2)	0.031(2)	0.0100(18)
N(3)	0.0236(16)	0.0293(17)	0.0380(19)	0.0002(14)	0.0049(14)	0.0027(13)
F(3)	0.0634(19)	0.0422(15)	0.0579(17)	0.0115(13)	0.0083(14)	-0.0081(13)
C(3)	0.0238(19)	0.029(2)	0.032(2)	-0.0026(16)	-0.0017(15)	-0.0025(15)
O(4)	0.050(2)	0.0325(16)	0.055(2)	-0.0056(14)	-0.0011(15)	-0.0073(14)
F(4)	0.088(3)	0.0385(17)	0.107(3)	0.0149(18)	0.027(2)	0.0012(16)
N(4)	0.0261(17)	0.0294(17)	0.045(2)	-0.0080(15)	0.0059(14)	-0.0006(14)
C(4)	0.040(3)	0.037(2)	0.061(3)	0.008(2)	0.009(2)	0.014(2)
C(5)	0.027(2)	0.030(2)	0.044(2)	-0.0021(18)	0.0060(17)	0.0026(16)
O(5)	0.081(3)	0.048(2)	0.0348(17)	0.0041(15)	-0.0084(16)	-0.0169(18)
F(5)	0.147(4)	0.062(2)	0.140(4)	-0.013(2)	0.105(4)	0.011(3)
C(6)	0.040(3)	0.036(2)	0.064(3)	-0.014(2)	0.005(2)	-0.009(2)
O(6)	0.0429(19)	0.060(2)	0.069(2)	0.0009(19)	0.0161(17)	0.0070(17)
F(6)	0.222(7)	0.068(3)	0.096(3)	-0.025(2)	-0.061(4)	-0.024(3)
C(7)	0.027(2)	0.042(2)	0.036(2)	0.0069(19)	0.0016(16)	0.0028(18)
C(9)	0.038(3)	0.086(4)	0.059(3)	0.033(3)	0.019(2)	0.012(3)

C(8)	0.035(2)	0.065(3)	0.052(3)	0.015(2)	0.015(2)	0.012(2)
C(11)	0.033(2)	0.031(2)	0.047(3)	0.0229(18)	0.0108(19)	0.0097(17)
C(10)	0.046(3)	0.063(3)	0.067(4)	0.034(3)	0.013(3)	0.007(2)
C(15)	0.033(2)	0.070(3)	0.032(2)	-0.003(2)	0.0021(18)	0.015(2)
C(14)	0.028(2)	0.057(3)	0.037(2)	-0.012(2)	0.0040(18)	0.0027(19)
C(13)	0.027(2)	0.042(2)	0.033(2)	-0.0093(18)	0.0090(16)	0.0059(17)
C(12)	0.0238(19)	0.042(2)	0.038(2)	0.0049(18)	0.0025(16)	0.0001(17)
C(18)	0.0224(19)	0.035(2)	0.035(2)	-0.0038(17)	0.0073(15)	0.0060(16)
C(19)	0.029(2)	0.0250(18)	0.0284(19)	-0.0019(15)	0.0038(15)	-0.0004(15)
C(20)	0.029(2)	0.0273(19)	0.031(2)	0.0024(16)	0.0051(15)	0.0016(15)
C(21)	0.034(2)	0.033(2)	0.040(2)	-0.0044(17)	-0.0006(18)	-0.0052(17)
C(22)	0.041(2)	0.030(2)	0.047(3)	-0.0109(19)	0.0026(19)	-0.0008(18)
C(23)	0.034(2)	0.031(2)	0.039(2)	-0.0039(17)	0.0061(17)	0.0045(17)
C(24)	0.0268(19)	0.0282(19)	0.031(2)	0.0015(16)	0.0018(15)	-0.0002(15)
C(25)	0.076(4)	0.041(3)	0.067(4)	-0.005(3)	0.020(3)	-0.009(3)
C(26)	0.037(2)	0.034(2)	0.034(2)	0.0015(17)	0.0050(17)	-0.0011(18)
C(17)	0.031(2)	0.041(2)	0.034(2)	0.0015(18)	0.0078(17)	0.0081(17)
C(16)	0.040(2)	0.050(3)	0.036(2)	0.004(2)	0.0121(18)	0.016(2)

Bond lengths [Å] and angles [°] for **3**^{Se} **- B**.

Se(1)-C(3)	1.890(4)	H(2AB)-C(2)-H(2AC)	109.5
Se(1)-C(1)	1.949(5)	O(5)-S(2)-O(6)	115.2(3)
F(1)-C(26)	1.319(5)	O(5)-S(2)-O(4)	114.4(2)
N(1)-C(3)	1.343(5)	O(6)-S(2)-O(4)	115.8(2)
N(1)-C(7)	1.379(6)	O(5)-S(2)-C(26)	101.0(2)
N(1)-C(4)	1.467(6)	O(6)-S(2)-C(26)	103.8(2)
C(1)-H(1A)	0.9800	O(4)-S(2)-C(26)	104.0(2)
C(1)-H(1AB)	0.9800	C(5)-N(3)-C(18)	109.2(3)
C(1)-H(1AC)	0.9800	C(5)-N(3)-C(24)	125.0(3)
S(1)-O(3)	1.424(4)	C(18)-N(3)-C(24)	125.2(3)
S(1)-O(2)	1.430(3)	N(2)-C(3)-N(1)	108.6(3)
S(1)-O(1)	1.438(4)	N(2)-C(3)-Se(1)	124.8(3)
S(1)-C(25)	1.814(6)	N(1)-C(3)-Se(1)	126.5(3)
Se(2)-C(5)	1.901(4)	C(5)-N(4)-C(13)	109.3(3)
Se(2)-C(2)	1.949(6)	C(5)-N(4)-C(6)	125.7(4)

F(2)-C(26)	1.346(5)	C(13)-N(4)-C(6)	125.0(4)
N(2)-C(3)	1.336(5)	N(1)-C(4)-H(4A)	109.5
N(2)-C(12)	1.395(5)	N(1)-C(4)-H(4AB)	109.5
N(2)-C(20)	1.442(5)	H(4A)-C(4)-H(4AB)	109.5
C(2)-H(2A)	0.9800	N(1)-C(4)-H(4AC)	109.5
C(2)-H(2AB)	0.9800	H(4A)-C(4)-H(4AC)	109.5
C(2)-H(2AC)	0.9800	H(4AB)-C(4)-H(4AC)	109.5
S(2)-O(5)	1.431(4)	N(4)-C(5)-N(3)	108.7(4)
S(2)-O(6)	1.432(4)	N(4)-C(5)-Se(2)	128.8(3)
S(2)-O(4)	1.432(3)	N(3)-C(5)-Se(2)	122.5(3)
S(2)-C(26)	1.807(4)	N(4)-C(6)-H(6A)	109.5
N(3)-C(5)	1.356(5)	N(4)-C(6)-H(6AB)	109.5
N(3)-C(18)	1.396(5)	H(6A)-C(6)-H(6AB)	109.5
N(3)-C(24)	1.440(5)	N(4)-C(6)-H(6AC)	109.5
F(3)-C(26)	1.345(5)	H(6A)-C(6)-H(6AC)	109.5
F(4)-C(25)	1.335(7)	H(6AB)-C(6)-H(6AC)	109.5
N(4)-C(5)	1.326(6)	N(1)-C(7)-C(8)	132.2(4)
N(4)-C(13)	1.396(6)	N(1)-C(7)-C(12)	107.0(4)
N(4)-C(6)	1.468(6)	C(8)-C(7)-C(12)	120.8(4)
C(4)-H(4A)	0.9800	C(8)-C(9)-C(10)	122.5(5)
C(4)-H(4AB)	0.9800	C(8)-C(9)-H(9)	118.7
C(4)-H(4AC)	0.9800	C(10)-C(9)-H(9)	118.7
F(5)-C(25)	1.337(8)	C(9)-C(8)-C(7)	116.4(5)
C(6)-H(6A)	0.9800	C(9)-C(8)-H(8)	121.8
C(6)-H(6AB)	0.9800	C(7)-C(8)-H(8)	121.8
C(6)-H(6AC)	0.9800	C(12)-C(11)-C(10)	114.3(4)
F(6)-C(25)	1.300(8)	C(12)-C(11)-H(11)	122.8
C(7)-C(8)	1.391(6)	C(10)-C(11)-H(11)	122.8
C(7)-C(12)	1.396(6)	C(9)-C(10)-C(11)	121.9(5)
C(9)-C(8)	1.379(8)	C(9)-C(10)-H(10)	119.1
C(9)-C(10)	1.393(8)	C(11)-C(10)-H(10)	119.1
C(9)-H(9)	0.9500	C(14)-C(15)-C(16)	122.3(4)
C(8)-H(8)	0.9500	C(14)-C(15)-H(15)	118.8
C(11)-C(12)	1.383(6)	C(16)-C(15)-H(15)	118.8
C(11)-C(10)	1.410(7)	C(15)-C(14)-C(13)	116.3(4)
C(11)-H(11)	0.9500	C(15)-C(14)-H(14)	121.9
C(10)-H(10)	0.9500	C(13)-C(14)-H(14)	121.9

C(15)-C(14)	1.362(7)	C(18)-C(13)-C(14)	121.2(4)
C(15)-C(16)	1.398(8)	C(18)-C(13)-N(4)	107.0(4)
C(15)-H(15)	0.9500	C(14)-C(13)-N(4)	131.7(4)
C(14)-C(13)	1.392(6)	C(11)-C(12)-N(2)	130.4(4)
C(14)-H(14)	0.9500	C(11)-C(12)-C(7)	123.9(4)
C(13)-C(18)	1.391(6)	N(2)-C(12)-C(7)	105.6(4)
C(18)-C(17)	1.379(6)	C(17)-C(18)-C(13)	122.7(4)
C(19)-C(20)	1.381(6)	C(17)-C(18)-N(3)	131.5(4)
C(19)-C(24)	1.391(6)	C(13)-C(18)-N(3)	105.8(4)
C(19)-H(19)	0.9500	C(20)-C(19)-C(24)	117.5(4)
C(20)-C(21)	1.385(6)	C(20)-C(19)-H(19)	121.2
C(21)-C(22)	1.383(6)	C(24)-C(19)-H(19)	121.2
C(21)-H(21)	0.9500	C(19)-C(20)-C(21)	121.8(4)
C(22)-C(23)	1.383(6)	C(19)-C(20)-N(2)	117.9(3)
C(22)-H(22)	0.9500	C(21)-C(20)-N(2)	120.2(4)
C(23)-C(24)	1.377(6)	C(22)-C(21)-C(20)	119.0(4)
C(23)-H(23)	0.9500	C(22)-C(21)-H(21)	120.5
C(17)-C(16)	1.382(6)	C(20)-C(21)-H(21)	120.5
C(17)-H(17)	0.9500	C(21)-C(22)-C(23)	120.7(4)
C(16)-H(16)	0.9500	C(21)-C(22)-H(22)	119.7
C(3)-Se(1)-C(1)	93.1(2)	C(23)-C(22)-H(22)	119.7
C(3)-N(1)-C(7)	109.2(3)	C(24)-C(23)-C(22)	118.9(4)
C(3)-N(1)-C(4)	126.7(4)	C(24)-C(23)-H(23)	120.5
C(7)-N(1)-C(4)	124.1(4)	C(22)-C(23)-H(23)	120.5
Se(1)-C(1)-H(1A)	109.5	C(23)-C(24)-C(19)	122.0(4)
Se(1)-C(1)-H(1AB)	109.5	C(23)-C(24)-N(3)	120.5(4)
H(1A)-C(1)-H(1AB)	109.5	C(19)-C(24)-N(3)	117.4(3)
Se(1)-C(1)-H(1AC)	109.5	F(6)-C(25)-F(4)	106.2(5)
H(1A)-C(1)-H(1AC)	109.5	F(6)-C(25)-F(5)	110.4(6)
H(1AB)-C(1)-H(1AC)	109.5	F(4)-C(25)-F(5)	104.8(5)
O(3)-S(1)-O(2)	115.1(2)	F(6)-C(25)-S(1)	112.6(5)
O(3)-S(1)-O(1)	113.7(3)	F(4)-C(25)-S(1)	112.0(4)
O(2)-S(1)-O(1)	115.1(2)	F(5)-C(25)-S(1)	110.5(4)
O(3)-S(1)-C(25)	104.3(3)	F(1)-C(26)-F(3)	107.7(4)
O(2)-S(1)-C(25)	103.8(2)	F(1)-C(26)-F(2)	106.9(4)
O(1)-S(1)-C(25)	102.5(3)	F(3)-C(26)-F(2)	106.1(4)
C(5)-Se(2)-C(2)	98.9(2)	F(1)-C(26)-S(2)	113.0(3)

C(3)-N(2)-C(12)	109.5(3)	F(3)-C(26)-S(2)	110.9(3)
C(3)-N(2)-C(20)	125.9(3)	F(2)-C(26)-S(2)	111.9(3)
C(12)-N(2)-C(20)	124.1(3)	C(18)-C(17)-C(16)	115.5(4)
Se(2)-C(2)-H(2A)	109.5	C(18)-C(17)-H(17)	122.2
Se(2)-C(2)-H(2AB)	109.5	C(16)-C(17)-H(17)	122.2
H(2A)-C(2)-H(2AB)	109.5	C(17)-C(16)-C(15)	121.9(5)
Se(2)-C(2)-H(2AC)	109.5	C(17)-C(16)-H(16)	119.0
H(2A)-C(2)-H(2AC)	109.5	C(15)-C(16)-H(16)	119.0

Symmetry transformations used to generate equivalent atoms.

S1.10. 3Se - C

S1.10.1. Crystal Data 3^{Se} - C.

$C_{27}H_{26}ClF_6N_4O_6S_2Se_2$
M_r = 874.01 g/mol
Triclinic P-1
a = 9.3672(1) Å
b = 13.3265(2) Å
c = 13.6445(1) Å
α = 102.515 (1),
β = 93.231 (1),
γ = 96.198 (1)
V = 1647.49 (3) $Å^3$
z = 4

F(000) = 870
D_x = 1.762 g/cm^3
Cu K_α radiation, λ = 1.54184 Å
Cell parameters from 5795 reflections
θ = 3.3 - 66.5°
μ = 5.48 mm^{-1}
T = 169.99 K
Cut plate, translucent colourless
0.19 x 0.10 x 0.08 mm

S1.11. Data collection 3^{Se} - C.

XtaLAB Synergy, Dualflex, HyPix
Radiation source: PhotonJet (Cu) X-ray Source
Mirror monochromator
ω scans
Absorption correction: Gaussian, CrysAlis PRO 1.171.40.20a (Rigaku Oxford Diffraction, 2018) Numerical absorption correction based on gaussian integration over a multifaceted crystal model Empirical absorption correction using spherical harmonics, implemented in SCALE3 ABSPACK scaling algorithm.

Tmin = 0.456, Tmax = 1.000
19605 measured reflections
5795 independent reflections
5307 reflections with I > 2σ(I)
R_{int} = 4.6%
θ_{max} = 66.5°, θ_{min} = 3.3°
h = -11 → 10
k = -15 → 15
l = -14 → 16

S1.12. Refinement 3^{Se}- C.

Refinement on F2
Least-squares matrix: full

R[F2 > 2σ(F2)] = 4.8%
wR(F2) = 12.7%

S = 1.05
5795 reflections
484 parameters
28 restraints

$\Delta\rho_{max}$, $\Delta\rho_{min}$ (e Å^{-3}) = 0.84, −0.79
Primary atom site location: dual
Hydrogen site location: constrained

The crystal structure of **3**^{Se} in 1,2-dichloroethan (structure C) contains a 95:5 disorder of one benzimidazolium unit (Figure S1). However, only the interactions of the 95 % site play a role for the investigated interactions.

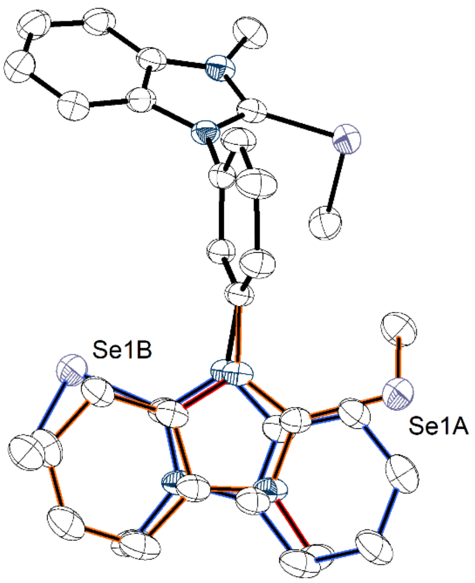

Figure S1 Graphic of the 95:5 disorder in structure C of **3**^{Se}.

Table S4 **3**^{Se} **- C**

Atomic coordinates (x 10^4) and equivalent isotropic displacement parameters (Å^{2}x 10^3) for **3**^{Se} **- C**. U(eq) is defined as one third of the trace of the orthogonalised U^{ij} tensor.

	x	y	z	U(eq)
Se(1A)	0.51229(5)	0.78078(3)	0.20663(3)	0.03655(15)
N(1A)	0.3629(6)	0.7664(3)	0.3822(3)	0.0317(8)
N(2A)	0.4146(11)	0.6162(3)	0.3040(4)	0.0261(7)
C(1A)	0.7037(5)	0.7379(4)	0.2166(3)	0.0420(10)
C(3A)	0.4277(7)	0.7173(3)	0.3041(3)	0.0282(10)
C(4A)	0.3515(9)	0.8774(4)	0.4098(6)	0.0499(17)
C(5A)	0.3027(4)	0.6943(3)	0.4323(3)	0.0315(8)
C(6A)	0.2161(5)	0.7041(4)	0.5138(3)	0.0423(11)
C(7A)	0.1696(5)	0.6134(4)	0.5419(3)	0.0455(11)

C(8A)	0.2087(5)	0.5180(4)	0.4944(5)	0.0421(12)
C(9A)	0.2924(4)	0.5074(3)	0.4139(3)	0.0341(9)
C(10A)	0.3373(5)	0.5987(4)	0.3846(3)	0.0283(11)
Se(1B)	0.3520(10)	0.4679(6)	0.4492(6)	0.03655(15)
C(1B)	0.170(9)	0.503(11)	0.499(13)	0.0420(10)
N(1B)	0.314376	0.678949	0.447267	0.0317(8)
N(2B)	0.41(2)	0.604(4)	0.312(8)	0.0261(7)
C(3B)	0.359(15)	0.590(4)	0.400(5)	0.0282(10)
C(4B)	0.262(13)	0.702(6)	0.547(4)	0.0499(17)
C(5B)	0.341(16)	0.762(5)	0.403(8)	0.0315(8)
C(6B)	0.322(19)	0.867(6)	0.417(9)	0.0423(11)
C(7B)	0.366(9)	0.912(4)	0.340(5)	0.0455(11)
C(8B)	0.446(8)	0.864(4)	0.264(4)	0.0421(12)
C(9B)	0.474(8)	0.763(4)	0.257(5)	0.0341(9)
C(10B)	0.413(16)	0.712(4)	0.325(8)	0.0283(11)
C(2A)	0.9577(5)	0.5809(3)	0.2775(3)	0.0440(10)
N(3)	0.7211(3)	0.3466(2)	0.1936(2)	0.0279(6)
N(4)	0.9361(3)	0.2978(2)	0.1916(2)	0.0311(6)
Se(2)	0.92896(4)	0.49697(3)	0.13920(3)	0.03528(14)
C(11)	0.8597(4)	0.3727(3)	0.1769(3)	0.0293(7)
C(12)	1.0894(4)	0.2924(4)	0.1769(3)	0.0410(9)
C(13)	0.8453(4)	0.2191(3)	0.2166(3)	0.0312(8)
C(14)	0.8724(5)	0.1257(3)	0.2395(3)	0.0389(9)
C(15)	0.7561(5)	0.0660(3)	0.2636(3)	0.0443(10)
C(16)	0.6181(5)	0.0981(3)	0.2668(3)	0.0445(10)
C(17)	0.5906(5)	0.1913(3)	0.2439(3)	0.0384(9)
C(18)	0.7082(4)	0.2503(3)	0.2177(3)	0.0310(8)
C(19)	0.4537(4)	0.5337(3)	0.2273(2)	0.0260(7)
C(20)	0.3729(4)	0.5062(3)	0.1355(3)	0.0321(8)
C(21)	0.4075(5)	0.4237(3)	0.0631(3)	0.0385(9)
C(22)	0.5228(4)	0.3717(3)	0.0820(3)	0.0332(8)
C(23)	0.6021(4)	0.4021(3)	0.1748(3)	0.0279(7)
C(24)	0.5678(4)	0.4821(3)	0.2498(3)	0.0270(7)
S(1)	0.11819(10)	0.75468(7)	0.09579(7)	0.0331(2)
F(1)	0.2088(4)	0.9489(3)	0.1760(3)	0.0861(12)

F(2)	-0.0039(4)	0.8998(3)	0.2082(3)	0.0947(14)
F(3)	0.0356(5)	0.9246(3)	0.0616(4)	0.1094(15)
O(1)	0.1765(4)	0.7316(3)	0.1871(2)	0.0558(8)
O(2)	0.2189(3)	0.7585(3)	0.0206(2)	0.0495(8)
O(3)	-0.0227(4)	0.7008(3)	0.0599(2)	0.0528(8)
C(25)	0.0879(6)	0.8895(4)	0.1370(5)	0.0594(13)
F(4)	0.0437(4)	0.1670(4)	0.5413(5)	0.126(2)
F(5)	0.2090(5)	0.0786(3)	0.5591(3)	0.0908(12)
F(6)	0.0937(4)	0.0531(2)	0.4146(3)	0.0793(10)
C(26)	0.1483(5)	0.1260(4)	0.4950(4)	0.0552(12)
Cl(1A)	0.352(2)	0.1088(13)	-0.0121(18)	0.062(2)
C(27A)	0.5136(10)	0.0490(7)	-0.0184(7)	0.0509(18)
Cl(1B)	0.345(3)	0.1106(19)	0.003(2)	0.067(5)
C(27B)	0.4228(14)	-0.0051(10)	0.0141(9)	0.0509(18)
S(2A)	0.2594(6)	0.2311(5)	0.4639(5)	0.0436(9)
O(4A)	0.3166(4)	0.2966(3)	0.5527(3)	0.0635(7)
O(5A)	0.1604(10)	0.2659(6)	0.4004(6)	0.0635(7)
O(6A)	0.3731(9)	0.1796(7)	0.4160(6)	0.0635(7)
S(2B)	0.2967(6)	0.2109(5)	0.4596(5)	0.0436(9)
O(4B)	0.3166(4)	0.2966(3)	0.5527(3)	0.0635(7)
O(5B)	0.2447(9)	0.2429(6)	0.3721(5)	0.0635(7)
O(6B)	0.4035(8)	0.1392(6)	0.4430(6)	0.0635(7)
H(1AA)	0.764582	0.768208	0.171733	0.063
H(1AB)	0.696569	0.662222	0.196995	0.063
H(1AC)	0.746273	0.761771	0.286196	0.063
H(4AA)	0.360353	0.900770	0.483322	0.075
H(4AB)	0.257903	0.890591	0.382867	0.075
H(4AC)	0.428760	0.915132	0.381636	0.075
H(6A)	0.190937	0.769239	0.547424	0.051
H(7A)	0.108846	0.616114	0.595601	0.055
H(8A)	0.176484	0.458318	0.518278	0.051
H(9A)	0.317749	0.442275	0.380633	0.041
H(1BA)	0.154002	0.571836	0.488350	0.063
H(1BB)	0.171045	0.503692	0.570562	0.063
H(1BC)	0.091254	0.451706	0.461725	0.063

H(4BA)	0.267257	0.777660	0.571149	0.075
H(4BB)	0.320596	0.674078	0.593420	0.075
H(4BC)	0.161140	0.671305	0.543359	0.075
H(6B)	0.282495	0.904160	0.474158	0.051
H(7B)	0.340495	0.978647	0.338420	0.055
H(8B)	0.482276	0.901547	0.216517	0.051
H(9B)	0.532521	0.731073	0.207871	0.041
H(2AA)	1.000622	0.651134	0.276882	0.066
H(2AB)	1.022288	0.549576	0.317907	0.066
H(2AC)	0.864711	0.583860	0.306753	0.066
H(12A)	1.139658	0.284118	0.239078	0.062
H(12B)	1.131541	0.356278	0.159712	0.062
H(12C)	1.099365	0.233066	0.122044	0.062
H(14)	0.965944	0.104311	0.238527	0.047
H(15)	0.769564	0.001151	0.278621	0.053
H(16)	0.541410	0.054914	0.285092	0.053
H(17)	0.497432	0.213314	0.245928	0.046
H(20)	0.295222	0.542871	0.122199	0.039
H(21)	0.351792	0.402888	0.000176	0.046
H(22)	0.547201	0.315945	0.032132	0.040
H(24)	0.620428	0.500739	0.313983	0.032
H(27A)	0.540977	0.033300	-0.088571	0.061
H(27B)	0.593142	0.096079	0.024336	0.061
H(27C)	0.420525	-0.014172	0.084101	0.061
H(27D)	0.366556	-0.066503	-0.031233	0.061

Anisotropic displacement parameters [Å^2] for **3^{Se}- C**.

	U11	U22	U33	U23	U13	U12
Se(1A)	0.0403(3)	0.0340(2)	0.0387(3)	0.01247(18)	0.00641(19)	0.00902(18)
N(1A)	0.039(3)	0.0313(17)	0.024(2)	-0.0016(14)	0.0022(16)	0.0121(15)
N(2A)	0.0257(19)	0.0271(16)	0.0244(18)	0.0012(13)	0.0029(13)	0.007(2)
C(1A)	0.034(2)	0.052(3)	0.041(2)	0.010(2)	0.0057(18)	0.0068(19)
C(3A)	0.031(2)	0.0296(19)	0.023(3)	0.0020(15)	-0.0001(19)	0.0095(16)

C(4A)	0.072(6)	0.033(2)	0.045(3)	0.000(2)	0.013(3)	0.020(3)
C(5A)	0.036(2)	0.036(2)	0.0205(17)	0.0009(15)	-0.0002(15)	0.0099(16)
C(6A)	0.047(3)	0.052(3)	0.0261(19)	-0.0009(19)	0.0065(18)	0.018(2)
C(7A)	0.052(3)	0.059(3)	0.026(2)	0.0076(19)	0.0114(18)	0.011(2)
C(8A)	0.042(3)	0.056(3)	0.030(2)	0.014(2)	0.005(3)	0.003(3)
C(9A)	0.035(2)	0.043(2)	0.0261(18)	0.0080(17)	0.0059(16)	0.0103(18)
C(10A)	0.026(3)	0.035(2)	0.0219(19)	0.0020(15)	-0.0016(18)	0.0076(15)
Se(1B)	0.0403(3)	0.0340(2)	0.0387(3)	0.01247(18)	0.00641(19)	0.00902(18)
C(1B)	0.034(2)	0.052(3)	0.041(2)	0.010(2)	0.0057(18)	0.0068(19)
N(1B)	0.039(3)	0.0313(17)	0.024(2)	-0.0016(14)	0.0022(16)	0.0121(15)
N(2B)	0.0257(19)	0.0271(16)	0.0244(18)	0.0012(13)	0.0029(13)	0.007(2)
C(3B)	0.031(2)	0.0296(19)	0.023(3)	0.0020(15)	-0.0001(19)	0.0095(16)
C(4B)	0.072(6)	0.033(2)	0.045(3)	0.000(2)	0.013(3)	0.020(3)
C(5B)	0.036(2)	0.036(2)	0.0205(17)	0.0009(15)	-0.0002(15)	0.0099(16)
C(6B)	0.047(3)	0.052(3)	0.0261(19)	-0.0009(19)	0.0065(18)	0.018(2)
C(7B)	0.052(3)	0.059(3)	0.026(2)	0.0076(19)	0.0114(18)	0.011(2)
C(8B)	0.042(3)	0.056(3)	0.030(2)	0.014(2)	0.005(3)	0.003(3)
C(9B)	0.035(2)	0.043(2)	0.0261(18)	0.0080(17)	0.0059(16)	0.0103(18)
C(10B)	0.026(3)	0.035(2)	0.0219(19)	0.0020(15)	-0.0016(18)	0.0076(15)
C(2A)	0.046(2)	0.042(2)	0.041(2)	0.0067(18)	-0.0002(18)	-0.0025(18)
N(3)	0.0314(15)	0.0262(14)	0.0275(14)	0.0047(12)	0.0057(12)	0.0103(12)
N(4)	0.0321(16)	0.0347(16)	0.0270(15)	0.0047(12)	0.0031(12)	0.0106(13)
Se(2)	0.0374(2)	0.0349(2)	0.0354(2)	0.01026(17)	0.00790(17)	0.00516(17)
C(11)	0.0319(19)	0.0328(18)	0.0234(16)	0.0034(14)	0.0035(14)	0.0108(15)
C(12)	0.031(2)	0.056(2)	0.040(2)	0.0129(19)	0.0052(16)	0.0166(18)
C(13)	0.038(2)	0.0320(18)	0.0228(16)	0.0021(14)	0.0053(14)	0.0098(15)
C(14)	0.052(2)	0.035(2)	0.0325(19)	0.0075(16)	0.0041(17)	0.0177(18)
C(15)	0.063(3)	0.030(2)	0.042(2)	0.0083(17)	0.005(2)	0.0132(19)
C(16)	0.059(3)	0.033(2)	0.043(2)	0.0096(17)	0.010(2)	0.0037(19)
C(17)	0.041(2)	0.036(2)	0.039(2)	0.0066(17)	0.0107(17)	0.0075(17)

C(18)	0.037(2)	0.0283(18)	0.0274(17)	0.0035(14)	0.0043(15)	0.0098(15)
C(19)	0.0274(17)	0.0256(17)	0.0240(16)	0.0016(13)	0.0058(13)	0.0052(13)
C(20)	0.0301(18)	0.0360(19)	0.0307(18)	0.0051(15)	0.0012(14)	0.0115(15)
C(21)	0.043(2)	0.045(2)	0.0257(18)	-0.0003(16)	-0.0031(15)	0.0138(18)
C(22)	0.042(2)	0.0320(18)	0.0233(17)	-0.0009(14)	0.0033(15)	0.0101(16)
C(23)	0.0307(18)	0.0273(17)	0.0278(17)	0.0072(14)	0.0079(14)	0.0083(14)
C(24)	0.0299(17)	0.0265(17)	0.0238(16)	0.0030(13)	0.0028(13)	0.0056(14)
S(1)	0.0357(5)	0.0350(5)	0.0303(4)	0.0064(4)	0.0068(4)	0.0125(4)
F(1)	0.083(2)	0.0558(18)	0.100(3)	-0.0168(18)	0.032(2)	-0.0195(17)
F(2)	0.080(2)	0.0578(19)	0.137(3)	-0.015(2)	0.065(2)	0.0152(17)
F(3)	0.117(3)	0.078(3)	0.158(4)	0.069(3)	0.002(3)	0.041(2)
O(1)	0.0515(19)	0.080(2)	0.0465(17)	0.0311(17)	0.0052(14)	0.0198(17)
O(2)	0.0509(18)	0.064(2)	0.0327(15)	0.0042(14)	0.0140(13)	0.0124(15)
O(3)	0.0496(18)	0.0523(18)	0.0515(18)	0.0069(15)	-0.0018(14)	-0.0028(15)
C(25)	0.054(3)	0.046(3)	0.080(4)	0.012(3)	0.021(3)	0.009(2)
F(4)	0.065(2)	0.098(3)	0.198(5)	-0.012(3)	0.051(3)	0.003(2)
F(5)	0.149(4)	0.062(2)	0.063(2)	0.0237(16)	-0.008(2)	0.008(2)
F(6)	0.092(2)	0.0539(18)	0.079(2)	0.0038(16)	-0.0269(19)	-0.0090(17)
C(26)	0.050(3)	0.042(2)	0.067(3)	-0.003(2)	-0.004(2)	0.011(2)
Cl(1A)	0.072(5)	0.056(3)	0.057(3)	0.016(2)	0.008(3)	-0.002(3)
C(27A)	0.054(5)	0.048(5)	0.048(3)	0.009(3)	0.008(4)	-0.006(3)
Cl(1B)	0.061(4)	0.063(5)	0.078(12)	0.015(5)	0.013(5)	0.016(4)
C(27B)	0.054(5)	0.048(5)	0.048(3)	0.009(3)	0.008(4)	-0.006(3)
S(2A)	0.044(3)	0.053(2)	0.0337(7)	0.0100(13)	0.0019(15)	0.0048(14)
O(4A)	0.0682(17)	0.0675(17)	0.0527(15)	0.0189(12)	-0.0107(12)	-0.0037(14)
O(5A)	0.0682(17)	0.0675(17)	0.0527(15)	0.0189(12)	-0.0107(12)	-0.0037(14)
O(6A)	0.0682(17)	0.0675(17)	0.0527(15)	0.0189(12)	-0.0107(12)	-0.0037(14)
S(2B)	0.044(3)	0.053(2)	0.0337(7)	0.0100(13)	0.0019(15)	0.0048(14)
O(4B)	0.0682(17)	0.0675(17)	0.0527(15)	0.0189(12)	-0.0107(12)	-0.0037(14)
O(5B)	0.0682(17)	0.0675(17)	0.0527(15)	0.0189(12)	-0.0107(12)	-0.0037(14)

O(6B)	0.0682(17)	0.0675(17)	0.0527(15)	0.0189(12)	-0.0107(12)	-0.0037(14)

Bond lengths [Å] and angles [°] for **3**^{Se} **- C**.

Se(1A)-C(3A)	1.891(4)	C(9A)-C(10A)-N(2A)	131.0(4)
Se(1A)-C(1A)	1.946(4)	C(3B)-Se(1B)-C(1B)	86(5)
N(1A)-C(3A)	1.338(5)	Se(1B)-C(1B)-H(1BA)	109.5
N(1A)-C(5A)	1.386(5)	Se(1B)-C(1B)-H(1BB)	109.5
N(1A)-C(4A)	1.461(5)	H(1BA)-C(1B)-H(1BB)	109.5
N(2A)-C(3A)	1.339(5)	Se(1B)-C(1B)-H(1BC)	109.5
N(2A)-C(10A)	1.397(6)	H(1BA)-C(1B)-H(1BC)	109.5
N(2A)-C(19)	1.439(4)	H(1BB)-C(1B)-H(1BC)	109.5
C(1A)-H(1AA)	0.9800	C(3B)-N(1B)-C(5B)	116(4)
C(1A)-H(1AB)	0.9800	C(3B)-N(1B)-C(4B)	128(3)
C(1A)-H(1AC)	0.9800	C(5B)-N(1B)-C(4B)	116(4)
C(4A)-H(4AA)	0.9800	C(3B)-N(2B)-C(10B)	102(4)
C(4A)-H(4AB)	0.9800	C(3B)-N(2B)-C(19)	132(3)
C(4A)-H(4AC)	0.9800	C(10B)-N(2B)-C(19)	126(4)
C(5A)-C(10A)	1.382(6)	N(1B)-C(3B)-N(2B)	108.1(18)
C(5A)-C(6A)	1.404(6)	N(1B)-C(3B)-Se(1B)	125.1(18)
C(6A)-C(7A)	1.380(7)	N(2B)-C(3B)-Se(1B)	127(3)
C(6A)-H(6A)	0.9500	N(1B)-C(4B)-H(4BA)	109.5
C(7A)-C(8A)	1.393(8)	N(1B)-C(4B)-H(4BB)	109.5
C(7A)-H(7A)	0.9500	H(4BA)-C(4B)-H(4BB)	109.5
C(8A)-C(9A)	1.376(7)	N(1B)-C(4B)-H(4BC)	109.5
C(8A)-H(8A)	0.9500	H(4BA)-C(4B)-H(4BC)	109.5
C(9A)-C(10A)	1.391(7)	H(4BB)-C(4B)-H(4BC)	109.5
C(9A)-H(9A)	0.9500	N(1B)-C(5B)-C(10B)	97(5)
Se(1B)-C(3B)	1.89(2)	N(1B)-C(5B)-C(6B)	141(6)
Se(1B)-C(1B)	1.94(2)	C(10B)-C(5B)-C(6B)	122(2)
C(1B)-H(1BA)	0.9800	C(7B)-C(6B)-C(5B)	114(3)
C(1B)-H(1BB)	0.9800	C(7B)-C(6B)-H(6B)	122.9
C(1B)-H(1BC)	0.9800	C(5B)-C(6B)-H(6B)	122.9
N(1B)-C(3B)	1.337(19)	C(6B)-C(7B)-C(8B)	123(3)
N(1B)-C(5B)	1.38(2)	C(6B)-C(7B)-H(7B)	118.3
N(1B)-C(4B)	1.455(18)	C(8B)-C(7B)-H(7B)	118.3

N(2B)-C(3B)	1.35(2)	C(9B)-C(8B)-C(7B)	120(2)
N(2B)-C(10B)	1.40(2)	C(9B)-C(8B)-H(8B)	119.8
N(2B)-C(19)	1.446(16)	C(7B)-C(8B)-H(8B)	119.8
C(4B)-H(4BA)	0.9800	C(10B)-C(9B)-C(8B)	117(2)
C(4B)-H(4BB)	0.9800	C(10B)-C(9B)-H(9B)	121.4
C(4B)-H(4BC)	0.9800	C(8B)-C(9B)-H(9B)	121.4
C(5B)-C(10B)	1.38(2)	C(9B)-C(10B)-C(5B)	121(3)
C(5B)-C(6B)	1.41(2)	C(9B)-C(10B)-N(2B)	122(6)
C(6B)-C(7B)	1.37(2)	C(5B)-C(10B)-N(2B)	116(6)
C(6B)-H(6B)	0.9500	Se(2)-C(2A)-H(2AA)	109.5
C(7B)-C(8B)	1.40(2)	Se(2)-C(2A)-H(2AB)	109.5
C(7B)-H(7B)	0.9500	H(2AA)-C(2A)-H(2AB)	109.5
C(8B)-C(9B)	1.38(2)	Se(2)-C(2A)-H(2AC)	109.5
C(8B)-H(8B)	0.9500	H(2AA)-C(2A)-H(2AC)	109.5
C(9B)-C(10B)	1.38(2)	H(2AB)-C(2A)-H(2AC)	109.5
C(9B)-H(9B)	0.9500	C(11)-N(3)-C(18)	108.9(3)
C(2A)-Se(2)	1.961(4)	C(11)-N(3)-C(23)	125.6(3)
C(2A)-H(2AA)	0.9800	C(18)-N(3)-C(23)	125.0(3)
C(2A)-H(2AB)	0.9800	C(11)-N(4)-C(13)	109.2(3)
C(2A)-H(2AC)	0.9800	C(11)-N(4)-C(12)	126.8(3)
N(3)-C(11)	1.352(5)	C(13)-N(4)-C(12)	123.8(3)
N(3)-C(18)	1.388(5)	C(11)-Se(2)-C(2A)	94.86(17)
N(3)-C(23)	1.444(5)	N(4)-C(11)-N(3)	108.8(3)
N(4)-C(11)	1.331(5)	N(4)-C(11)-Se(2)	126.9(3)
N(4)-C(13)	1.391(5)	N(3)-C(11)-Se(2)	124.2(3)
N(4)-C(12)	1.468(5)	N(4)-C(12)-H(12A)	109.5
Se(2)-C(11)	1.899(4)	N(4)-C(12)-H(12B)	109.5
C(12)-H(12A)	0.9800	H(12A)-C(12)-H(12B)	109.5
C(12)-H(12B)	0.9800	N(4)-C(12)-H(12C)	109.5
C(12)-H(12C)	0.9800	H(12A)-C(12)-H(12C)	109.5
C(13)-C(18)	1.392(5)	H(12B)-C(12)-H(12C)	109.5
C(13)-C(14)	1.393(5)	N(4)-C(13)-C(18)	106.6(3)
C(14)-C(15)	1.376(7)	N(4)-C(13)-C(14)	131.5(4)
C(14)-H(14)	0.9500	C(18)-C(13)-C(14)	121.9(4)
C(15)-C(16)	1.405(7)	C(15)-C(14)-C(13)	116.3(4)
C(15)-H(15)	0.9500	C(15)-C(14)-H(14)	121.9

C(16)-C(17)	1.391(6)	C(13)-C(14)-H(14)	121.9
C(16)-H(16)	0.9500	C(14)-C(15)-C(16)	122.1(4)
C(17)-C(18)	1.392(6)	C(14)-C(15)-H(15)	119.0
C(17)-H(17)	0.9500	C(16)-C(15)-H(15)	119.0
C(19)-C(20)	1.382(5)	C(17)-C(16)-C(15)	121.8(4)
C(19)-C(24)	1.386(5)	C(17)-C(16)-H(16)	119.1
C(20)-C(21)	1.389(5)	C(15)-C(16)-H(16)	119.1
C(20)-H(20)	0.9500	C(16)-C(17)-C(18)	115.7(4)
C(21)-C(22)	1.386(6)	C(16)-C(17)-H(17)	122.1
C(21)-H(21)	0.9500	C(18)-C(17)-H(17)	122.1
C(22)-C(23)	1.387(5)	N(3)-C(18)-C(13)	106.5(3)
C(22)-H(22)	0.9500	N(3)-C(18)-C(17)	131.3(3)
C(23)-C(24)	1.387(5)	C(13)-C(18)-C(17)	122.2(3)
C(24)-H(24)	0.9500	C(20)-C(19)-C(24)	122.5(3)
S(1)-O(2)	1.437(3)	C(20)-C(19)-N(2A)	118.9(5)
S(1)-O(3)	1.437(3)	C(24)-C(19)-N(2A)	118.6(5)
S(1)-O(1)	1.439(3)	C(20)-C(19)-N(2B)	121(9)
S(1)-C(25)	1.821(5)	C(24)-C(19)-N(2B)	115(8)
F(1)-C(25)	1.323(6)	C(19)-C(20)-C(21)	118.7(3)
F(2)-C(25)	1.328(6)	C(19)-C(20)-H(20)	120.7
F(3)-C(25)	1.310(7)	C(21)-C(20)-H(20)	120.7
F(4)-C(26)	1.301(6)	C(22)-C(21)-C(20)	120.5(3)
F(5)-C(26)	1.322(6)	C(22)-C(21)-H(21)	119.7
F(6)-C(26)	1.333(6)	C(20)-C(21)-H(21)	119.7
C(26)-S(2A)	1.794(9)	C(21)-C(22)-C(23)	119.1(3)
C(26)-S(2B)	1.850(8)	C(21)-C(22)-H(22)	120.5
Cl(1A)-C(27A)	1.78(2)	C(23)-C(22)-H(22)	120.5
C(27A)-C(27A)#1	1.499(18)	C(24)-C(23)-C(22)	121.9(3)
C(27A)-H(27A)	0.9900	C(24)-C(23)-N(3)	119.7(3)
C(27A)-H(27B)	0.9900	C(22)-C(23)-N(3)	118.4(3)
Cl(1B)-C(27B)	1.81(3)	C(19)-C(24)-C(23)	117.3(3)
C(27B)-C(27B)#1	1.52(3)	C(19)-C(24)-H(24)	121.3
C(27B)-H(27C)	0.9900	C(23)-C(24)-H(24)	121.3
C(27B)-H(27D)	0.9900	O(2)-S(1)-O(3)	115.94(19)
S(2A)-O(4A)	1.372(8)	O(2)-S(1)-O(1)	114.8(2)
S(2A)-O(5A)	1.415(8)	O(3)-S(1)-O(1)	114.0(2)
S(2A)-O(6A)	1.441(9)	O(2)-S(1)-C(25)	103.0(2)

S(2B)-O(5B)	1.430(8)	O(3)-S(1)-C(25)	104.2(2)
S(2B)-O(6B)	1.451(8)	O(1)-S(1)-C(25)	102.5(3)
S(2B)-O(4B)	1.500(7)	F(3)-C(25)-F(1)	108.3(5)
C(3A)-Se(1A)-C(1A)	99.1(2)	F(3)-C(25)-F(2)	108.6(5)
C(3A)-N(1A)-C(5A)	108.9(3)	F(1)-C(25)-F(2)	106.9(5)
C(3A)-N(1A)-C(4A)	126.5(4)	F(3)-C(25)-S(1)	110.9(4)
C(5A)-N(1A)-C(4A)	124.6(3)	F(1)-C(25)-S(1)	111.3(4)
C(3A)-N(2A)-C(10A)	109.4(3)	F(2)-C(25)-S(1)	110.7(4)
C(3A)-N(2A)-C(19)	127.2(3)	F(4)-C(26)-F(5)	104.9(5)
C(10A)-N(2A)-C(19)	122.9(4)	F(4)-C(26)-F(6)	109.4(5)
Se(1A)-C(1A)-H(1AA)	109.5	F(5)-C(26)-F(6)	107.1(4)
Se(1A)-C(1A)-H(1AB)	109.5	F(4)-C(26)-S(2A)	105.8(4)
H(1AA)-C(1A)-H(1AB)	109.5	F(5)-C(26)-S(2A)	116.0(4)
Se(1A)-C(1A)-H(1AC)	109.5	F(6)-C(26)-S(2A)	113.2(5)
H(1AA)-C(1A)-H(1AC)	109.5	F(4)-C(26)-S(2B)	119.5(4)
H(1AB)-C(1A)-H(1AC)	109.5	F(5)-C(26)-S(2B)	105.5(4)
N(1A)-C(3A)-N(2A)	108.6(3)	F(6)-C(26)-S(2B)	109.7(4)
N(1A)-C(3A)-Se(1A)	124.8(3)	C(27A)#1-C(27A)-Cl(1A)	107.9(10)
N(2A)-C(3A)-Se(1A)	126.5(3)	C(27A)#1-C(27A)-H(27A)	110.1
N(1A)-C(4A)-H(4AA)	109.5	Cl(1A)-C(27A)-H(27A)	110.1
N(1A)-C(4A)-H(4AB)	109.5	C(27A)#1-C(27A)-H(27B)	110.1
H(4AA)-C(4A)-H(4AB)	109.5	Cl(1A)-C(27A)-H(27B)	110.1
N(1A)-C(4A)-H(4AC)	109.5	H(27A)-C(27A)-H(27B)	108.4
H(4AA)-C(4A)-H(4AC)	109.5	C(27B)#1-C(27B)-Cl(1B)	108.7(15)
H(4AB)-C(4A)-H(4AC)	109.5	C(27B)#1-C(27B)-H(27C)	110.0
C(10A)-C(5A)-N(1A)	107.4(4)	Cl(1B)-C(27B)-H(27C)	110.0
C(10A)-C(5A)-C(6A)	120.9(4)	C(27B)#1-C(27B)-H(27D)	110.0
N(1A)-C(5A)-C(6A)	131.7(4)	Cl(1B)-C(27B)-H(27D)	110.0
C(7A)-C(6A)-C(5A)	115.9(4)	H(27C)-C(27B)-H(27D)	108.3
C(7A)-C(6A)-H(6A)	122.0	O(4A)-S(2A)-O(5A)	119.3(5)
C(5A)-C(6A)-H(6A)	122.0	O(4A)-S(2A)-O(6A)	108.6(5)
C(6A)-C(7A)-C(8A)	122.4(4)	O(5A)-S(2A)-O(6A)	116.4(7)

C(6A)-C(7A)-H(7A)	118.8	O(4A)-S(2A)-C(26)	107.6(5)
C(8A)-C(7A)-H(7A)	118.8	O(5A)-S(2A)-C(26)	100.8(5)
C(9A)-C(8A)-C(7A)	122.2(5)	O(6A)-S(2A)-C(26)	102.0(5)
C(9A)-C(8A)-H(8A)	118.9	O(5B)-S(2B)-O(6B)	115.3(6)
C(7A)-C(8A)-H(8A)	118.9	O(5B)-S(2B)-O(4B)	113.1(5)
C(8A)-C(9A)-C(10A)	115.4(4)	O(6B)-S(2B)-O(4B)	118.8(5)
C(8A)-C(9A)-H(9A)	122.3	O(5B)-S(2B)-C(26)	107.5(5)
C(10A)-C(9A)-H(9A)	122.3	O(6B)-S(2B)-C(26)	99.5(5)
C(5A)-C(10A)-C(9A)	123.2(4)	O(4B)-S(2B)-C(26)	99.5(4)
C(5A)-C(10A)-N(2A)	105.7(4)		

Symmetry transformations used to generate equivalent atoms.

S2. DFT calculations

DFT gas phase calculations were performed using M06-2X[S-1] density functional with D3 dispersion correction[S-2] and the triple-zeta def2-TZVP[S-3] basis set on Gaussian 16.[S-4] The obtained data was read out using GoodVibes 3.0.1[S-5] and frequencies lower than 100 cm^{-1} were corrected according to and entropic, quasi harmonic approach (command: python -m goodvibes --imag *.out).[S-5,S-6]

S2.1.1. Coordinates of 3^S

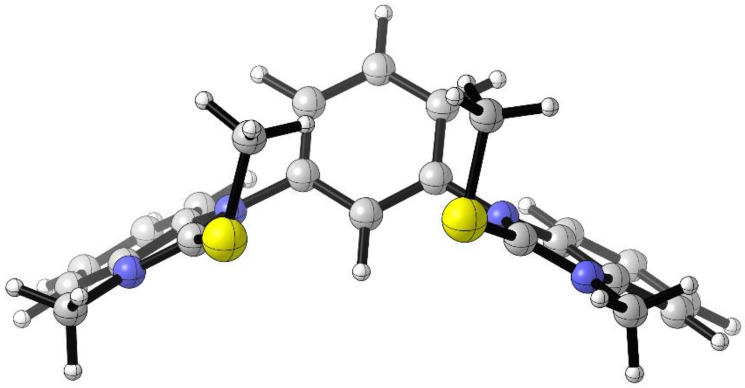

Figure S2 DFT calculation of **3**^{S}. Charge = +2. Latest E Energy = -1943.855782 ht. Graphics by CYLView.[S-7]

C	-1.24745300	0.74814100	2.20927300
C	-1.22966100	0.32871400	0.88590300
C	-0.03796900	0.15611200	0.19833600
C	1.14768900	0.38439600	0.88010900
C	1.15724100	0.81038300	2.20088200
C	-0.04894600	0.99753600	2.86176000
N	2.39274000	0.22472600	0.19145300
N	-2.47072700	0.10264700	0.21458400
C	2.91100800	-0.93560700	-0.26438800
N	4.06427800	-0.67871000	-0.88570000

C	4.31554500	0.68536600	-0.83143100
C	3.25738800	1.26528700	-0.13708200
C	-3.45250800	1.07843100	0.02313900
C	-4.44803200	0.48272300	-0.74375900
N	-4.05468800	-0.83190500	-0.96790000
C	-2.86928300	-1.03867400	-0.38937100
C	-3.53106200	2.40625000	0.42466900
C	-4.65825900	3.09837600	0.02653300
C	-5.66677300	2.49554300	-0.74251000
C	-5.58181900	1.17865600	-1.14756400
C	5.37174700	1.44275000	-1.32957300
C	5.31095100	2.80038100	-1.09525900
C	4.23901600	3.38559700	-0.39740700
C	3.19106900	2.63500100	0.09471000
C	-4.86151500	-1.79838200	-1.70816100
S	-1.99841500	-2.54014800	-0.48259000
S	2.18404100	-2.50911000	-0.06791600
C	4.96574200	-1.63747300	-1.51984200
C	-1.56053100	-2.81997900	1.25681400
C	2.55773400	-2.77576800	1.69412900
H	-2.19313900	0.87261200	2.72186600
H	-0.03161800	-0.14683600	-0.84028300
H	2.09983200	0.99237600	2.70223200
H	-0.05416000	1.32683400	3.89166500
H	-2.75245600	2.87665300	1.00874600
H	-4.76724600	4.13558900	0.31218300
H	-6.52995600	3.08098200	-1.02750200
H	-6.35437700	0.71901900	-1.74834100
H	6.19474600	0.99517300	-1.86952600
H	6.10757500	3.43499800	-1.45838800
H	4.23839600	4.45607600	-0.24420800
H	2.36521100	3.08585300	0.62739200
H	-4.46765900	-2.79616300	-1.54255000
H	-5.88449900	-1.74260800	-1.34223000
H	-4.83397300	-1.55817200	-2.76965000
H	5.03549300	-1.40918400	-2.58183500
H	5.94778500	-1.55287100	-1.05829200
H	4.57411700	-2.63992600	-1.38219500
H	-0.70546600	-2.21745700	1.55247700
H	-2.41782500	-2.63789100	1.89932300
H	-1.30402700	-3.87722100	1.30093000
H	3.63153900	-2.77379900	1.85879400
H	2.06110600	-2.03127400	2.31199200
H	2.16145500	-3.76449600	1.91898000

S2.1.2. Coordinates of 3^{Se}

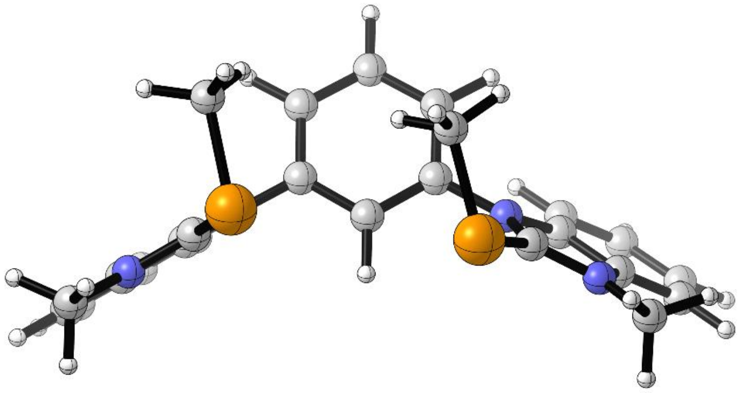

Figure S3 DFT calculation of **3**^{Se}. Charge = +2. Latest E Energy = -5950.634040 ht. Graphics by CYLView.[S-7]

C	-1.14065700	1.10640000	2.22934100
C	-1.12646100	0.72194300	0.89604000
C	0.06183400	0.50529900	0.21467900
C	1.25071100	0.64883000	0.91381300
C	1.26304100	1.02660400	2.24978100
C	0.06268200	1.26206600	2.90376900
N	2.49627000	0.44180600	0.24365500
N	-2.36887900	0.60978300	0.19178800
C	2.91820100	-0.69045500	-0.35846800
N	4.10948200	-0.46504700	-0.91575200
C	4.48377200	0.85367500	-0.67786500
C	3.46771700	1.43224500	0.07411200
C	-3.17182700	1.69320100	-0.15738200
C	-4.25084200	1.16460100	-0.85873900
N	-4.07228100	-0.21254700	-0.89579700
C	-2.94247400	-0.52501700	-0.25827500
C	-3.03521700	3.05892400	0.06620400
C	-4.03556900	3.86059300	-0.44433600
C	-5.12819300	3.32761100	-1.15153800
C	-5.25887300	1.97300200	-1.37594400
C	5.61520200	1.56689300	-1.05772700
C	5.67586900	2.88224700	-0.64317100
C	4.64705100	3.46712800	0.11270400
C	3.52213000	2.75796800	0.48644100
C	-5.02262000	-1.12094900	-1.53297400
C	4.94266100	-1.41337100	-1.64989100
C	-2.67873300	-2.39938900	1.90732300
C	1.47457900	-2.51271400	1.41242000
H	-2.08502400	1.28483900	2.72869100
H	0.05920900	0.23557300	-0.83318300
H	2.20695000	1.13168300	2.77008400
H	0.06409800	1.55957700	3.94333300
H	-2.19370100	3.46854300	0.60767500
H	-3.98022800	4.93062400	-0.29821400
H	-5.88527500	4.00067200	-1.52967800
H	-6.09811500	1.56594700	-1.92288700

H	6.40406700	1.12198400	-1.64826900
H	6.53608400	3.48054900	-0.90986300
H	4.73845000	4.50343700	0.40764800
H	2.72794000	3.21306400	1.06169600
H	-4.70910700	-2.14539600	-1.36163600
H	-6.00727800	-0.95593000	-1.09962000
H	-5.04984500	-0.91443200	-2.60139100
H	4.90416600	-1.18691400	-2.71407800
H	5.96481400	-1.32203900	-1.28904200
H	4.58378800	-2.42214200	-1.46975400
H	-2.13193800	-1.64342100	2.46340700
H	-3.75066500	-2.30880600	2.04909200
H	-2.35140800	-3.39365000	2.20383900
H	2.31966700	-2.26872300	2.04899700
H	0.60884300	-1.89441800	1.63067200
H	1.23050000	-3.56695600	1.52618100
Se	-2.24931000	-2.27765600	-0.00230400
Se	1.98983800	-2.33927500	-0.46631000

S2.1.3. Coordinates of 3Te

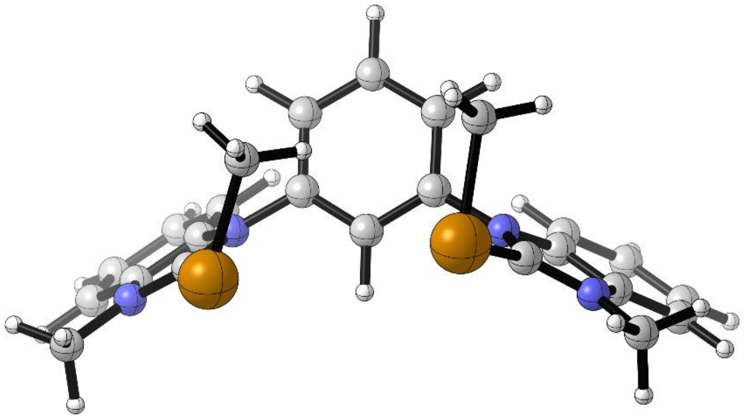

Figure S4 DFT calculation of **3Te**. Charge = +2. Latest E Energy = -1683.359488 ht. Graphics by CYLView.[S-7]

C	-1.29325000	1.12842700	2.31539300
C	-1.29386800	0.86007300	0.95323300
C	-0.11182300	0.79017400	0.23087900
C	1.08298100	0.97396600	0.91186600
C	1.10798100	1.24807900	2.27206200
C	-0.08790300	1.32491400	2.97300300
N	2.31590500	0.97379500	0.18064600
N	-2.54694400	0.70336400	0.28224400
C	2.99411400	-0.09819100	-0.28024400
N	4.07606200	0.33850200	-0.93185700
C	4.11119900	1.72688000	-0.89408700
C	2.99430000	2.13714700	-0.17523800
C	-3.49249200	1.72458000	0.16122900
C	-4.53731800	1.19579400	-0.58700100
N	-4.20037400	-0.12440300	-0.86946000
C	-3.00480300	-0.40272000	-0.34262200
C	-3.50464600	3.03680700	0.61808800
C	-4.61720400	3.78653600	0.28954600
C	-5.67417900	3.25308100	-0.46569500
C	-5.65610400	1.95038000	-0.92246600
C	5.02135000	2.63861400	-1.42121600
C	4.75373800	3.97138600	-1.18672300
C	3.62347200	4.38441400	-0.45900600
C	2.72037800	3.47992400	0.06111000
C	-5.07134700	-1.02251800	-1.62074500
C	5.11173900	-0.45372200	-1.58974400
C	-1.33883800	-2.32926700	1.52333000
C	2.80368200	-2.07816900	2.12616100
H	-2.23250900	1.18335200	2.85172200
H	-0.11993300	0.61032400	-0.83642100
H	2.05595500	1.40956800	2.77029200
H	-0.07983800	1.53830500	4.03304600
H	-2.68926000	3.45034900	1.19525800
H	-4.67720700	4.81417400	0.62062700
H	-6.52304600	3.88162900	-0.69687300
H	-6.46778000	1.54598300	-1.51122600

H	5.89016500	2.32450700	-1.98306900
H	5.43062600	4.72137600	-1.57208700
H	3.46032900	5.44209100	-0.30391300
H	1.85068800	3.79589900	0.62062000
H	-4.76191800	-2.05060600	-1.45454700
H	-6.09001300	-0.89526400	-1.26091500
H	-5.02246600	-0.78293300	-2.68172900
H	5.07712000	-0.26958100	-2.66213900
H	6.08140900	-0.15534100	-1.19575600
H	4.94417200	-1.50692500	-1.38921400
H	-0.45262500	-1.71306900	1.64573100
H	-2.14297400	-2.02122700	2.18502800
H	-1.10605100	-3.37658200	1.70798400
H	3.83224500	-1.78551300	2.30937800
H	2.10025200	-1.41339500	2.61764800
H	2.64999600	-3.10135500	2.46436700
Te	-2.01979300	-2.26665000	-0.50030500
Te	2.43581500	-2.12266000	0.01854600

S3. NMR spectra

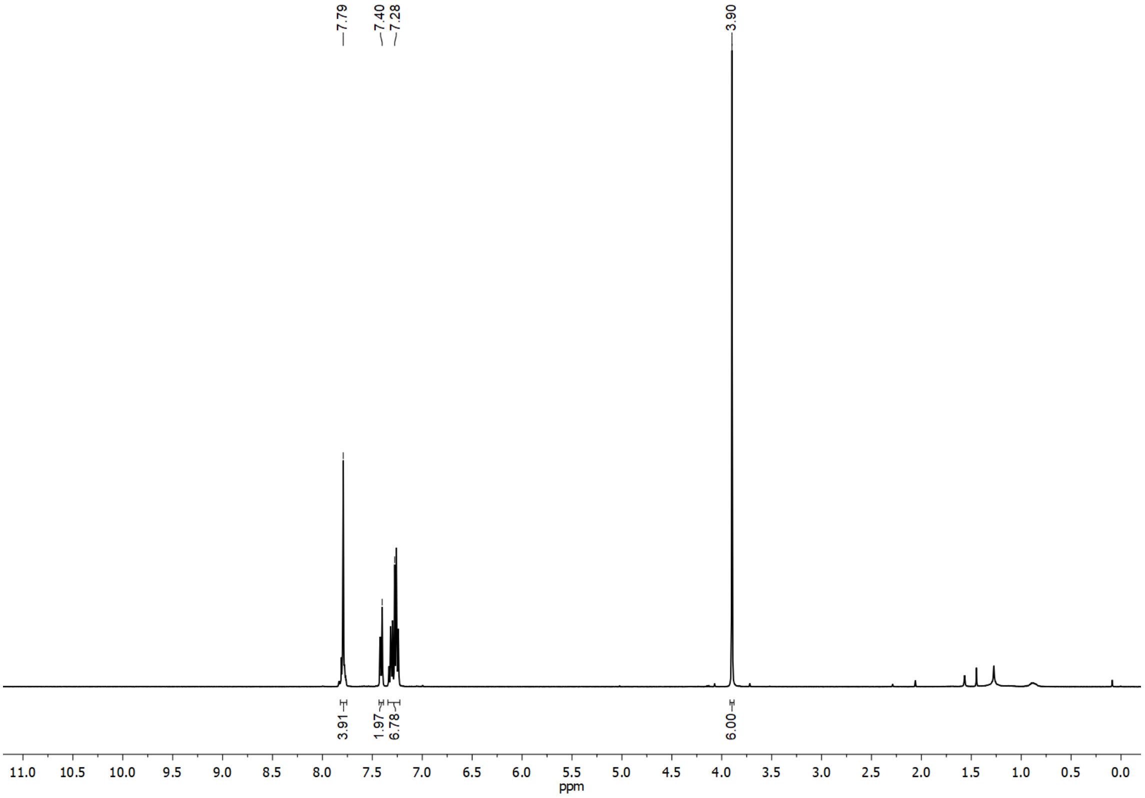

Figure S5 ^{1}H NMR spectrum of **2**$^{\mathbf{S}}$ in $CDCl_3$.

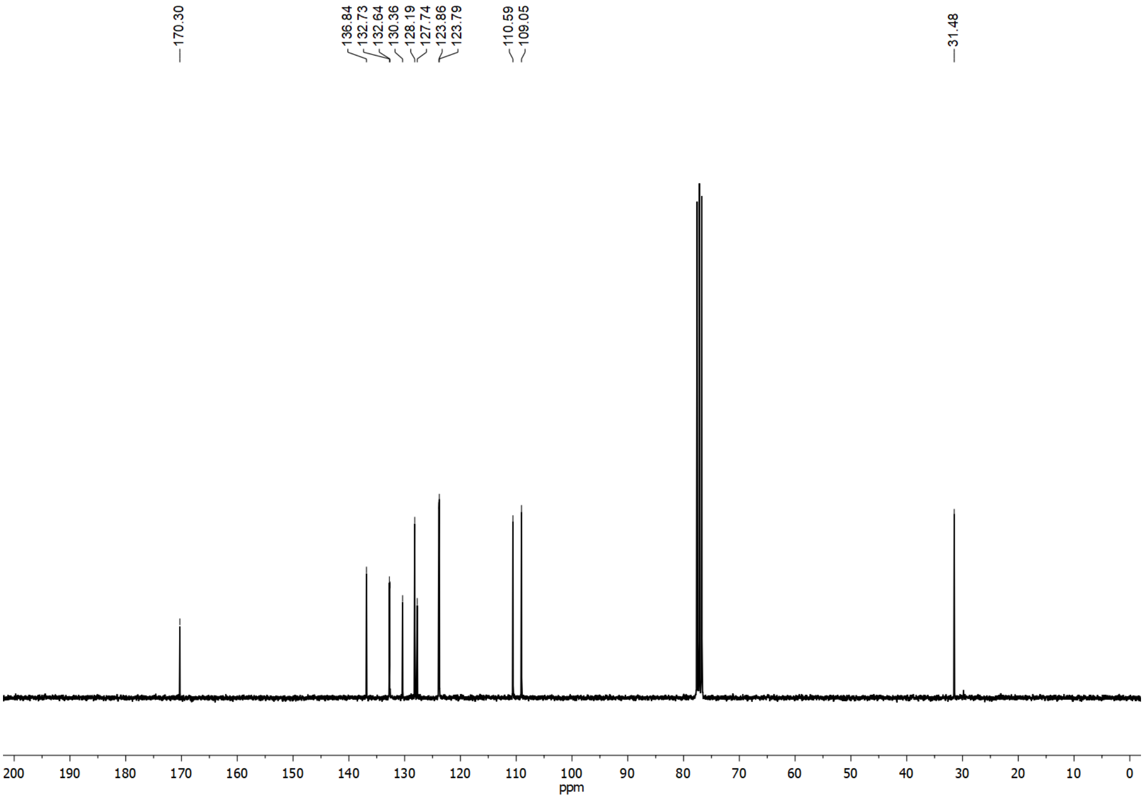

Figure S6 ^{13}C NMR spectrum of **2**$^{\mathbf{S}}$ in $CDCl_3$

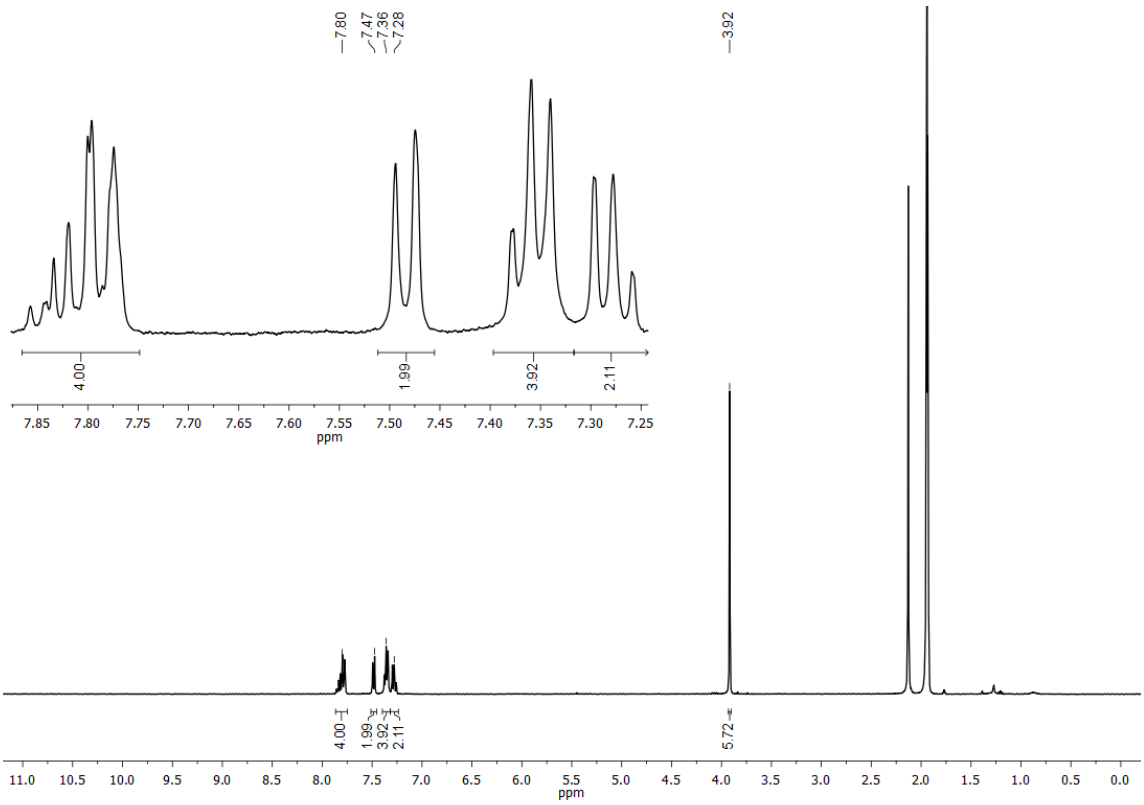

Figure S7 ^{1}H NMR spectrum of **2**$^{\mathbf{Se}}$ in CD_3CN.

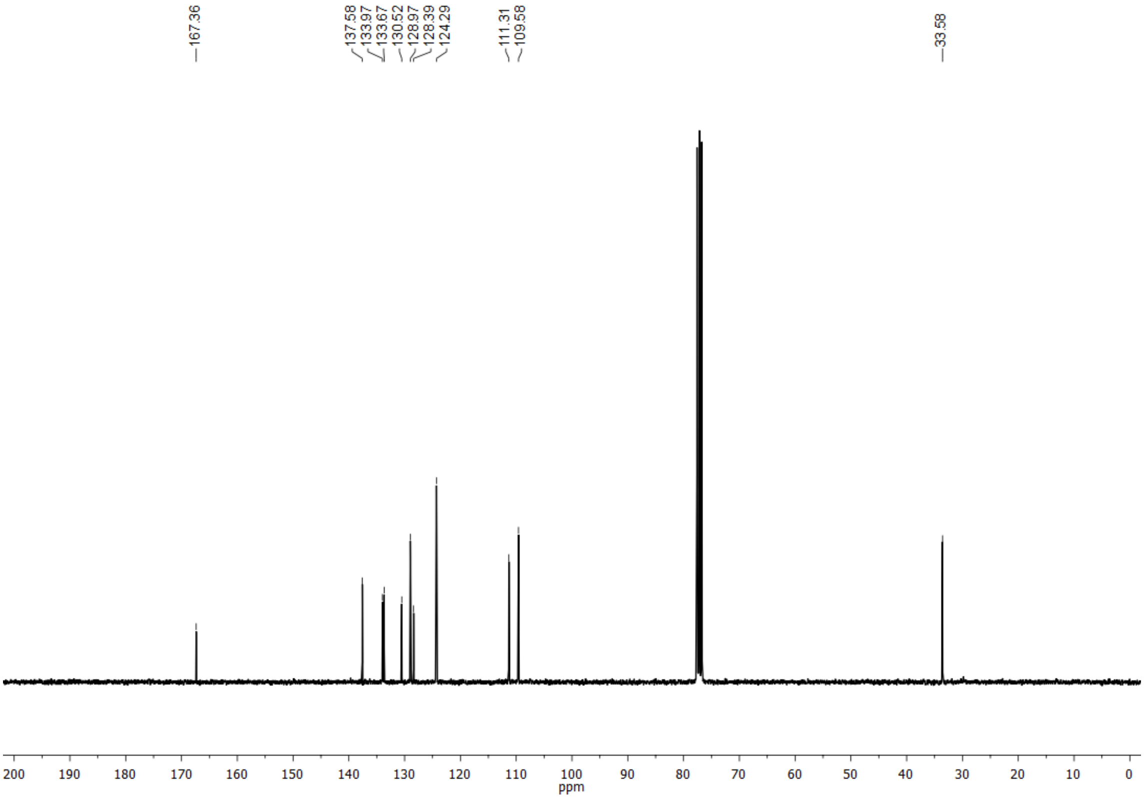

Figure S8 ^{13}C NMR spectrum of **2**$^{\mathbf{Se}}$ in $CDCl_3$.

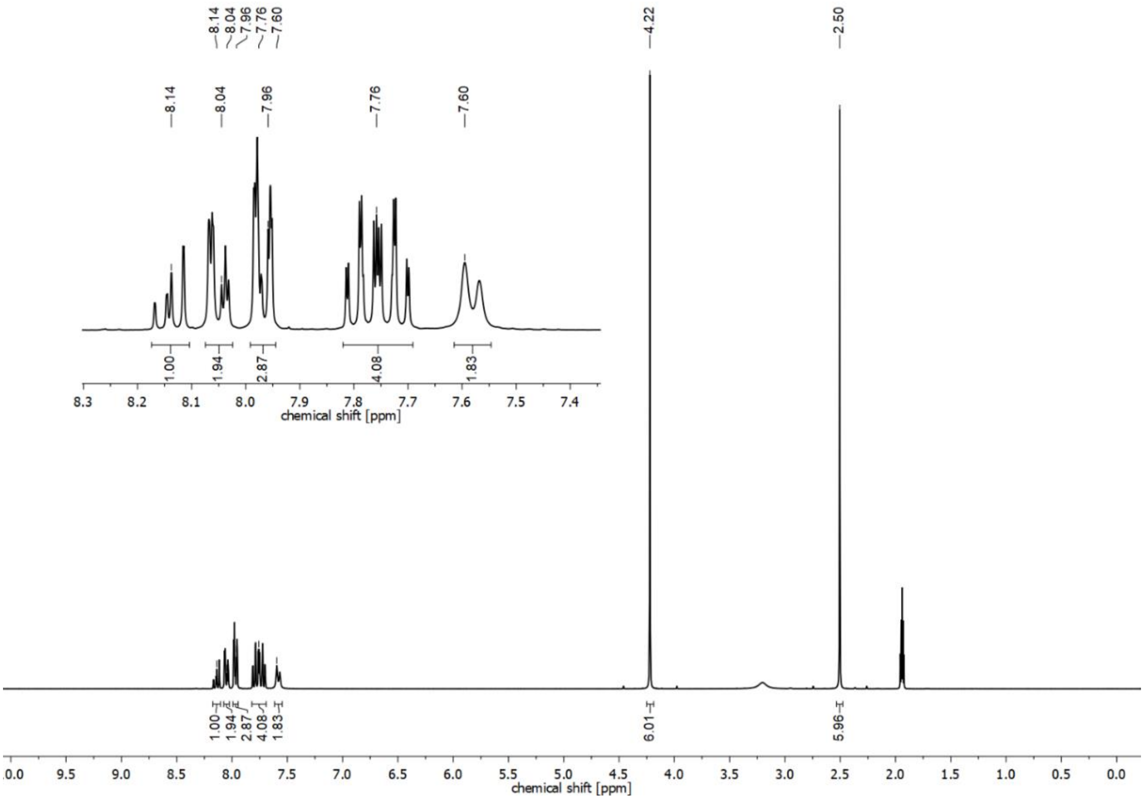

Figure S9 ^{1}H NMR spectrum of **3^{S}** in CD_3CN.

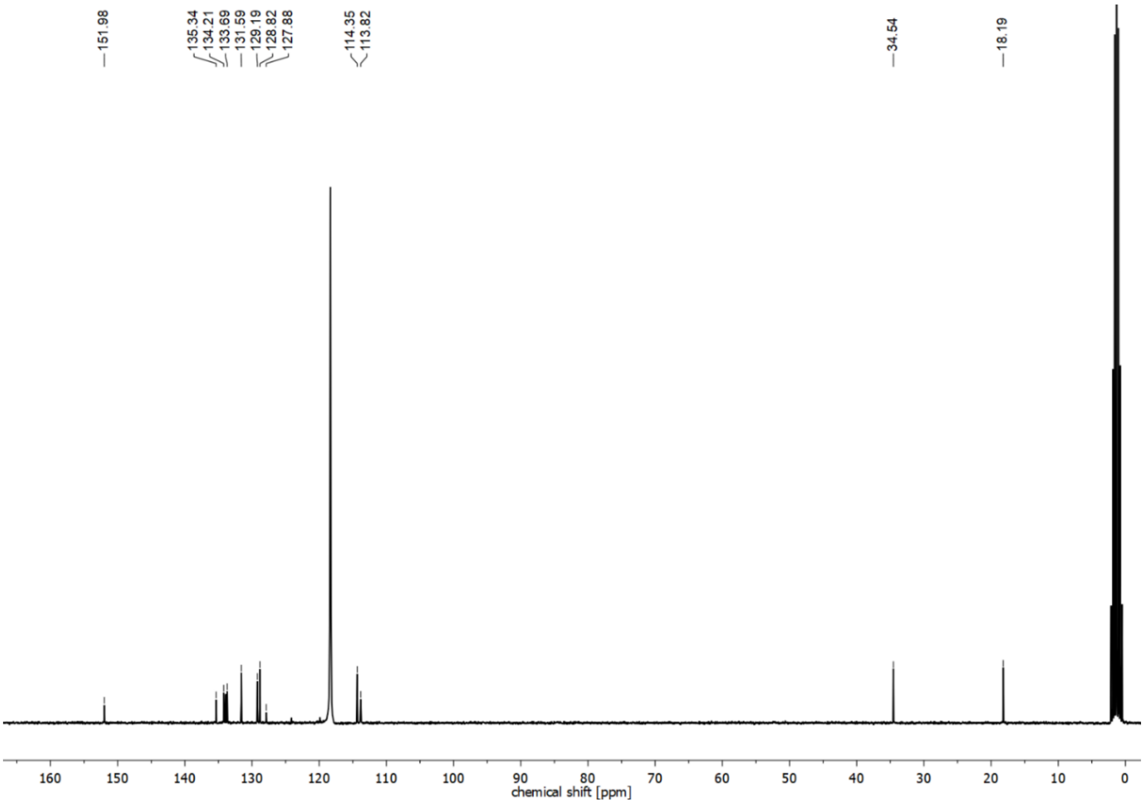

Figure S10 ^{13}C NMR spectrum of **3^{S}** in CD_3CN.

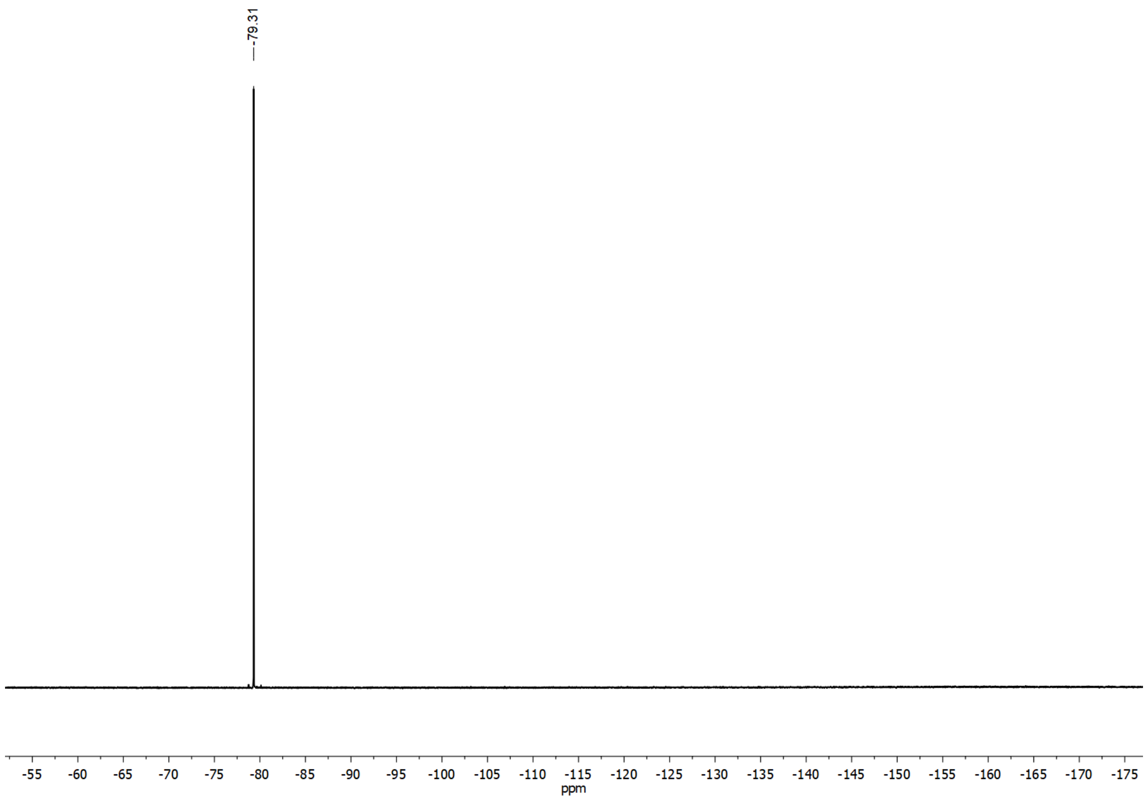

Figure S11 ^{19}F NMR spectrum of $\mathbf{3^S}$ in CD_3CN.

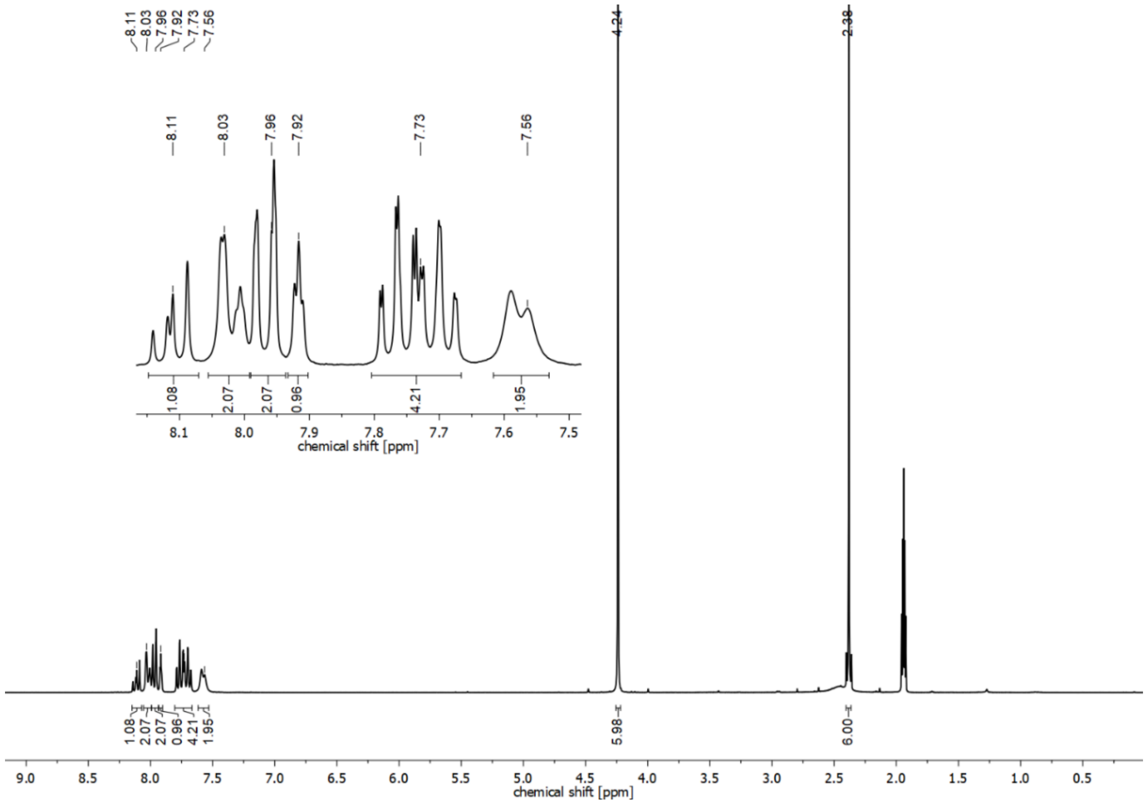

Figure S12 ^{1}H NMR spectrum of $\mathbf{3^{Se}}$ in CD_3CN.

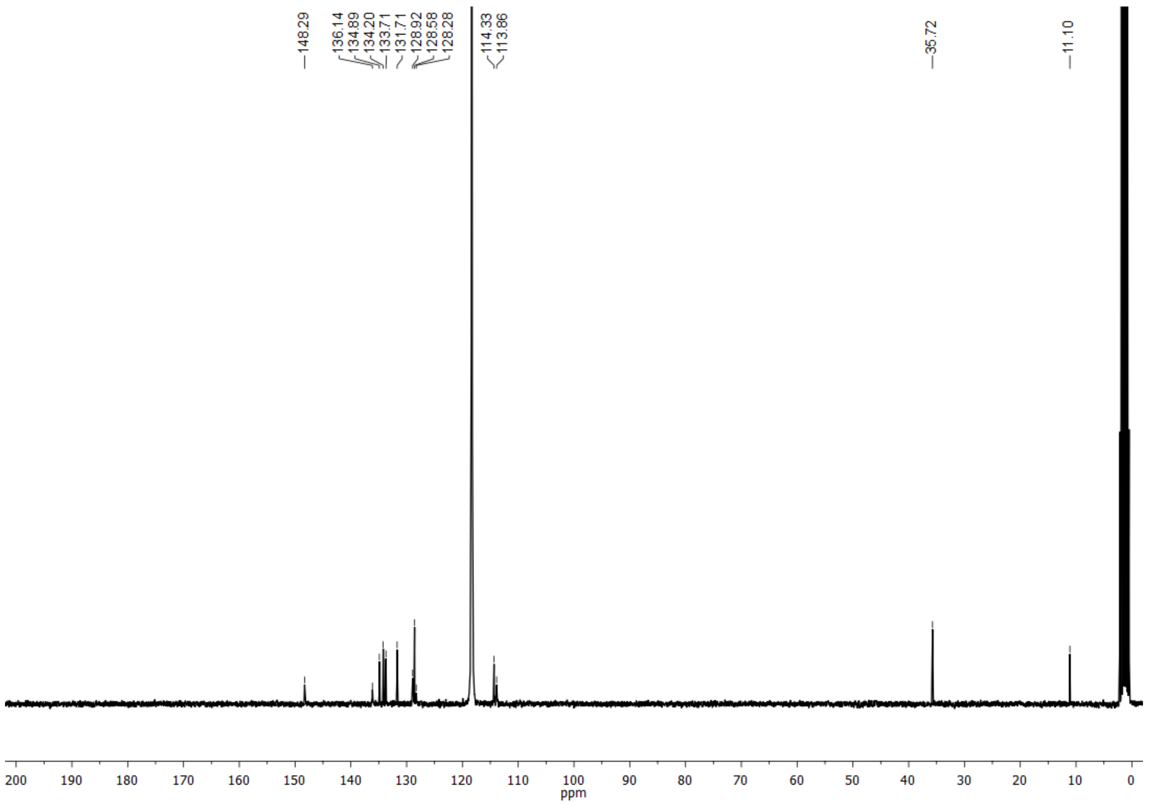

Figure S13 ^{13}C NMR spectrum of $\mathbf{3^{Se}}$ in CD_3CN.

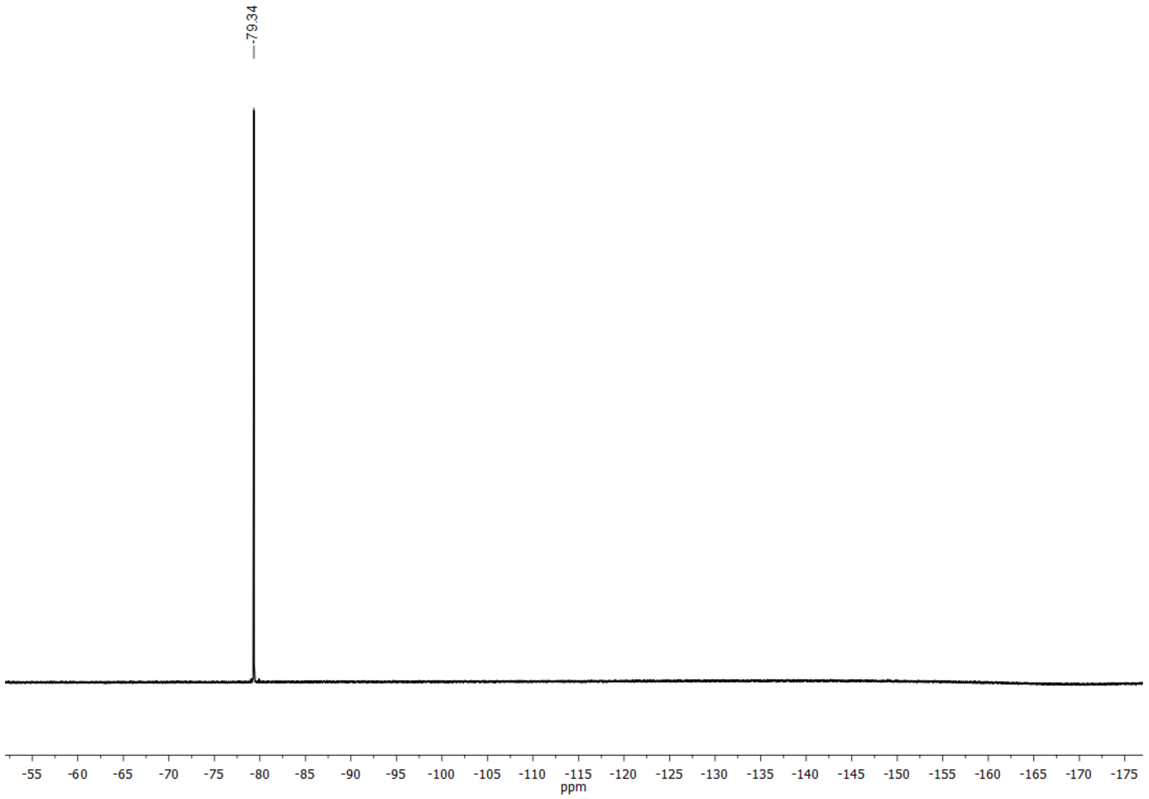

Figure S14 ^{19}F NMR spectrum of $\mathbf{3^{S}}$ in $CDCl_3$.

S4. SI references

[S-1] Y. Zhao, D. G. Truhlar, *Theor. Chem. Acc*. **2008**, *120*, 215-241.

[S-2] S. Grimme, *Chem. Eur. J*. **2012**, *18*, 9955-9964.

[S-3] a) F. Weigend, R. Ahlrichs, *Phys. Chem. Chem. Phys*. **2005**, *7*, 3297–3305.

[S-4] Gaussian 16, Revision C.01, M. J. Frisch, G. W. Trucks, H. B. Schlegel, G. E. Scuseria, M. A. Robb, J. R. Cheeseman, G. Scalmani, V. Barone, G. A. Petersson, H. Nakatsuji, X. Li, M. Caricato, A. V. Marenich, J. Bloino, B. G. Janesko, R. Gomperts, B. Mennucci, H. P. Hratchian, J. V. Ortiz, A. F. Izmaylov, J. L. Sonnenberg, D. Williams-Young, F. Ding, F. Lipparini, F. Egidi, J. Goings, B. Peng, A. Petrone, T. Henderson, D. Ranasinghe, V. G. Zakrzewski, J. Gao, N. Rega, G. Zheng, W. Liang, M. Hada, M. Ehara, K. Toyota, R. Fukuda, J. Hasegawa, M. Ishida, T. Nakajima, Y. Honda, O. Kitao, H. Nakai, T. Vreven, K. Throssell, J. A. Montgomery, Jr., J. E. Peralta, F. Ogliaro, M. J. Bearpark, J. J. Heyd, E. N. Brothers, K. N. Kudin, V. N. Staroverov, T. A. Keith, R. Kobayashi, J. Normand, K. Raghavachari, A. P. Rendell, J. C. Burant, S. S. Iyengar, J. Tomasi, M. Cossi, J. M. Millam, M. Klene, C. Adamo, R. Cammi, J. W. Ochterski, R. L. Martin, K. Morokuma, O. Farkas, J. B. Foresman, and D. J. Fox, Gaussian, Inc., Wallingford CT, **2019**.

[S-5] G. Luchini, J. Alegre-Requena, IFunes, J. Rodríguez-Guerra, J. Chen, R. Paton, bobbypaton/GoodVibes: GoodVibes v3.0.0, Zenodo, **2019**

[S-6] S. Grimme, *Chem. Eur. J*. **2012**, *18*, 9955– 9964.

[S-7] C. Y. Legault. *CYLview*, *1.0b*. http://www.cylview.org; C. Y. Legault: Université de Sherbrooke, **2009**.
